# Supplementary material for: Phenoxyaromatic Acid Analogues as Novel Radiotherapy Sensitizers: Design, Synthesis and Biological Evaluation
Source: Molecules. 2022 Apr 9;27(8):2428. doi: 10.3390/molecules27082428 (PMC9024523; doi:10.3390/molecules27082428)
Supplement: Supplementary file 1 [file molecules-27-02428-s001.zip › Figure S1.pdf]

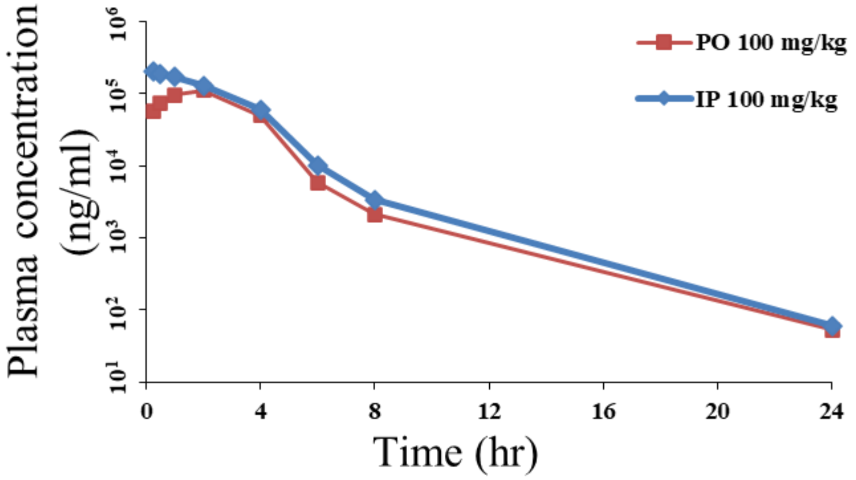

Figure S1. Pharmacokinetic Study in Rats. Compound **19c** was administrated at a dosage of 100 mg/kg by intraperitoneal injection (IP) or oral administration (PO). Plasma concentration vs time profile for **19c** after drug administration in rats.
