# Supplementary figures and images for: Phenoxyaromatic Acid Analogues as Novel Radiotherapy Sensitizers: Design, Synthesis and Biological Evaluation
Source: Molecules. 2022 Apr 9;27(8):2428. doi: 10.3390/molecules27082428 (PMC9024523; doi:10.3390/molecules27082428)

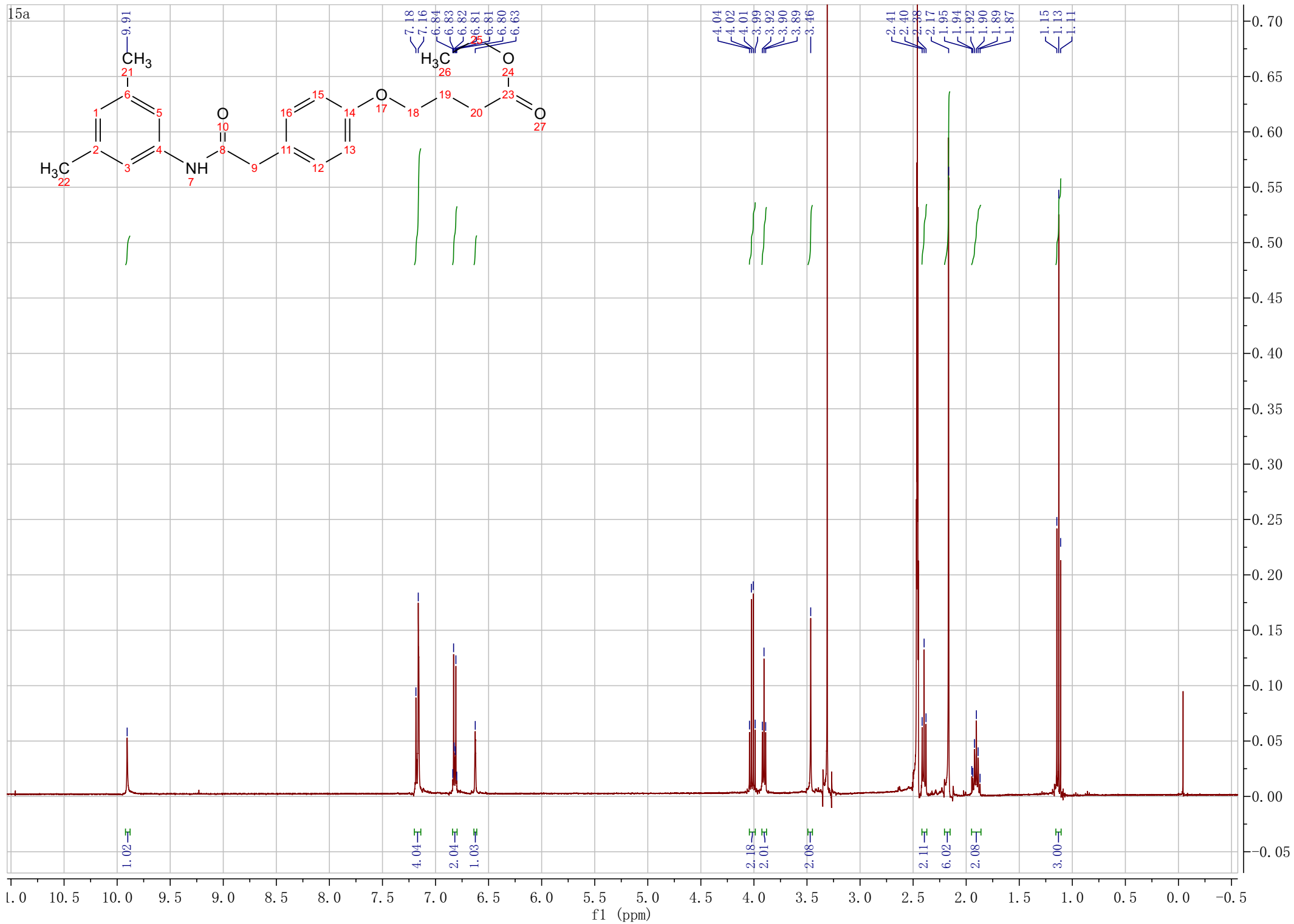

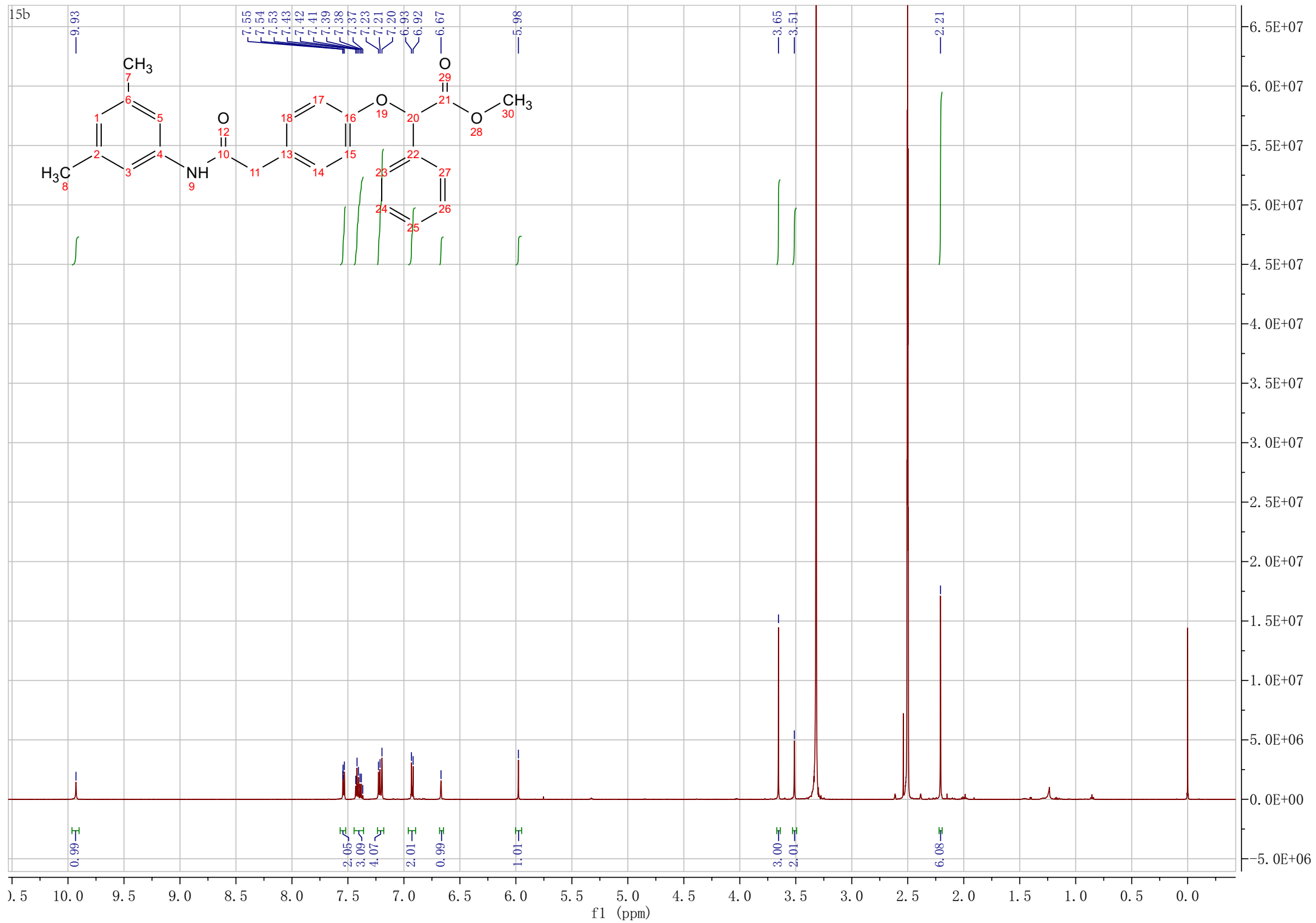

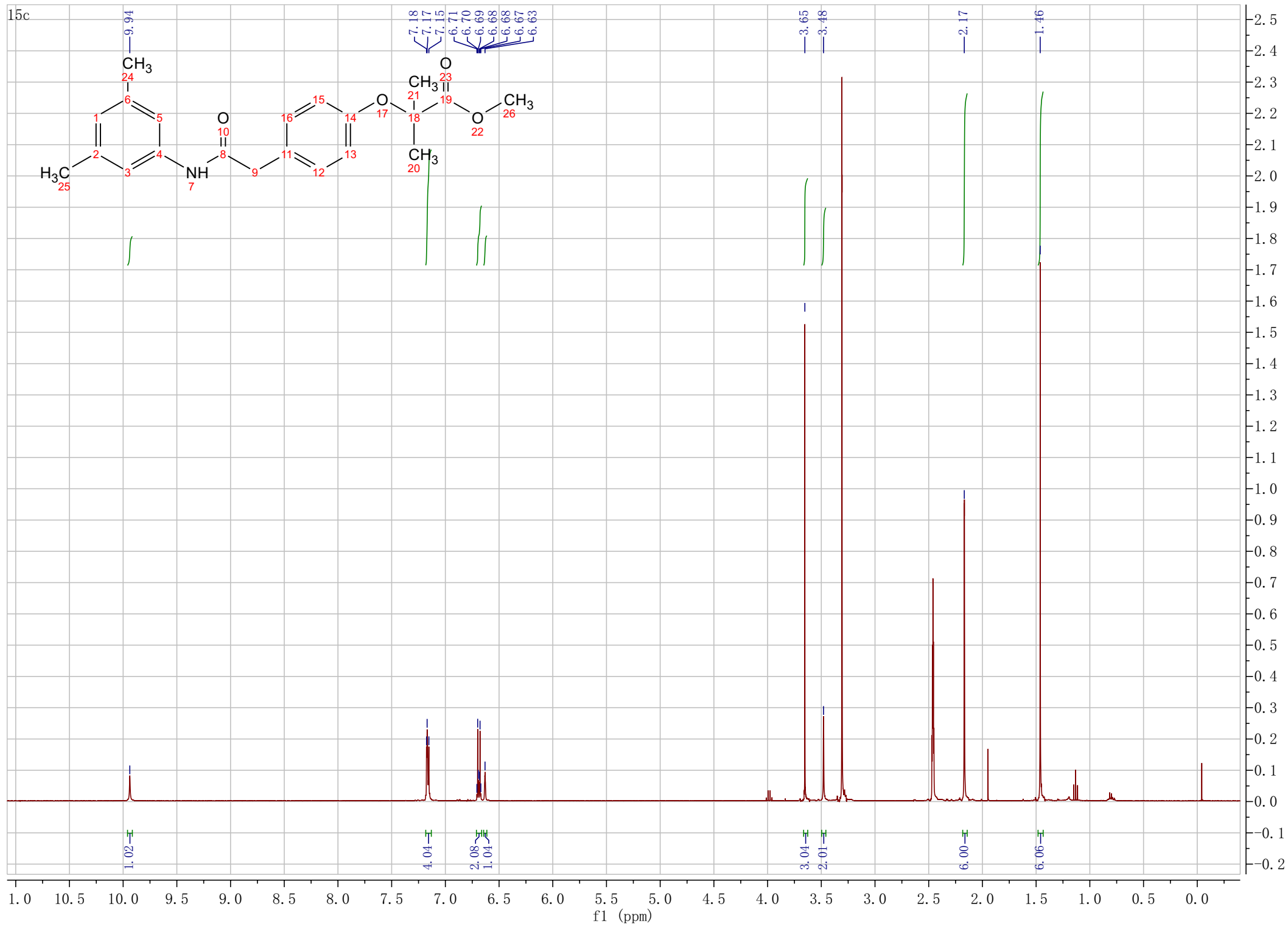

16a

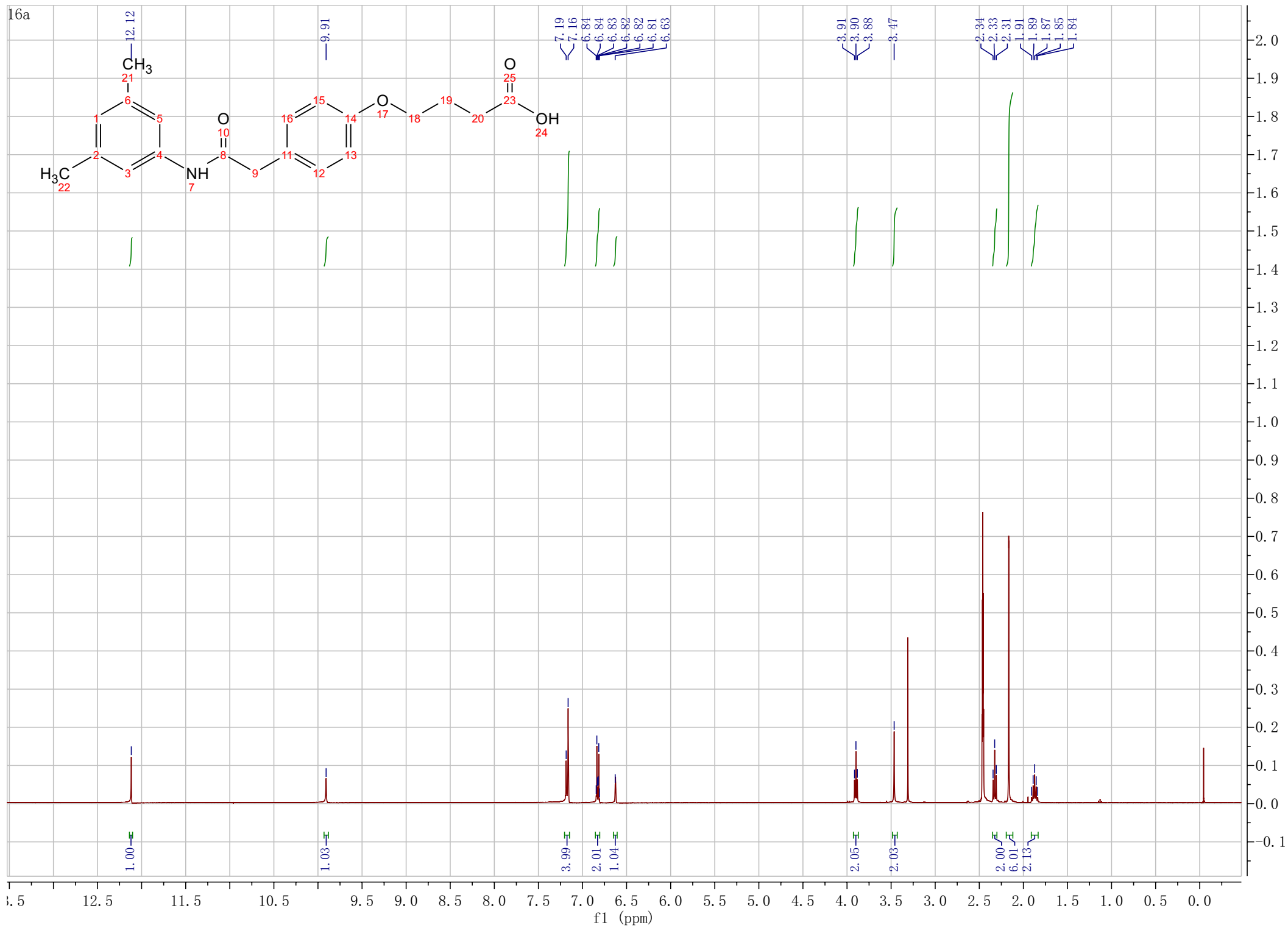

16b

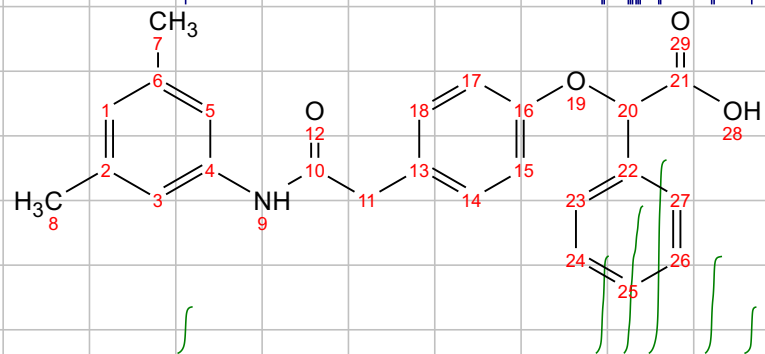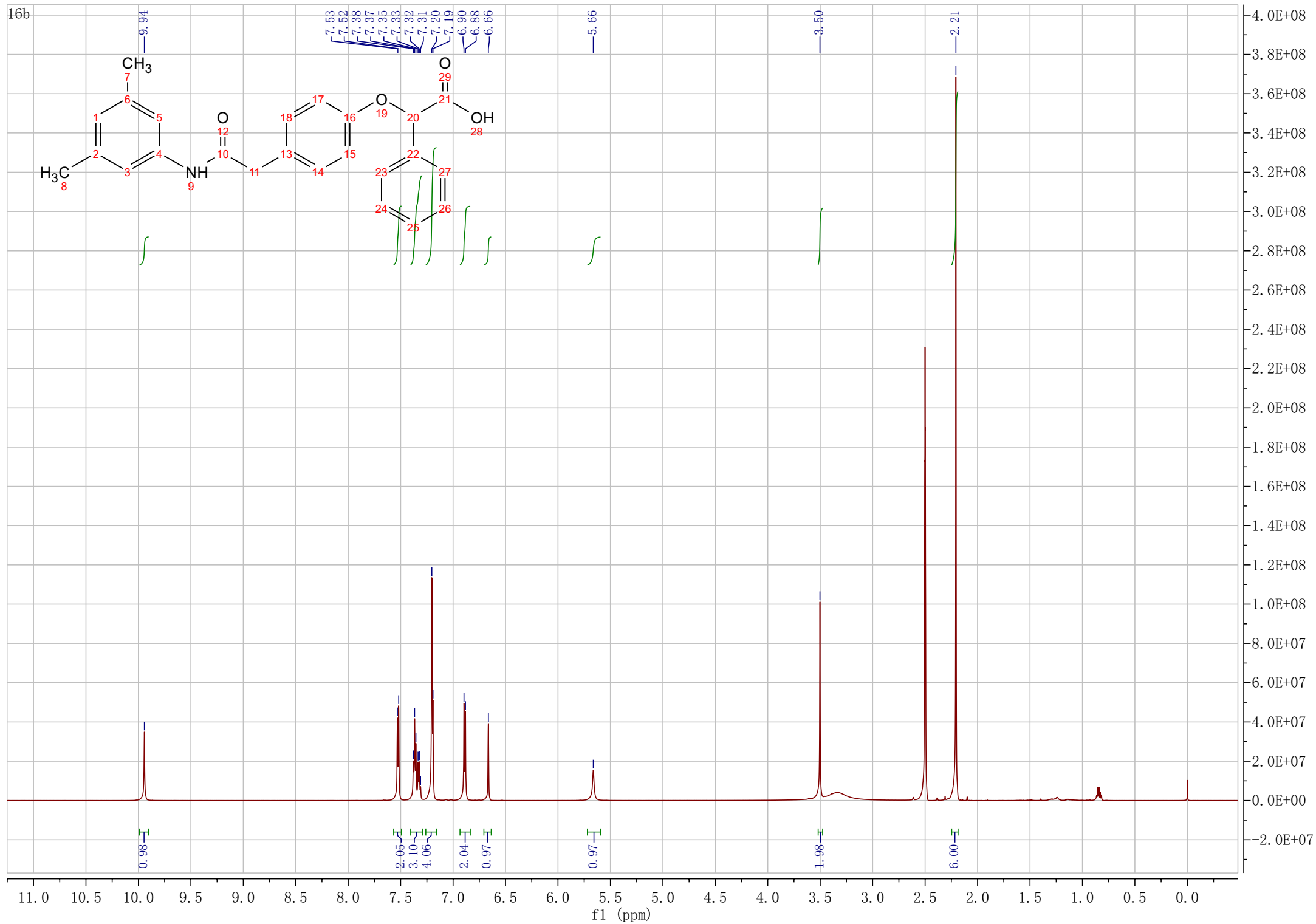

18a

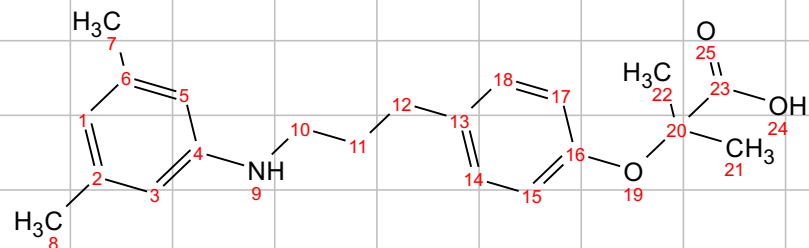

7.09  
7.08  
6.94  
6.77  
6.76  
6.75  
6.74

3.19  
3.18  
3.16  
2.60  
2.59  
2.58  
2.27  
1.91  
1.90  
1.88  
1.87  
1.86  
1.48

16

15

14

13

12

11

10

9

8

7

f1 (ppm)

5

4

3

2

1

0

-1

-2

-3

-4

-1.00E+07

0.00E+00

1.00E+07

2.00E+07

3.00E+07

4.00E+07

5.00E+07

6.00E+07

7.00E+07

8.00E+07

9.00E+07

1.00E+08

1.10E+08

1.20E+08

1.30E+08

1.40E+08

1.50E+08

1.60E+08

1.70E+08

18b

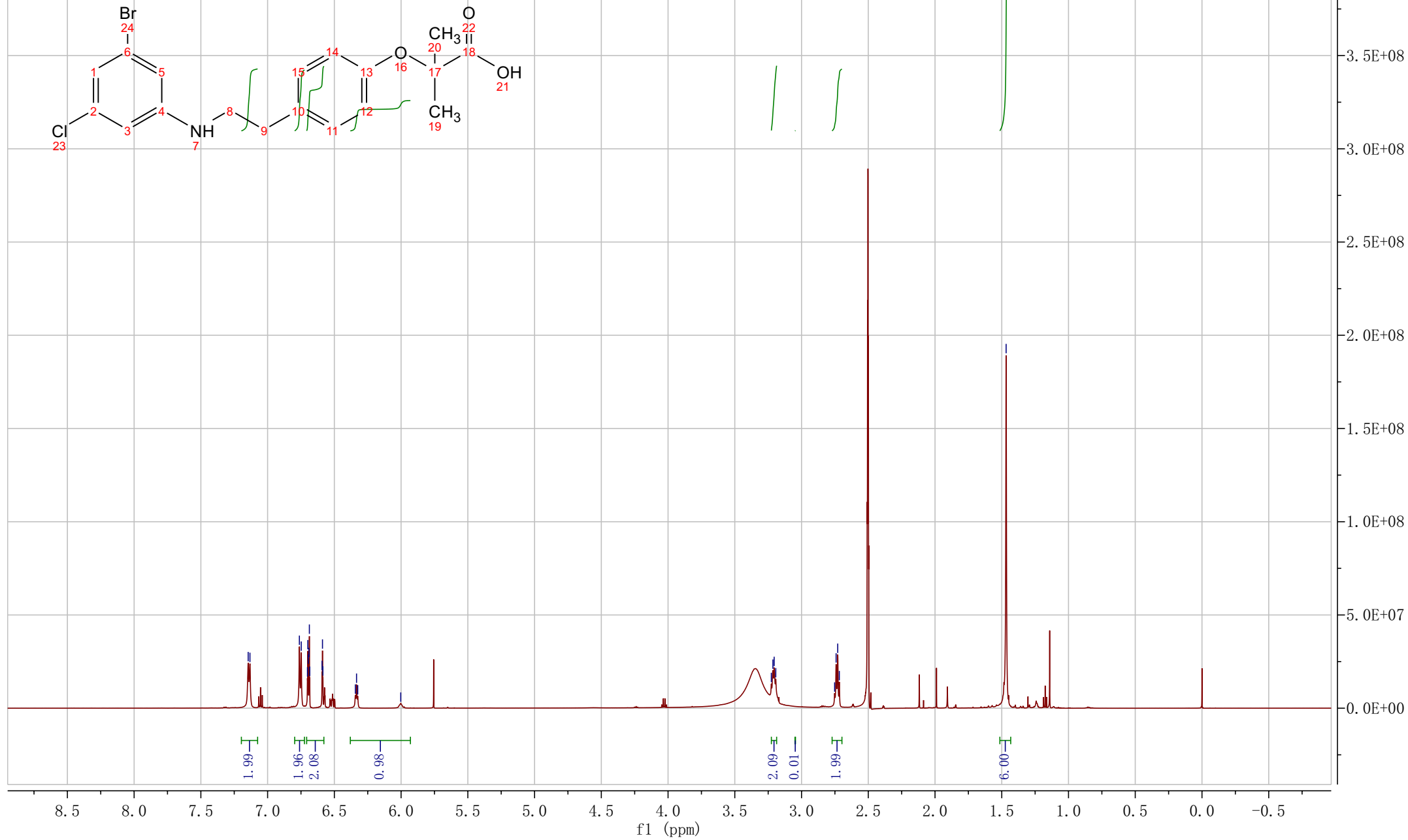

18c

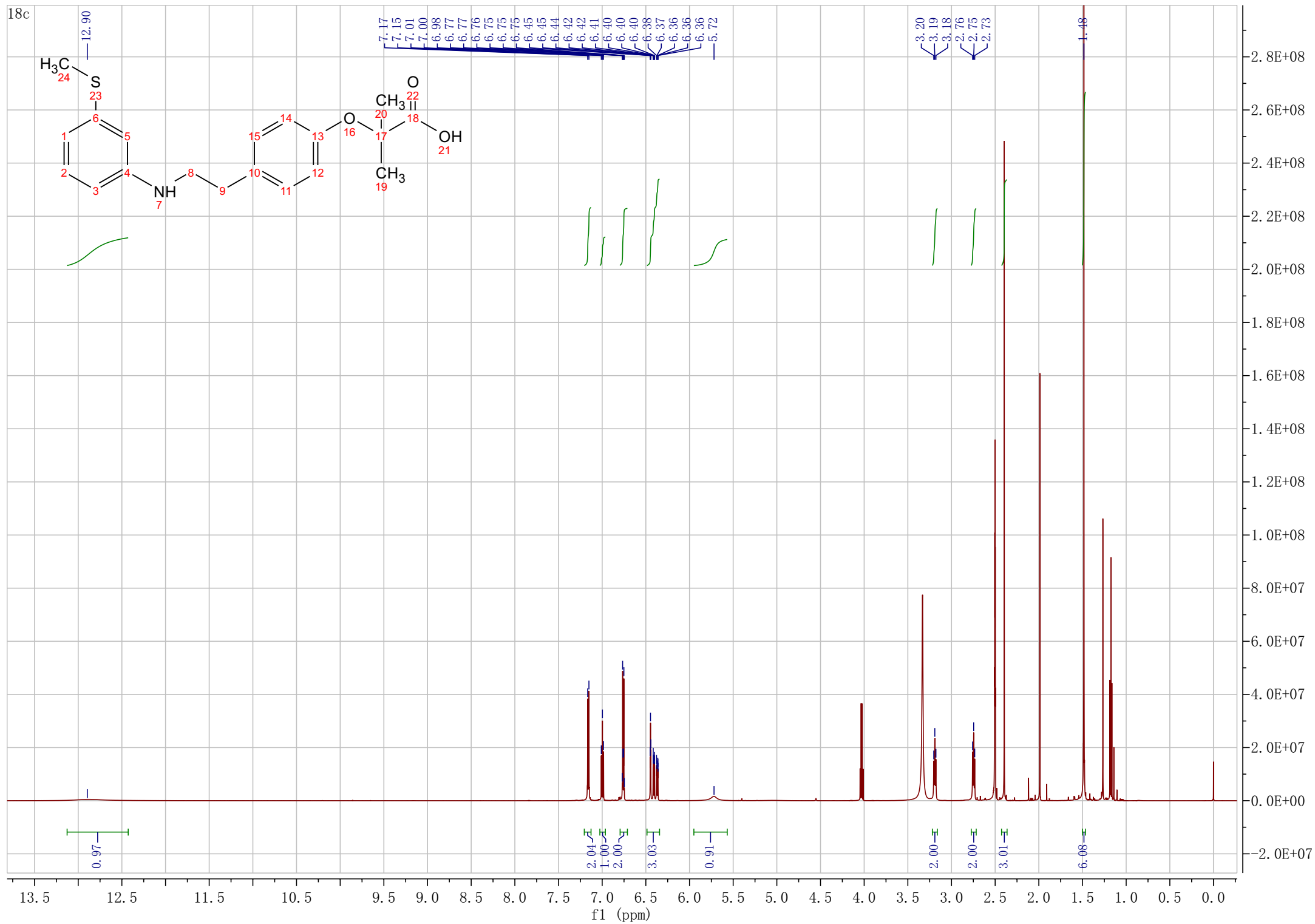

18d

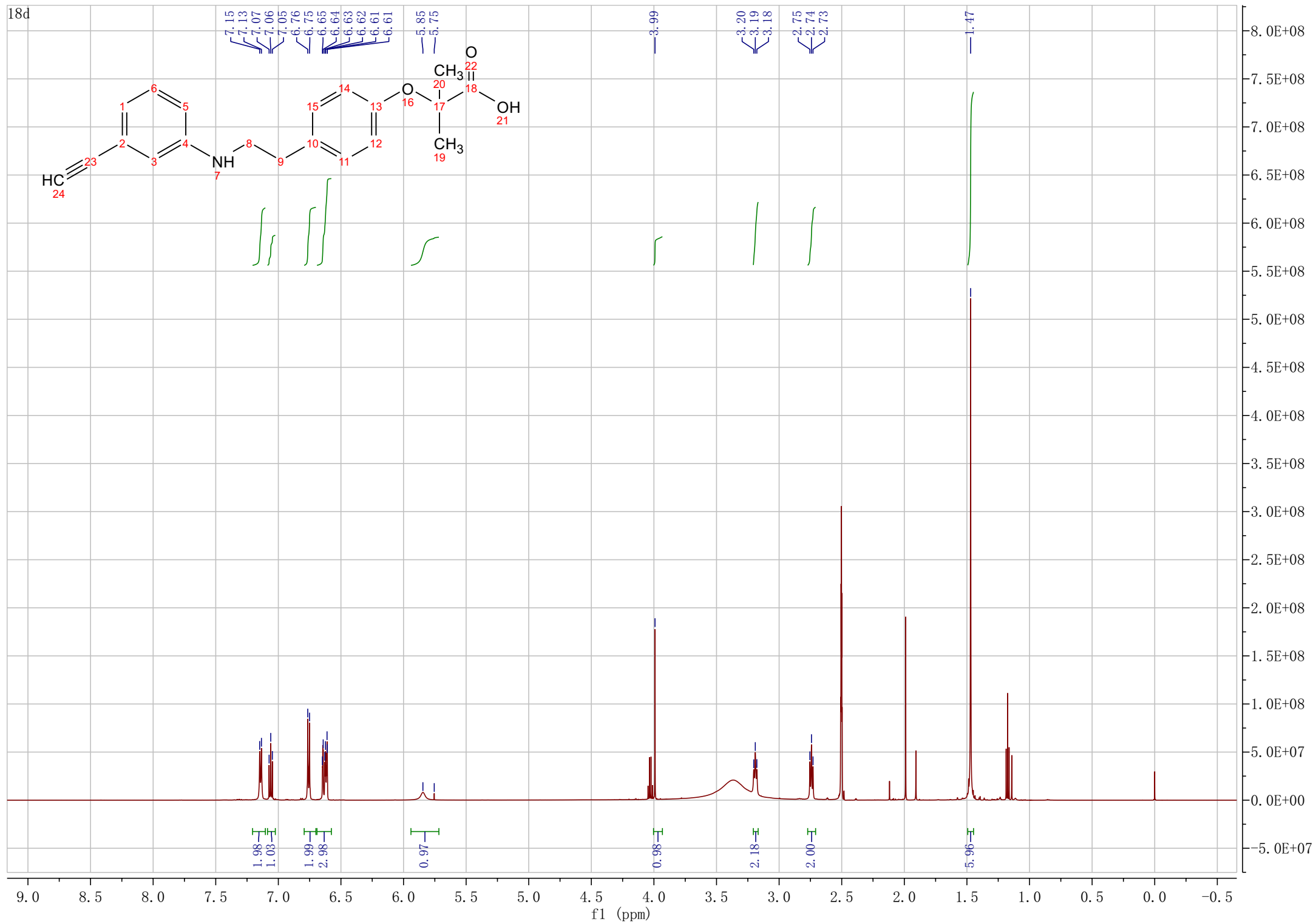

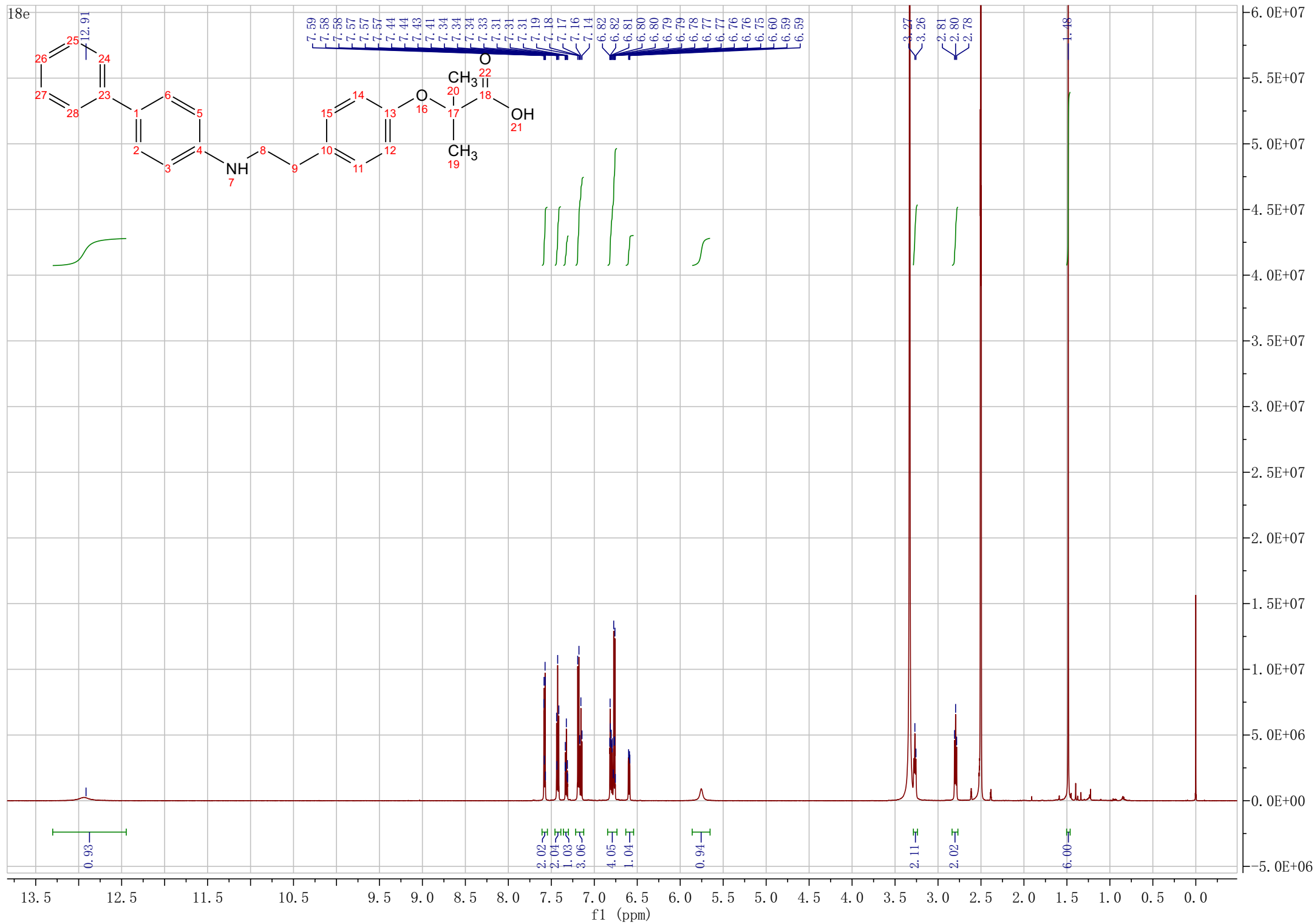

18f

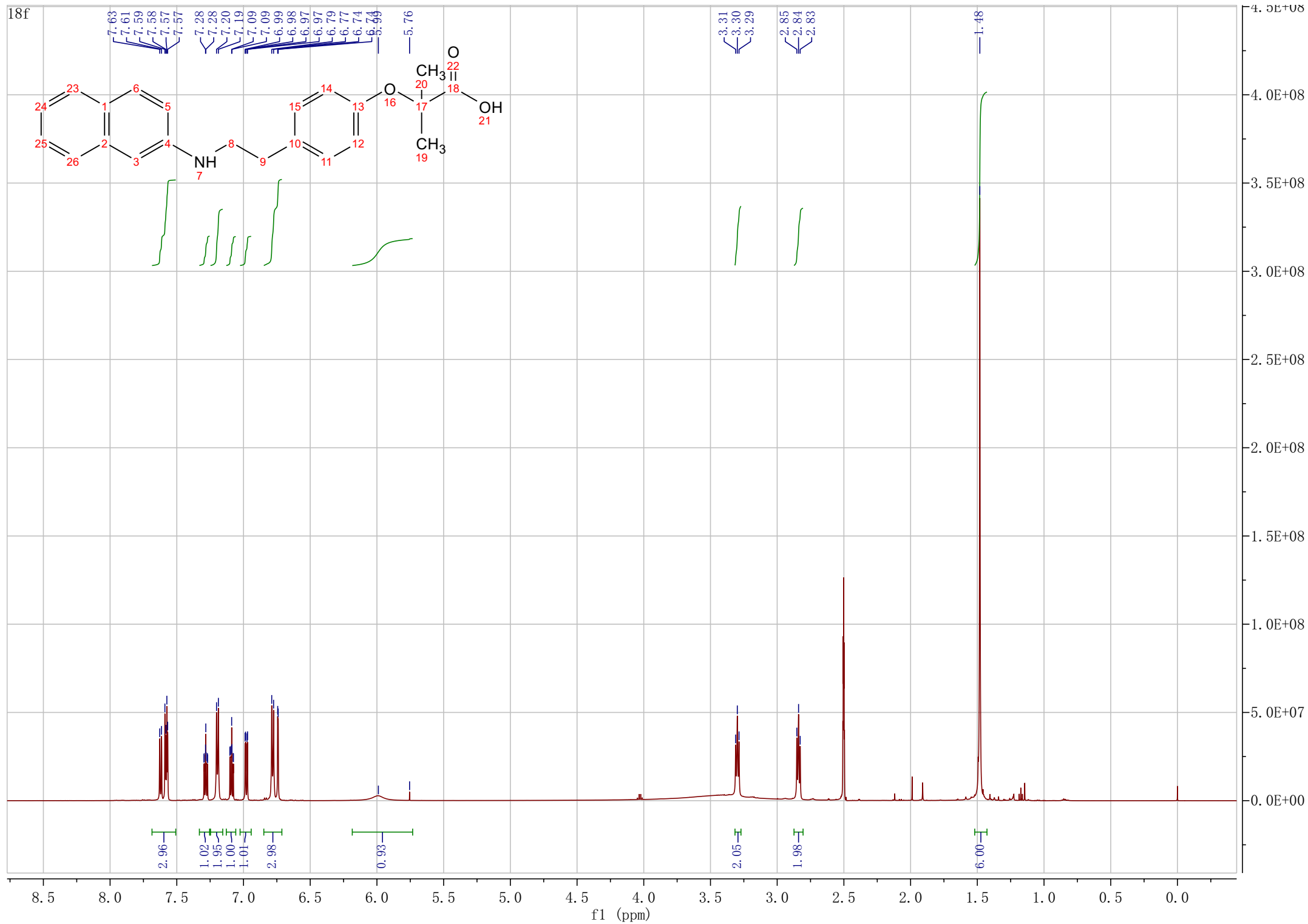

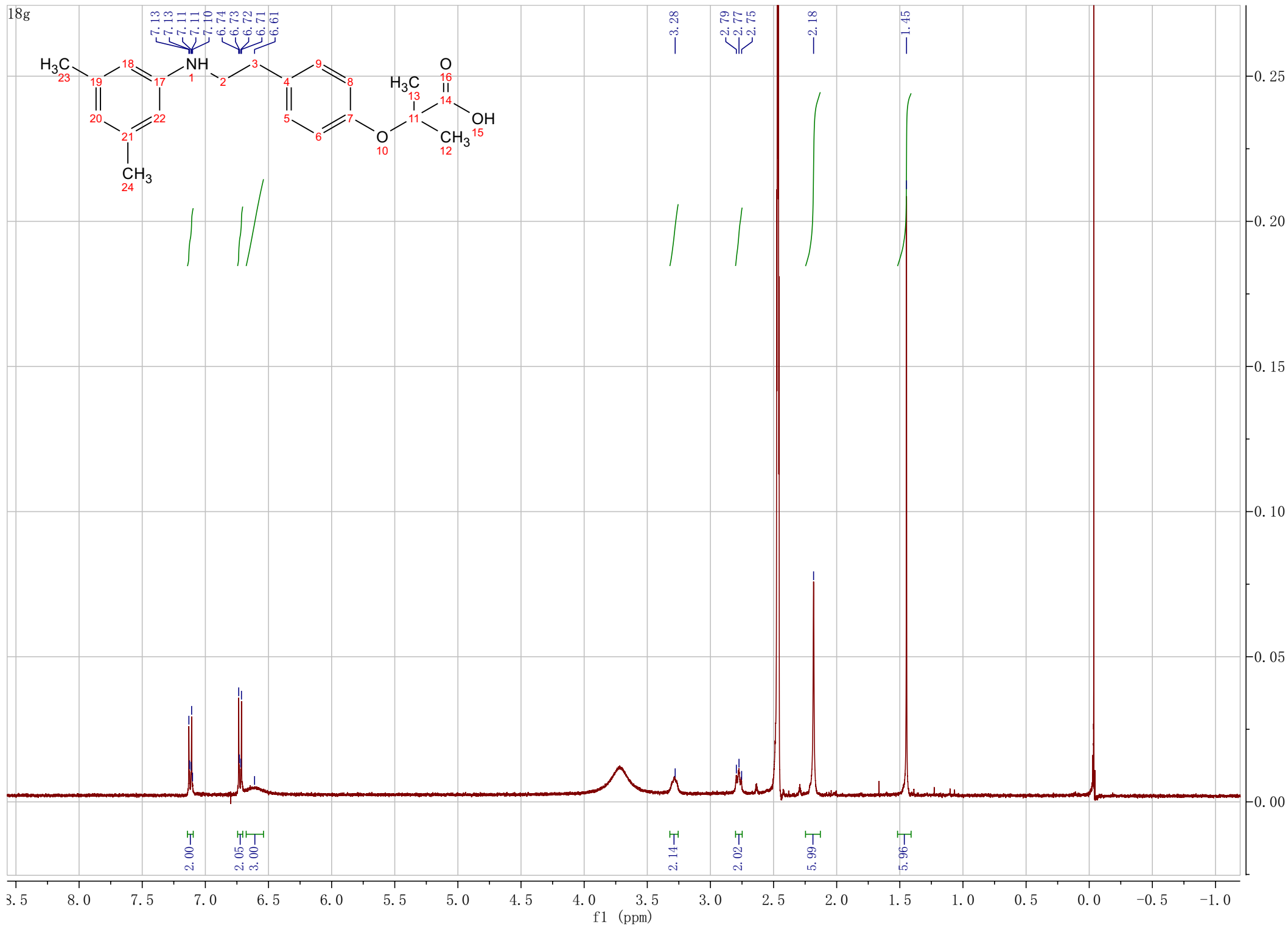

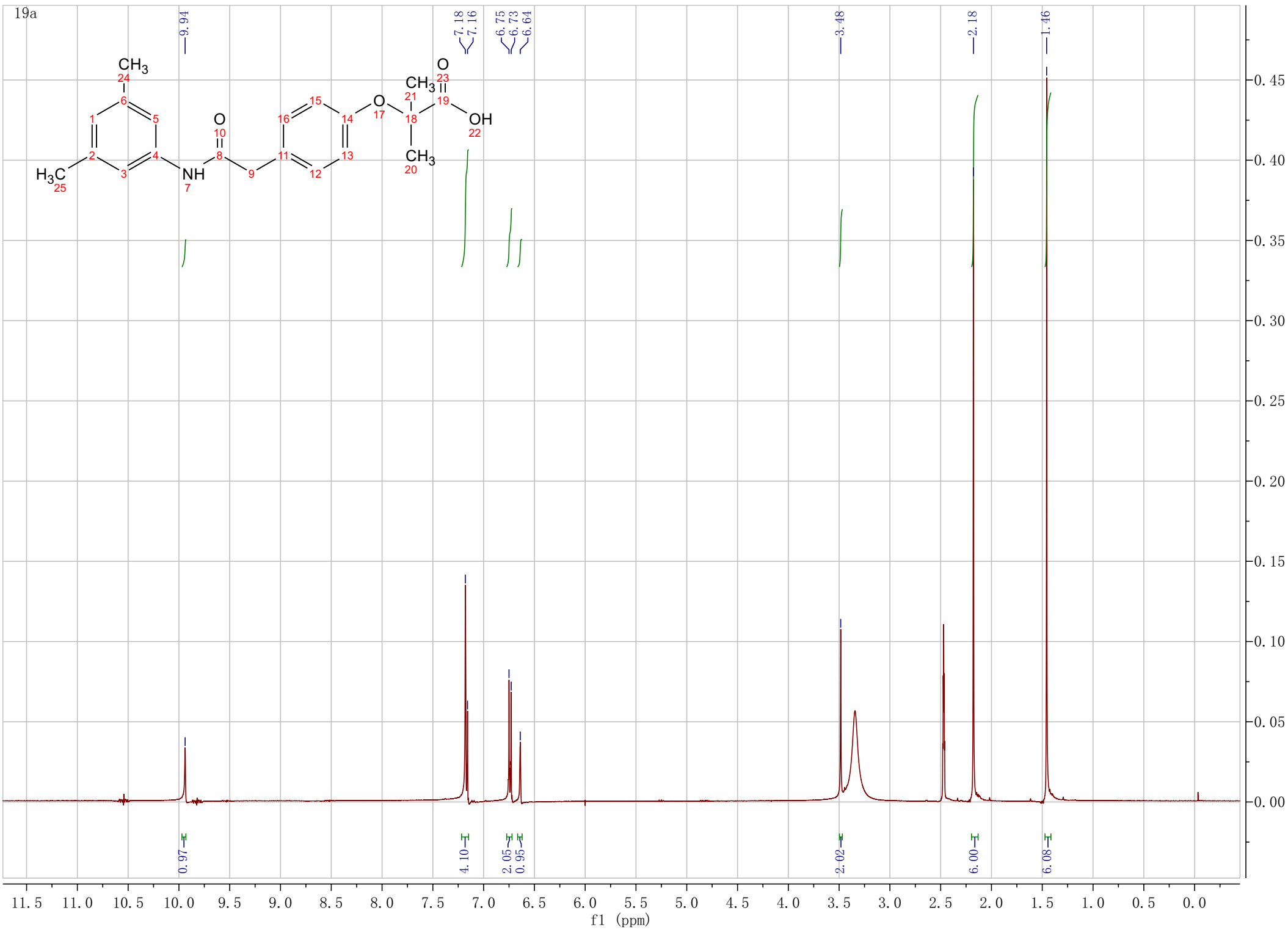

19b

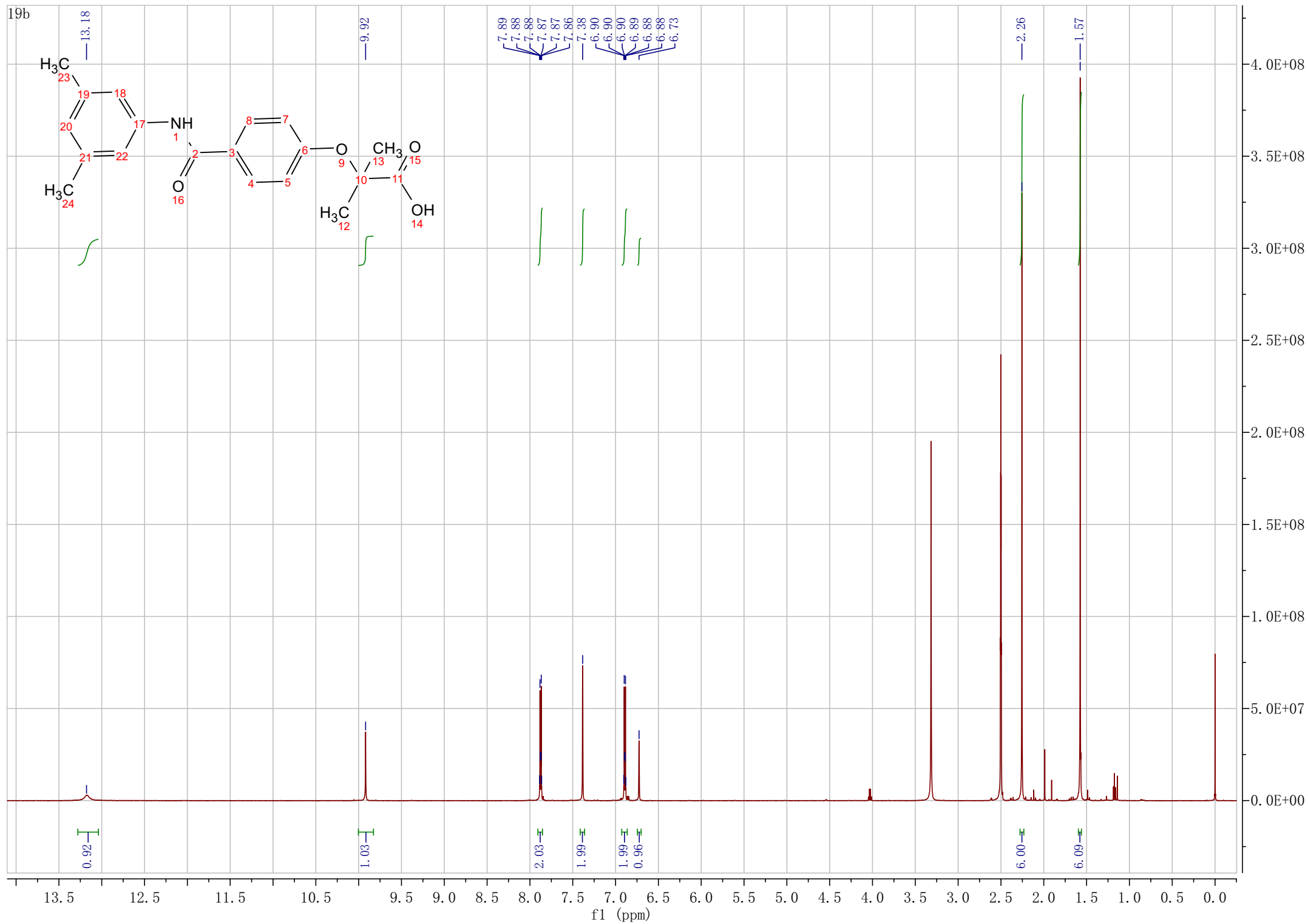

19c

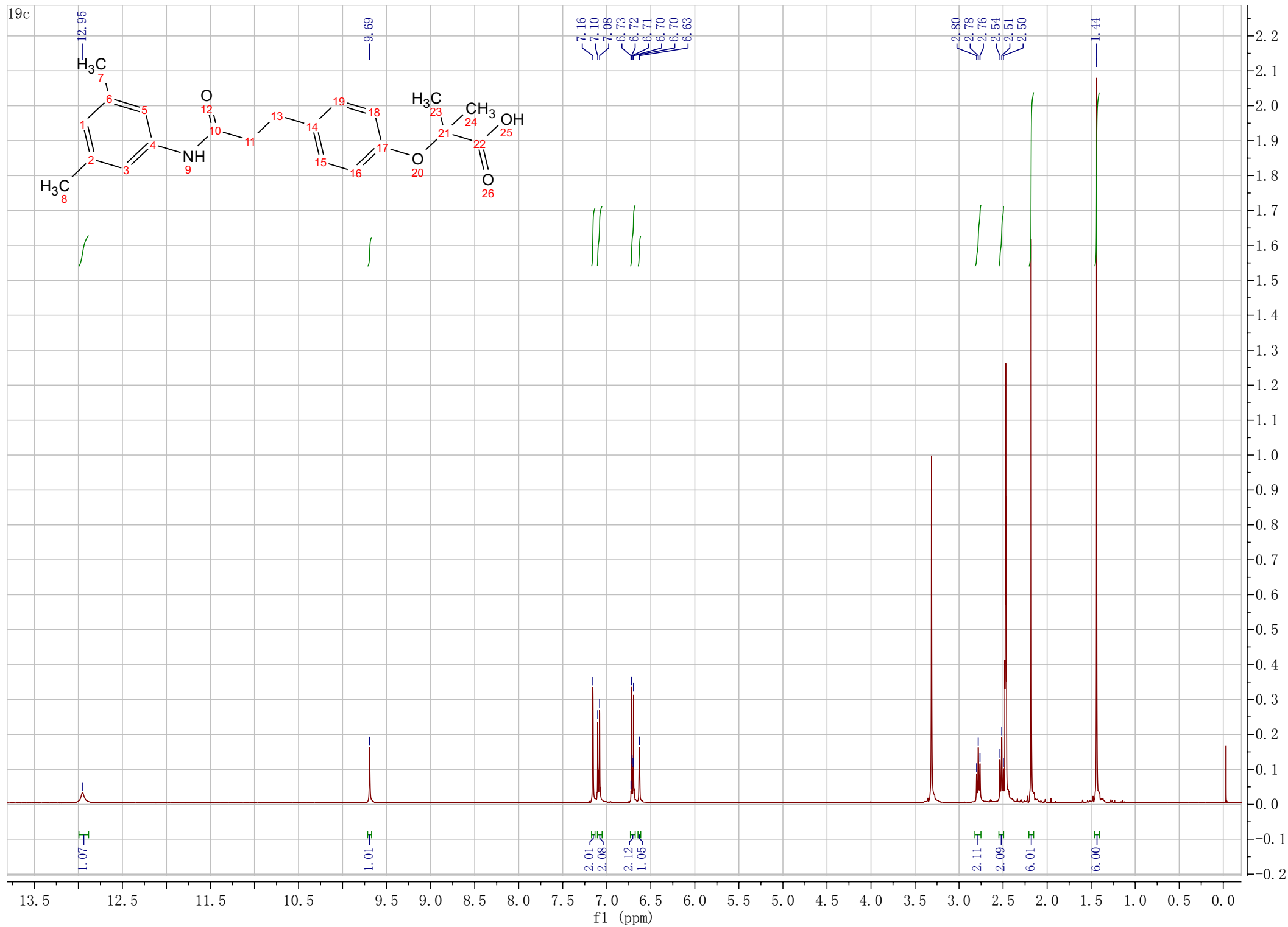

19d

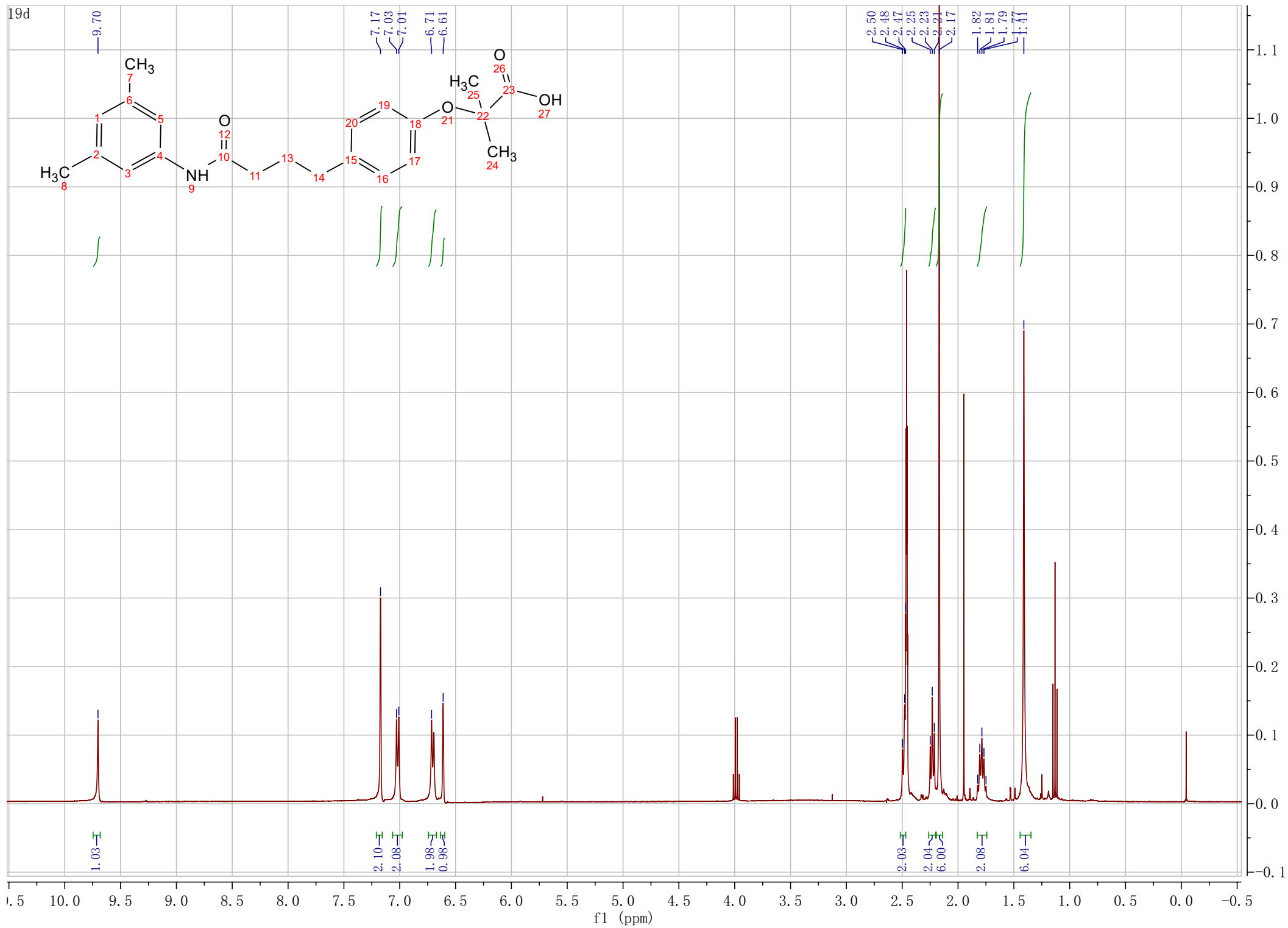

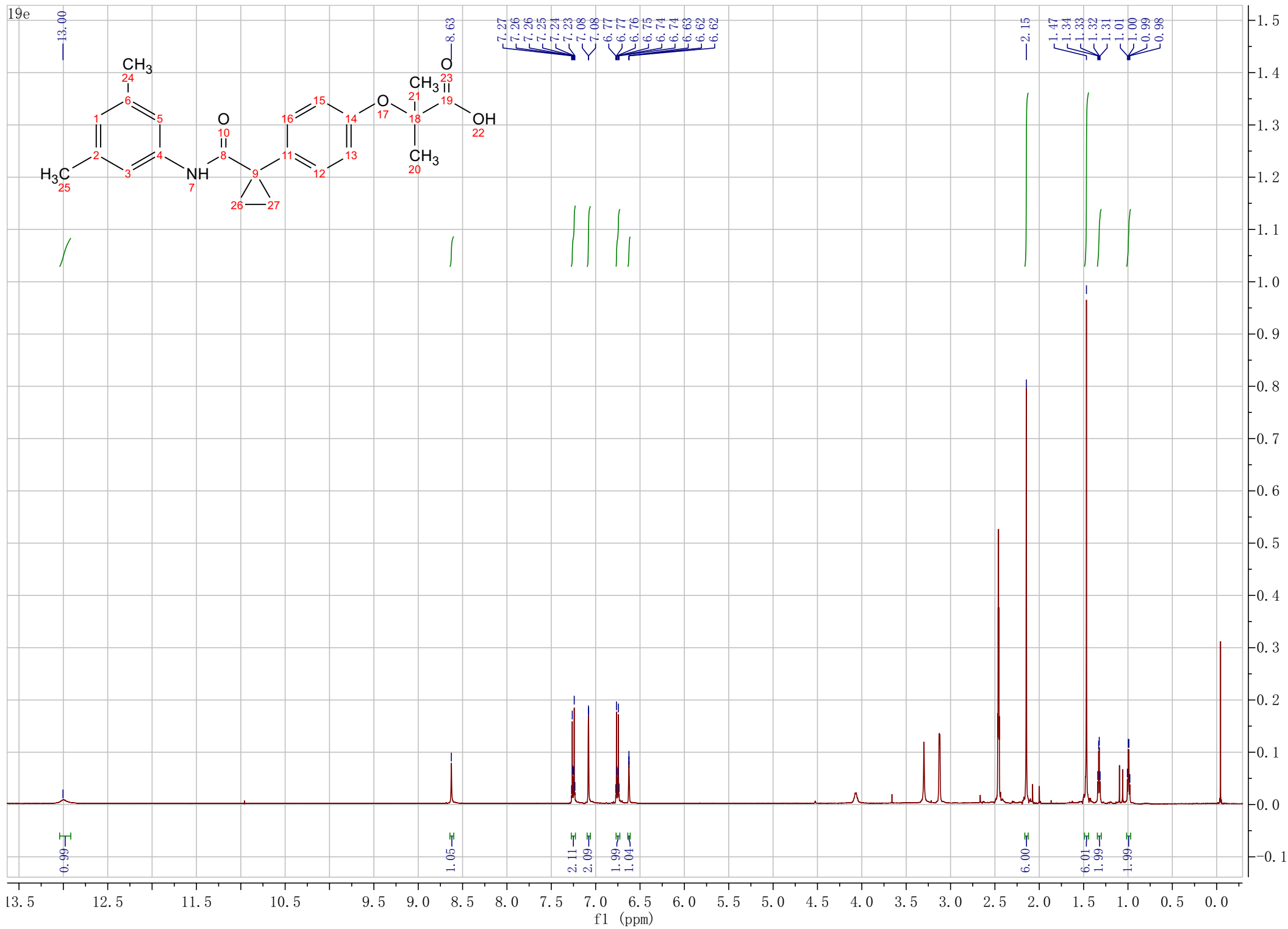

19f

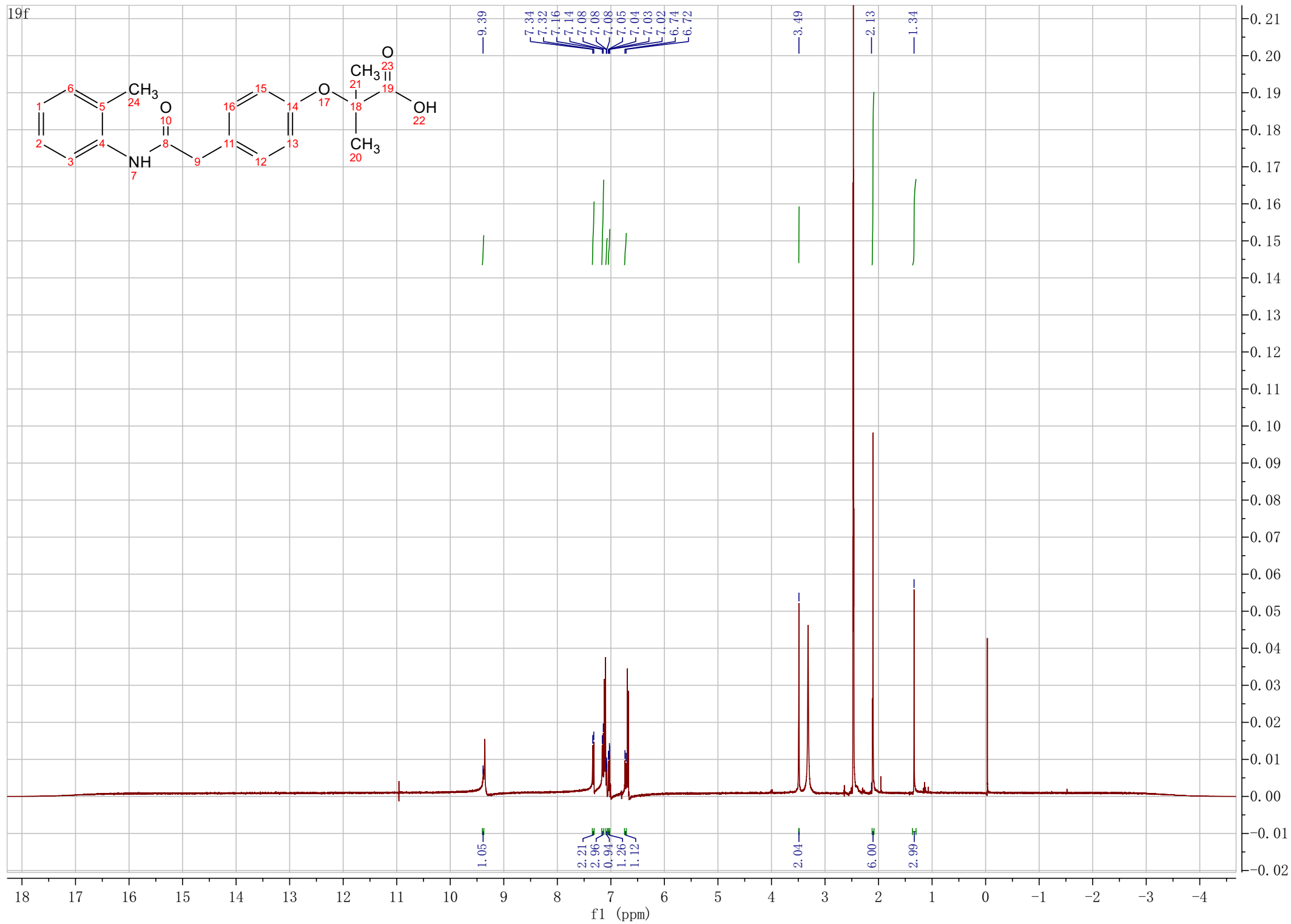

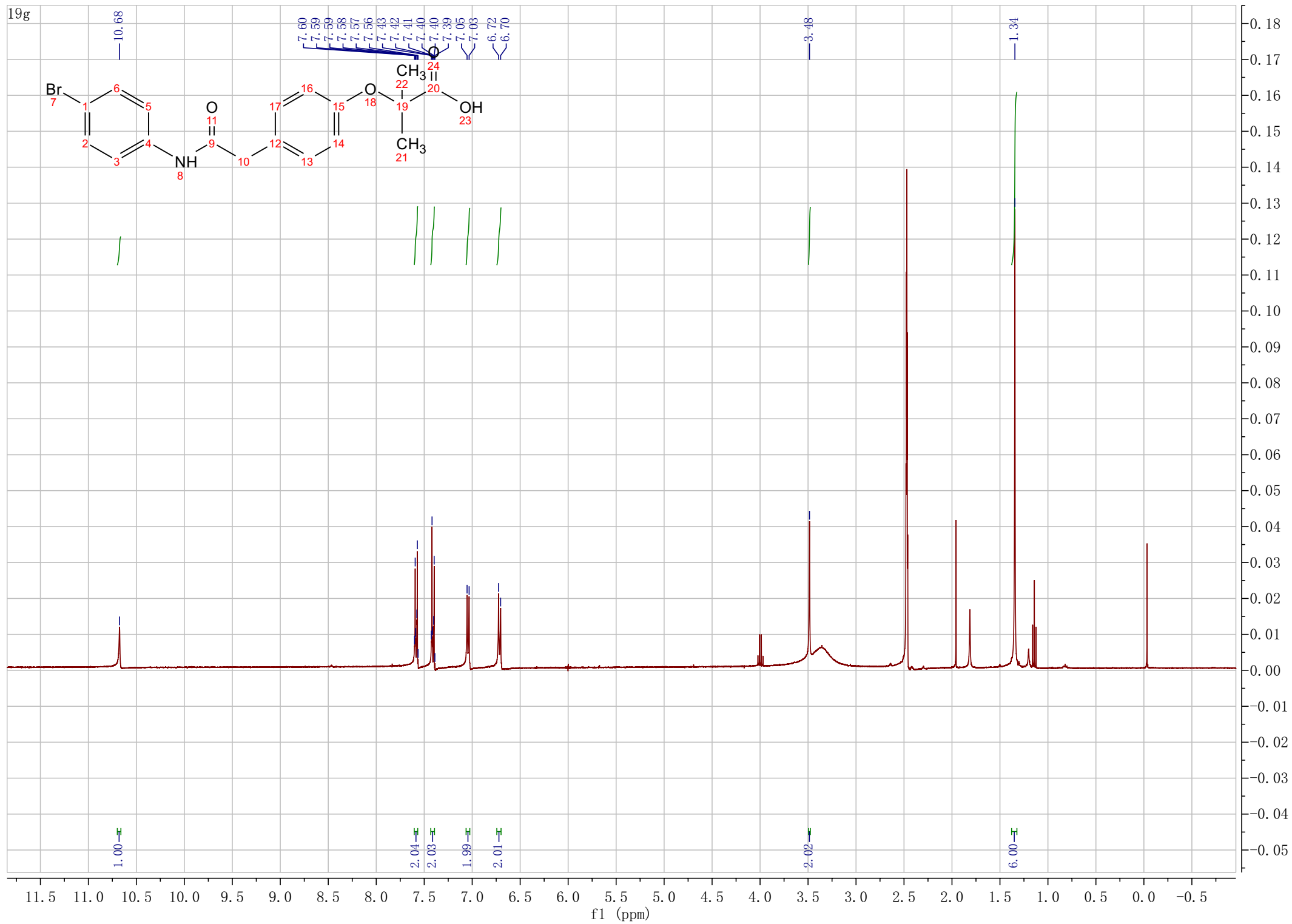

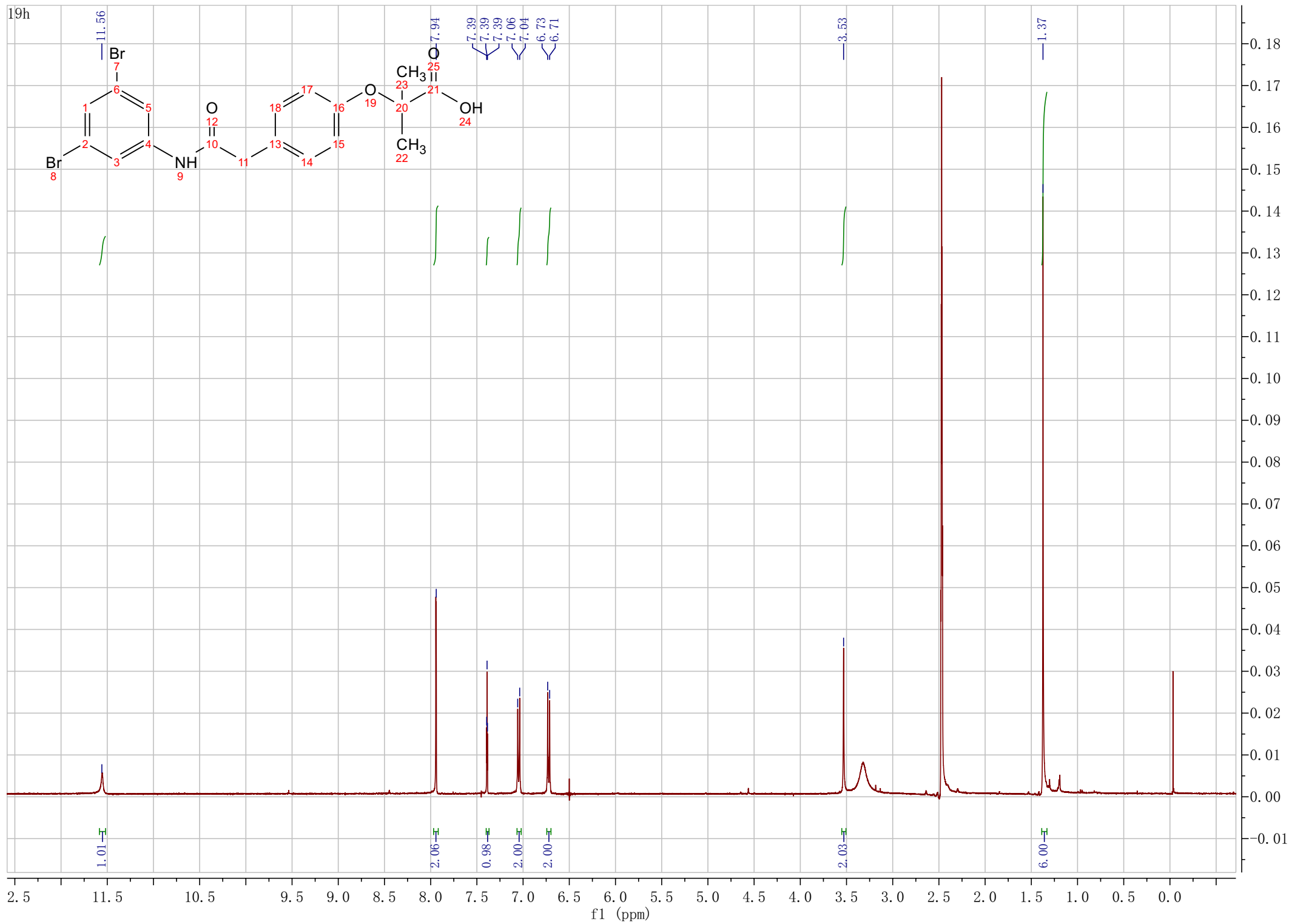

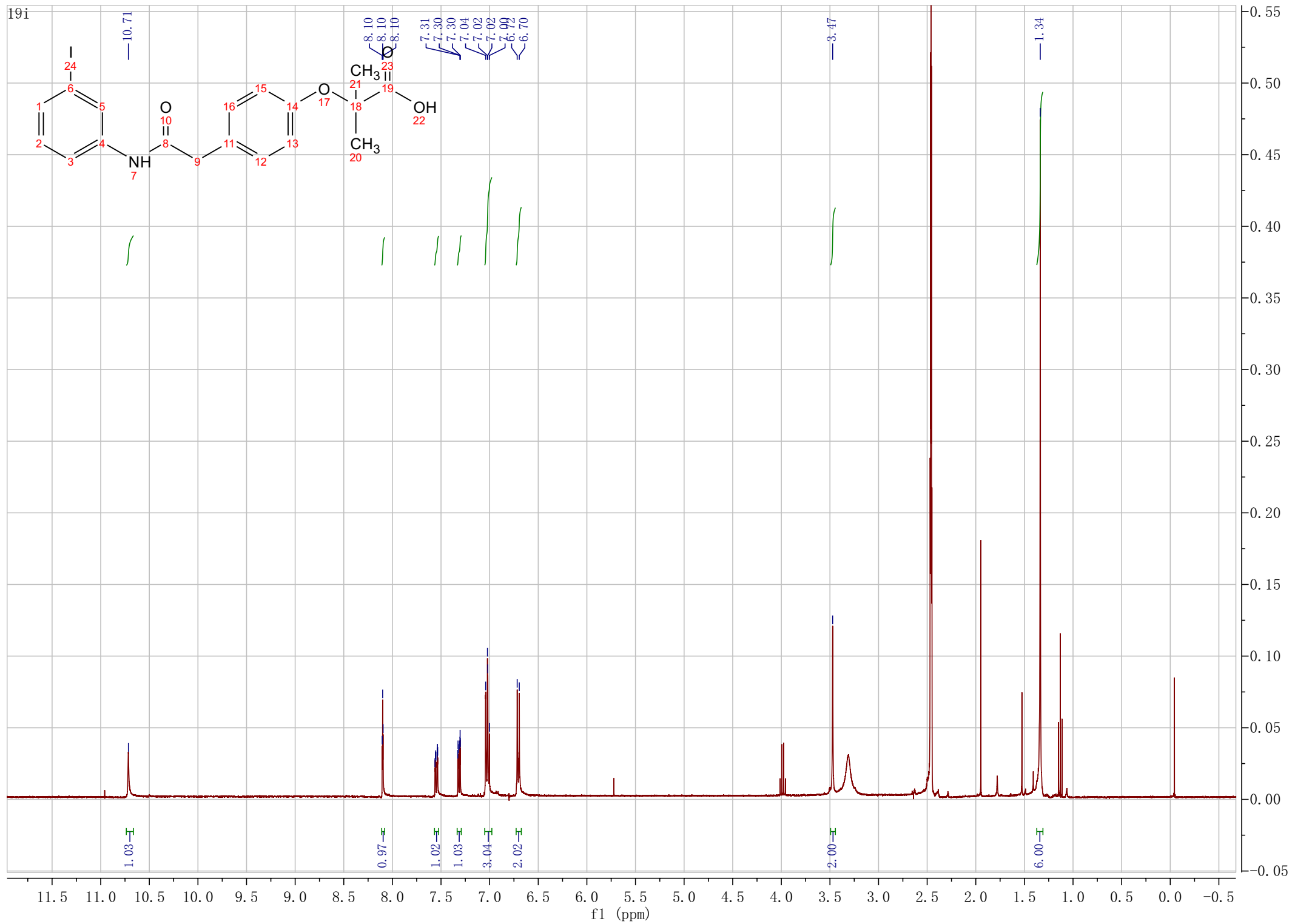

19j

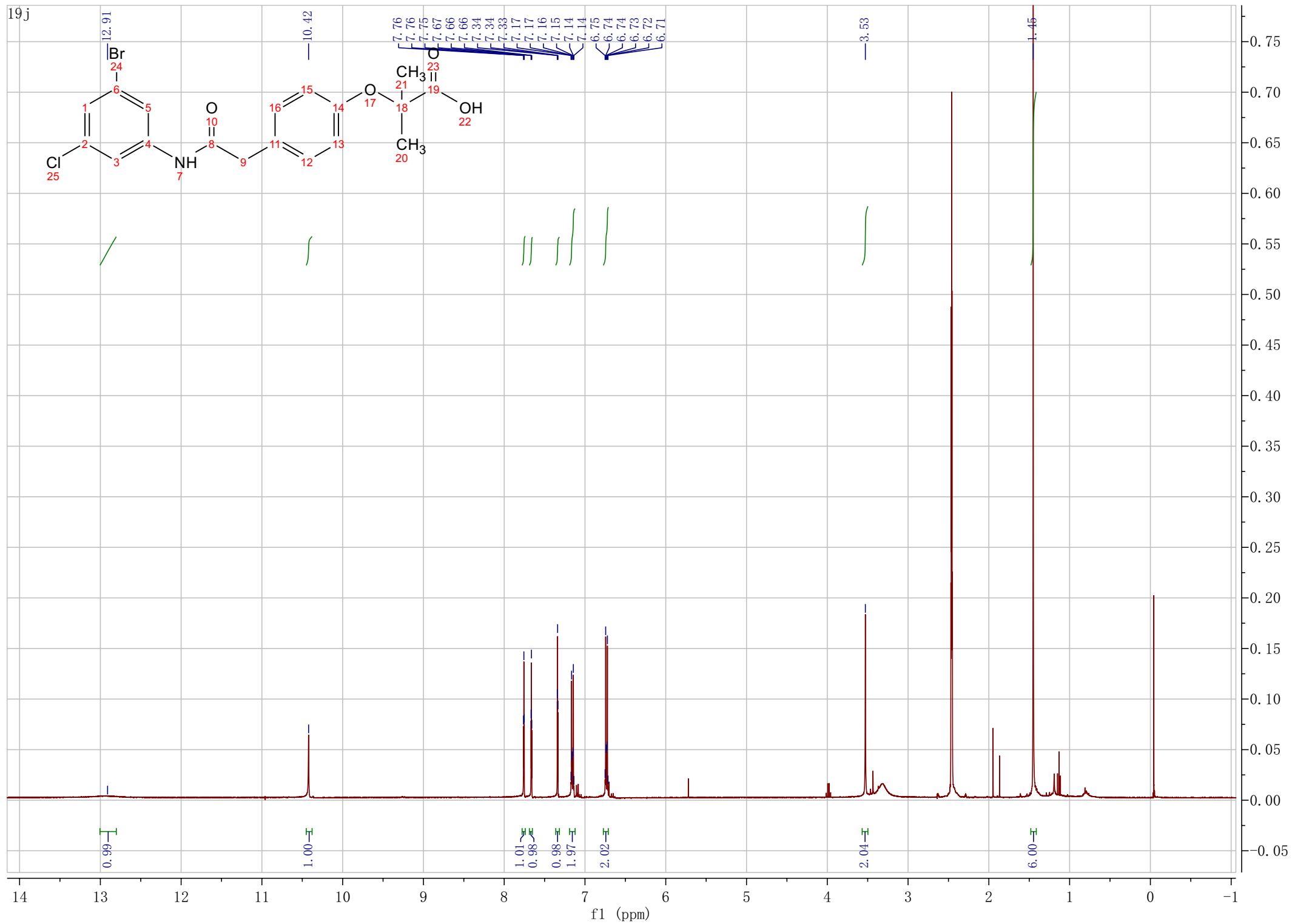

19k

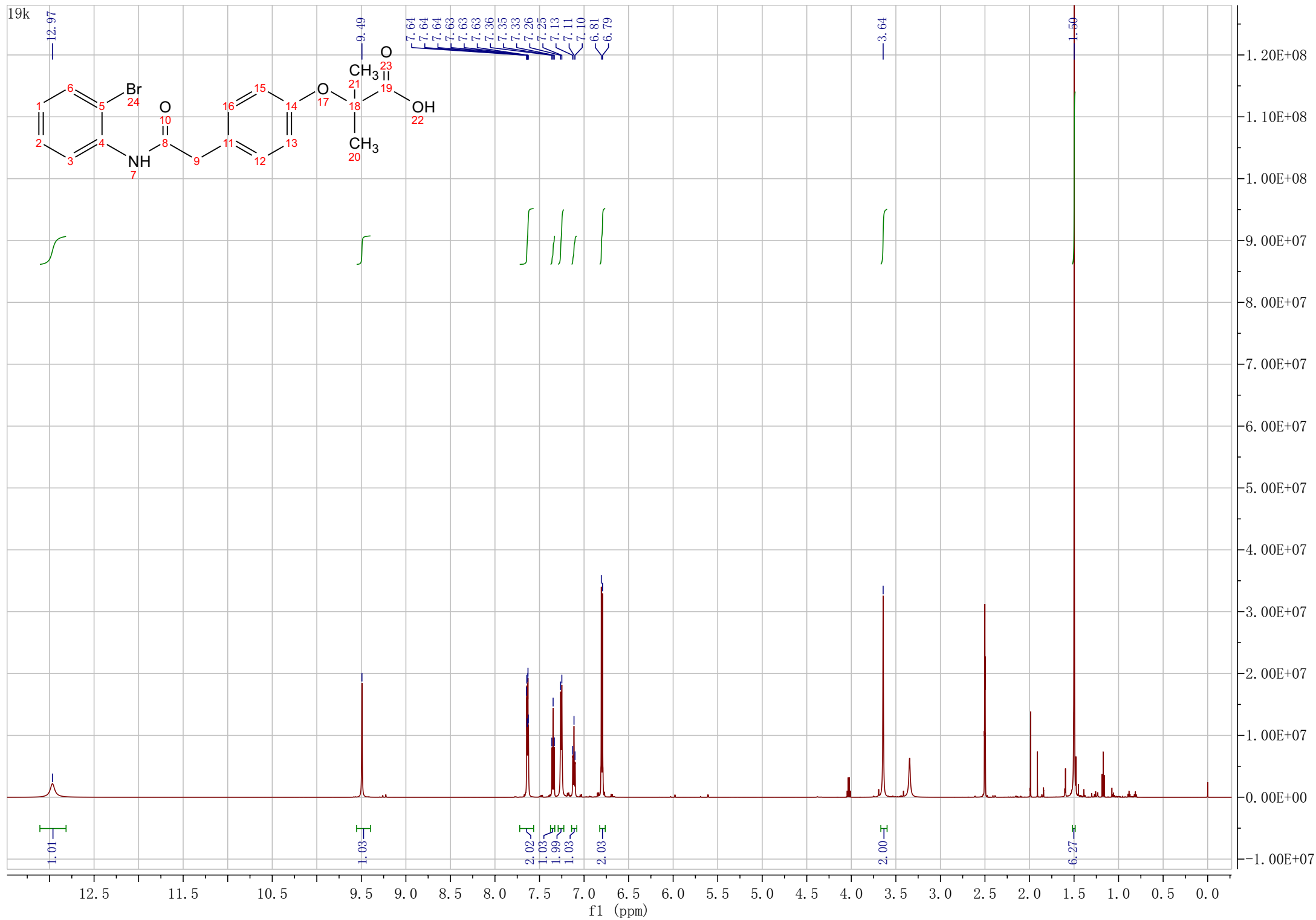

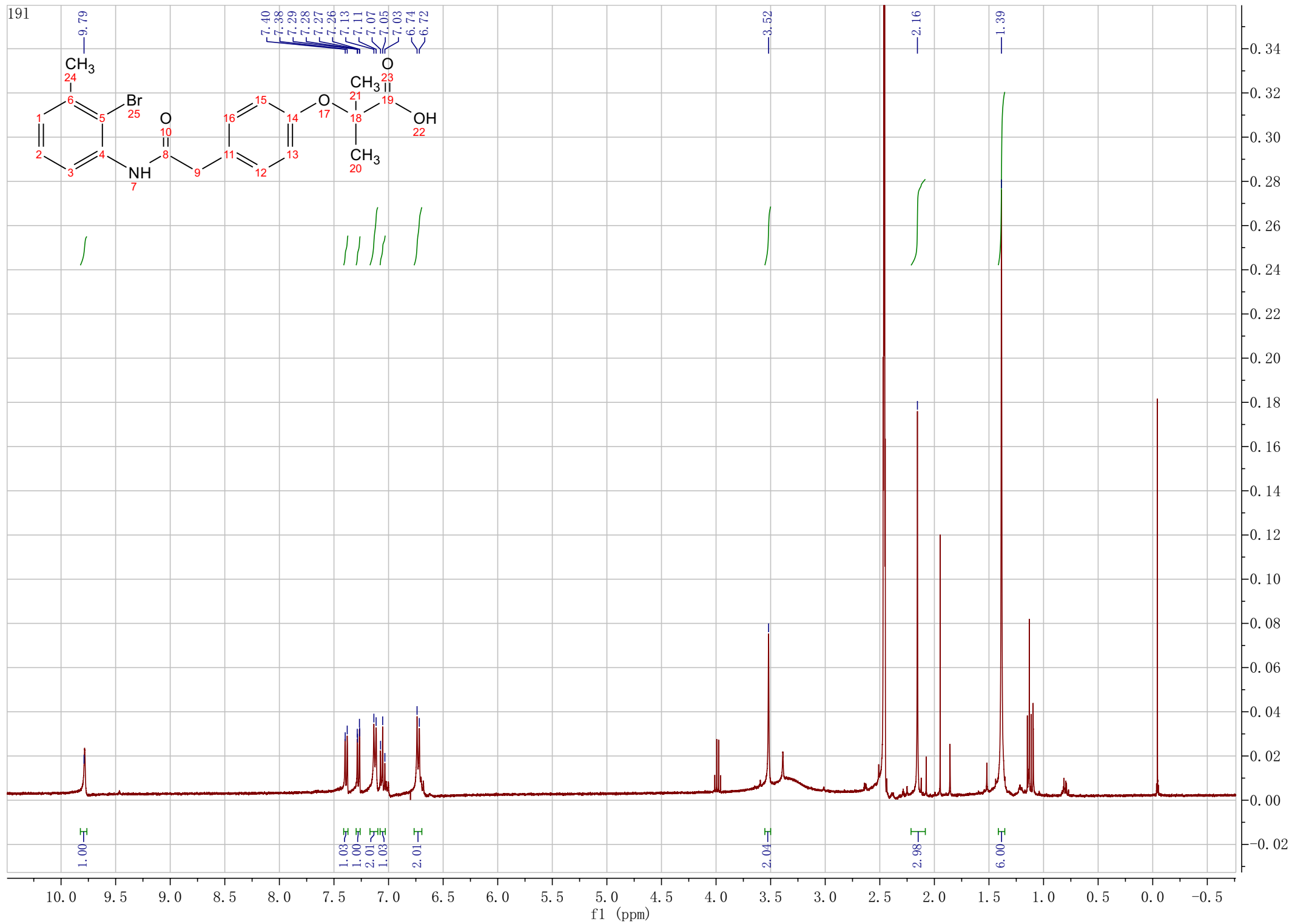

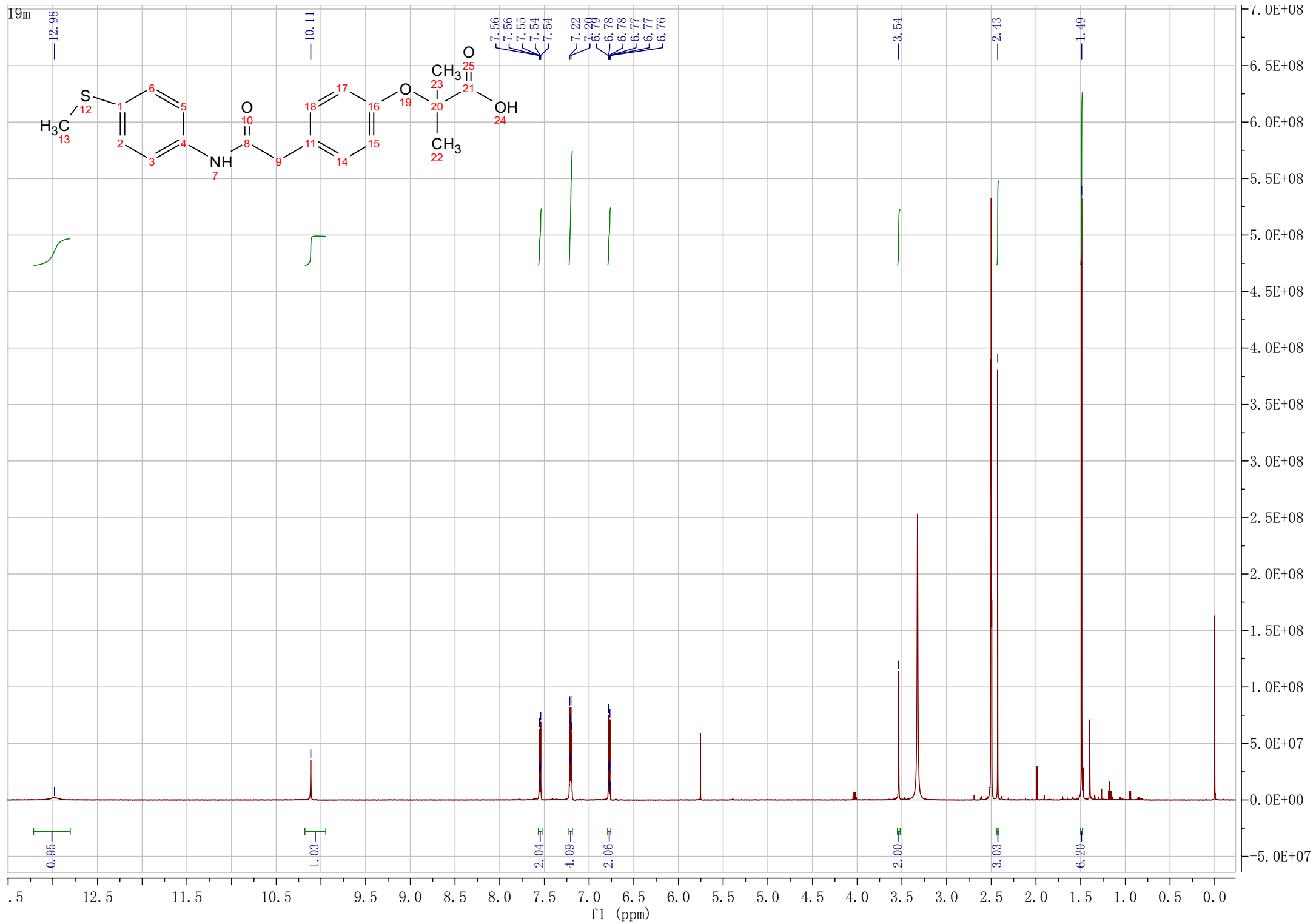

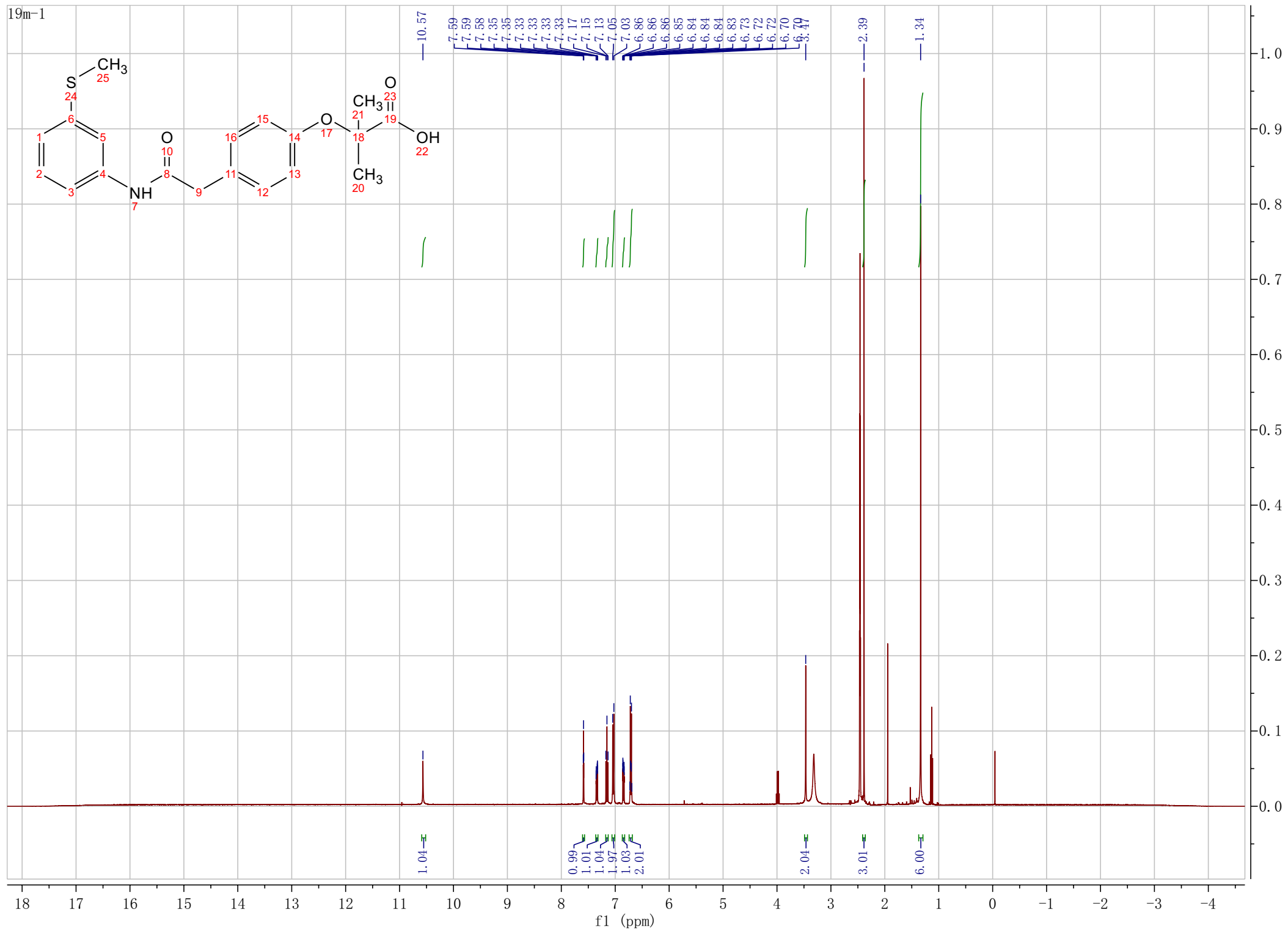

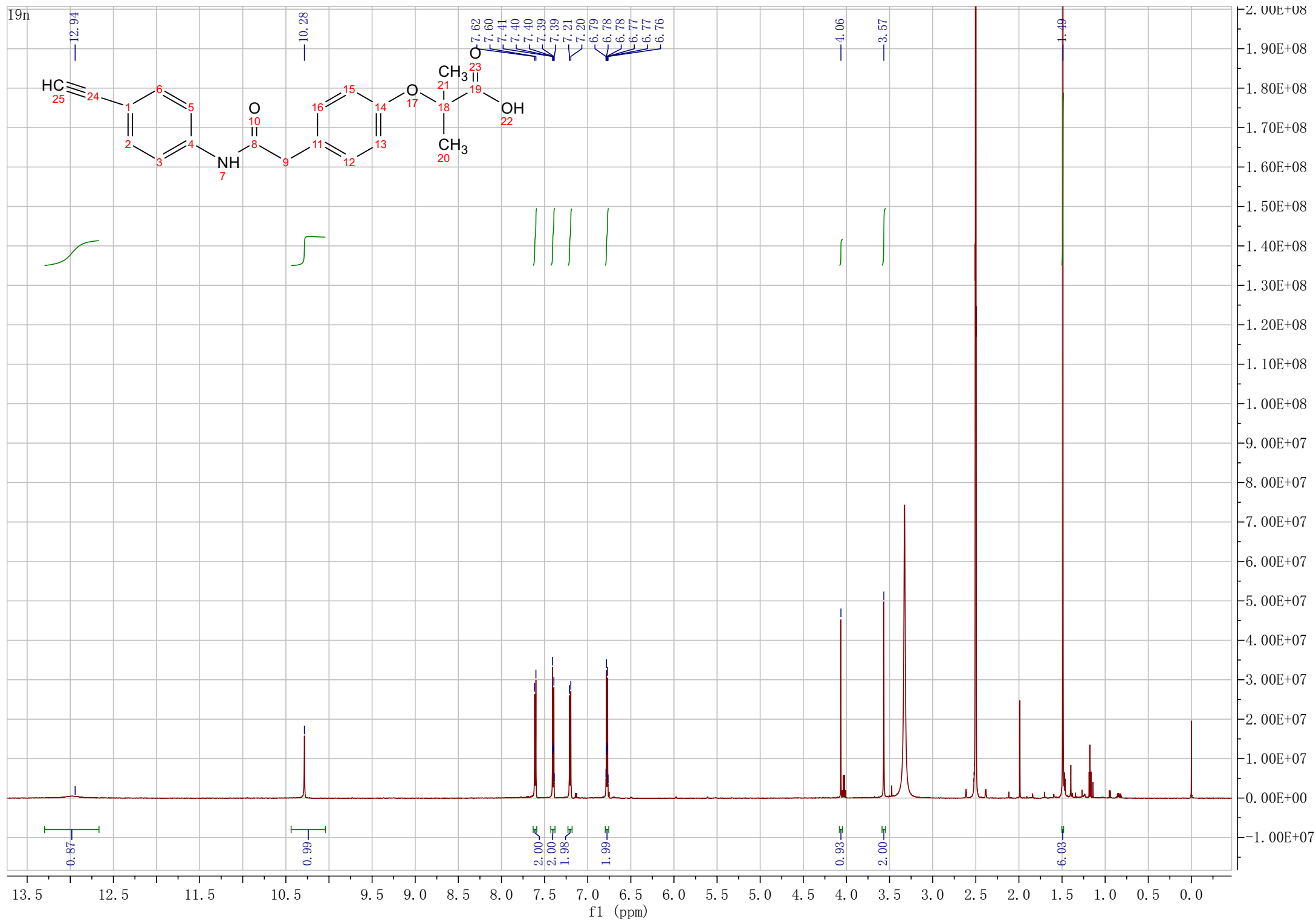

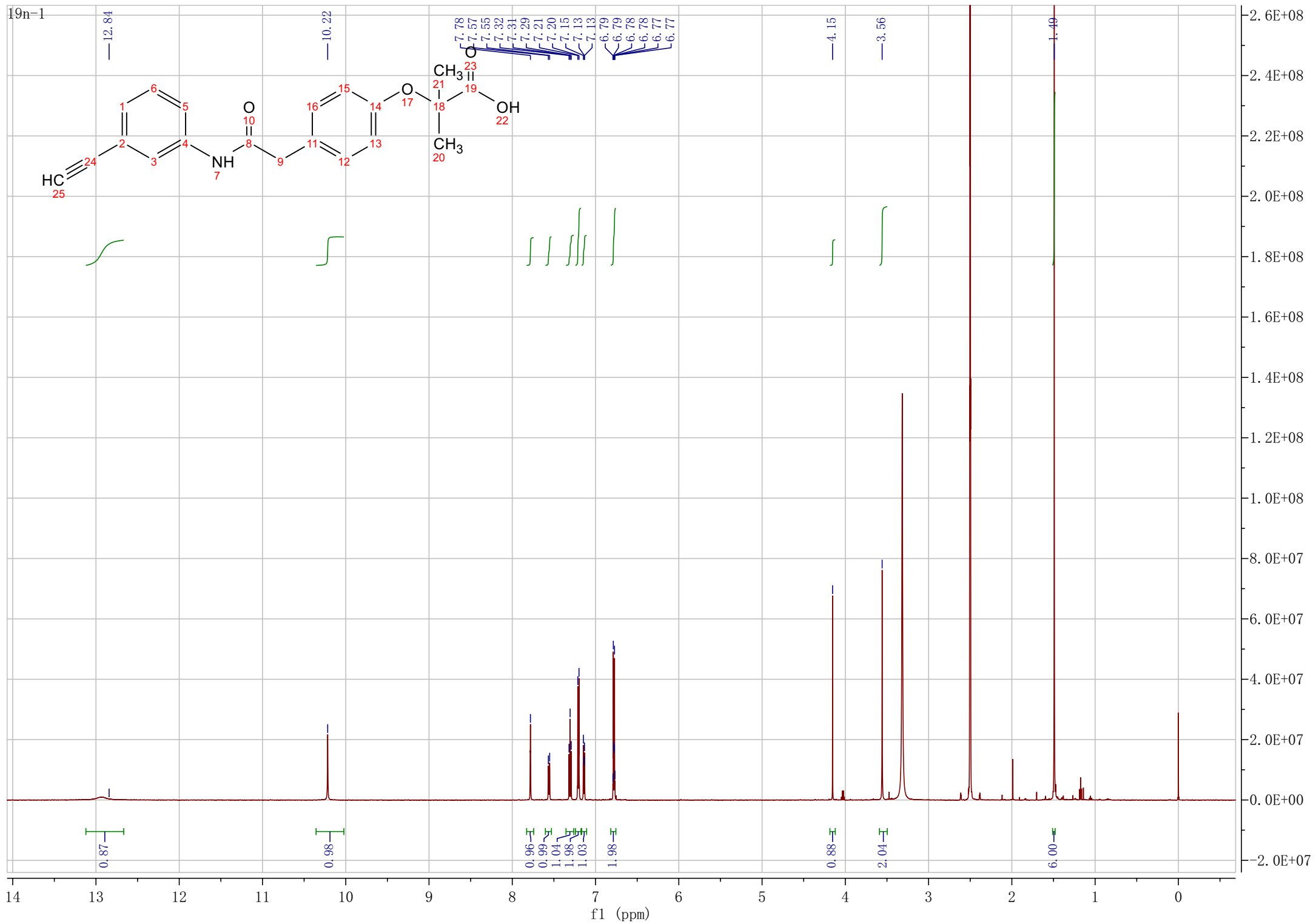

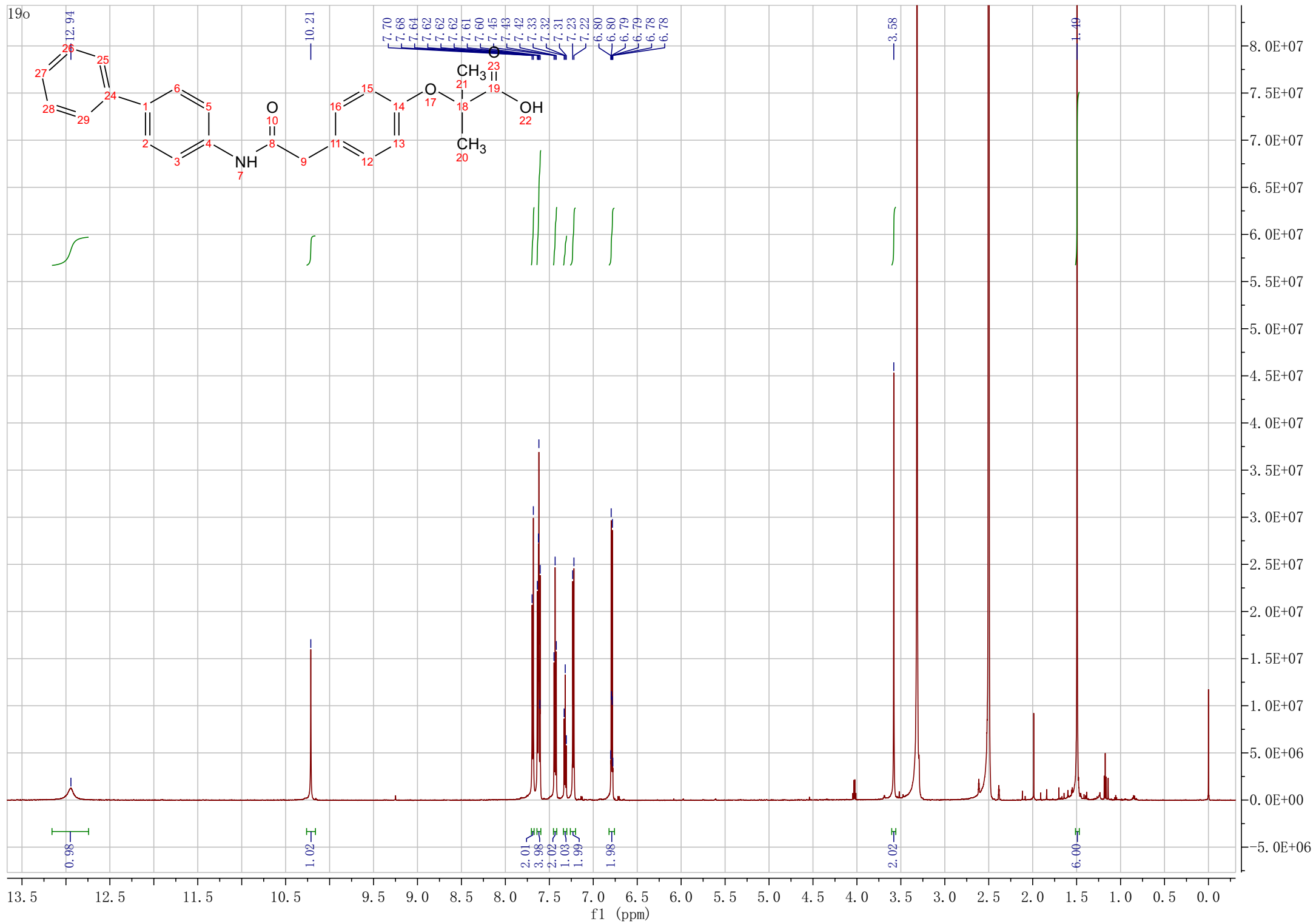

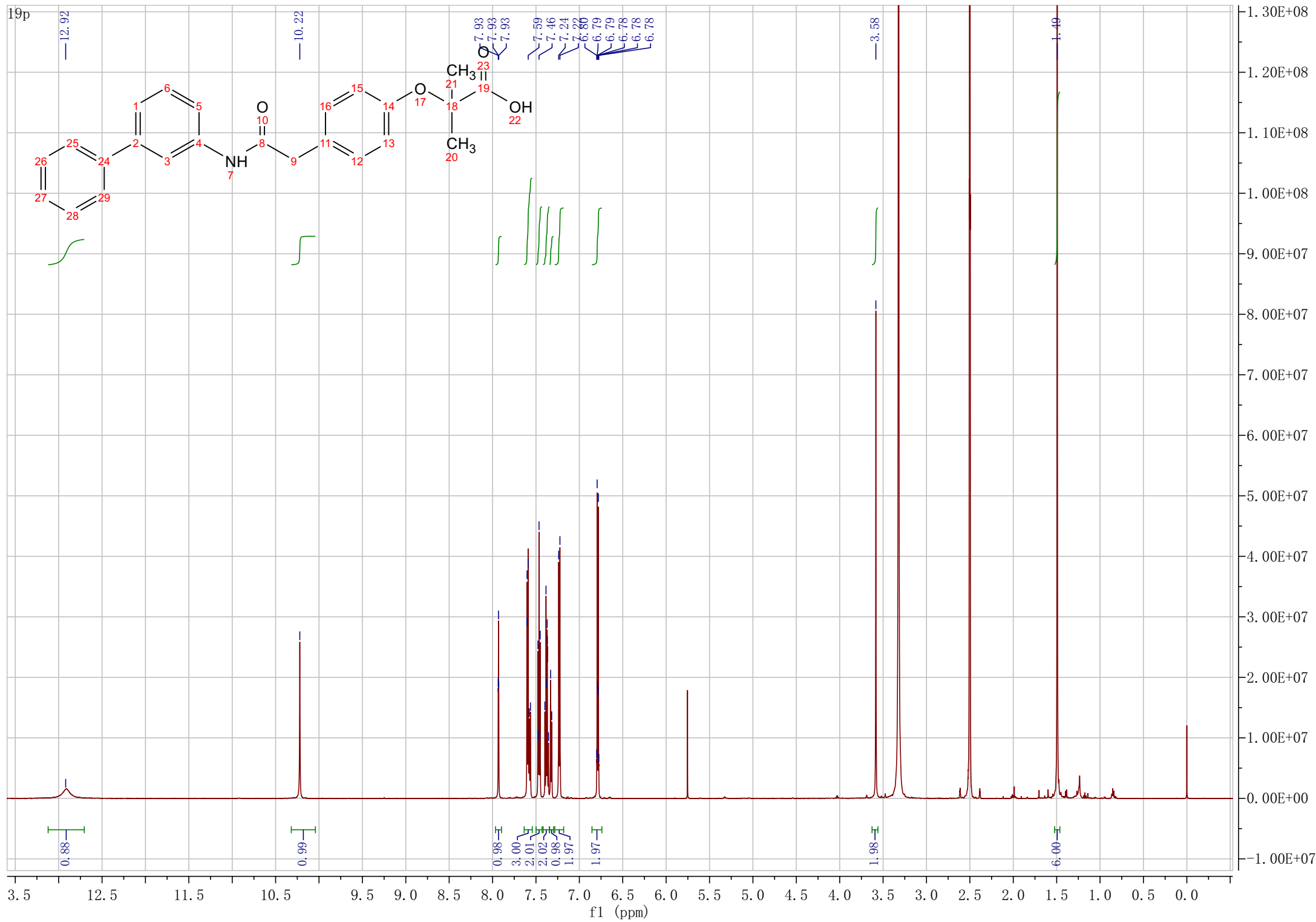

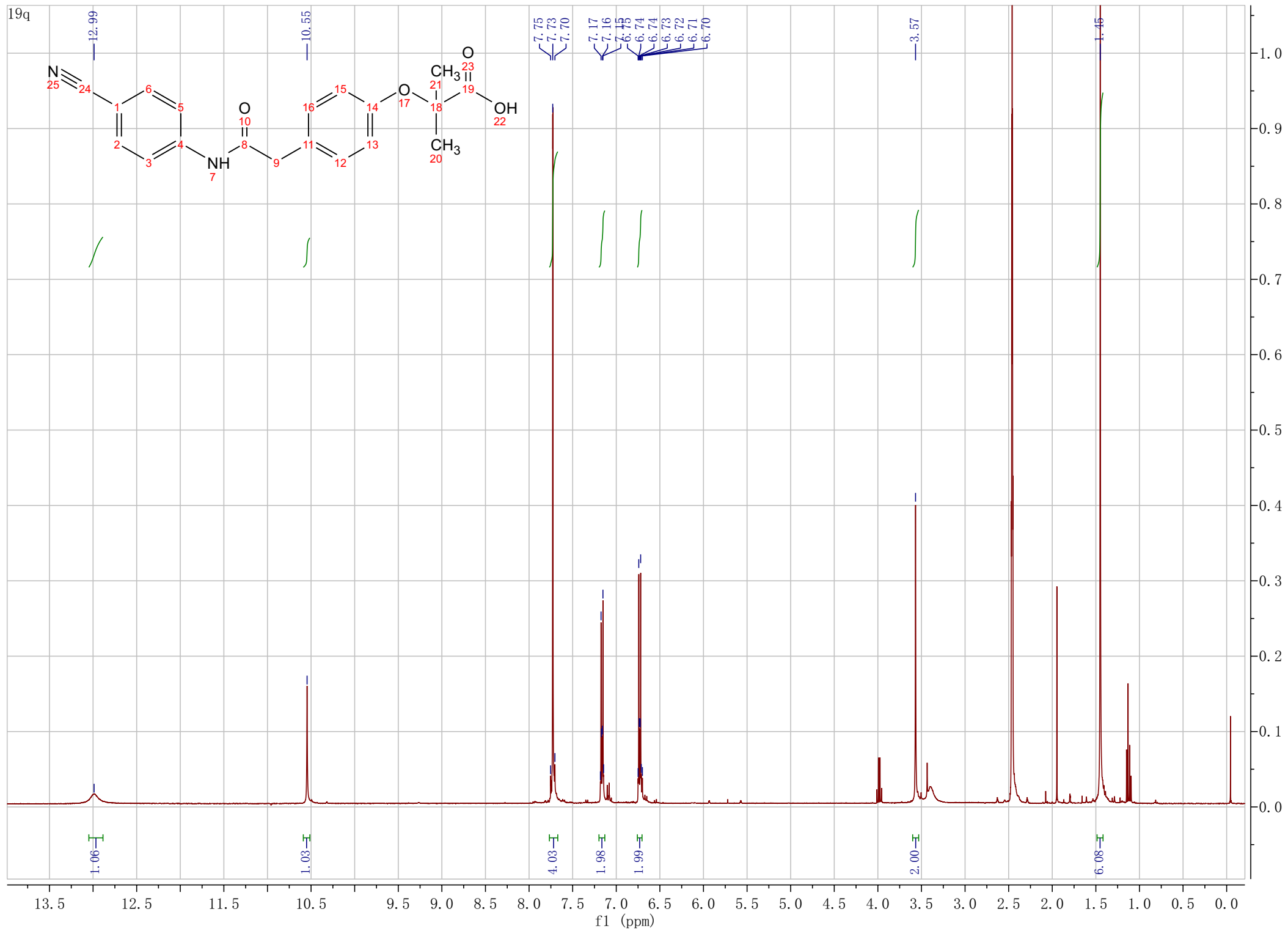

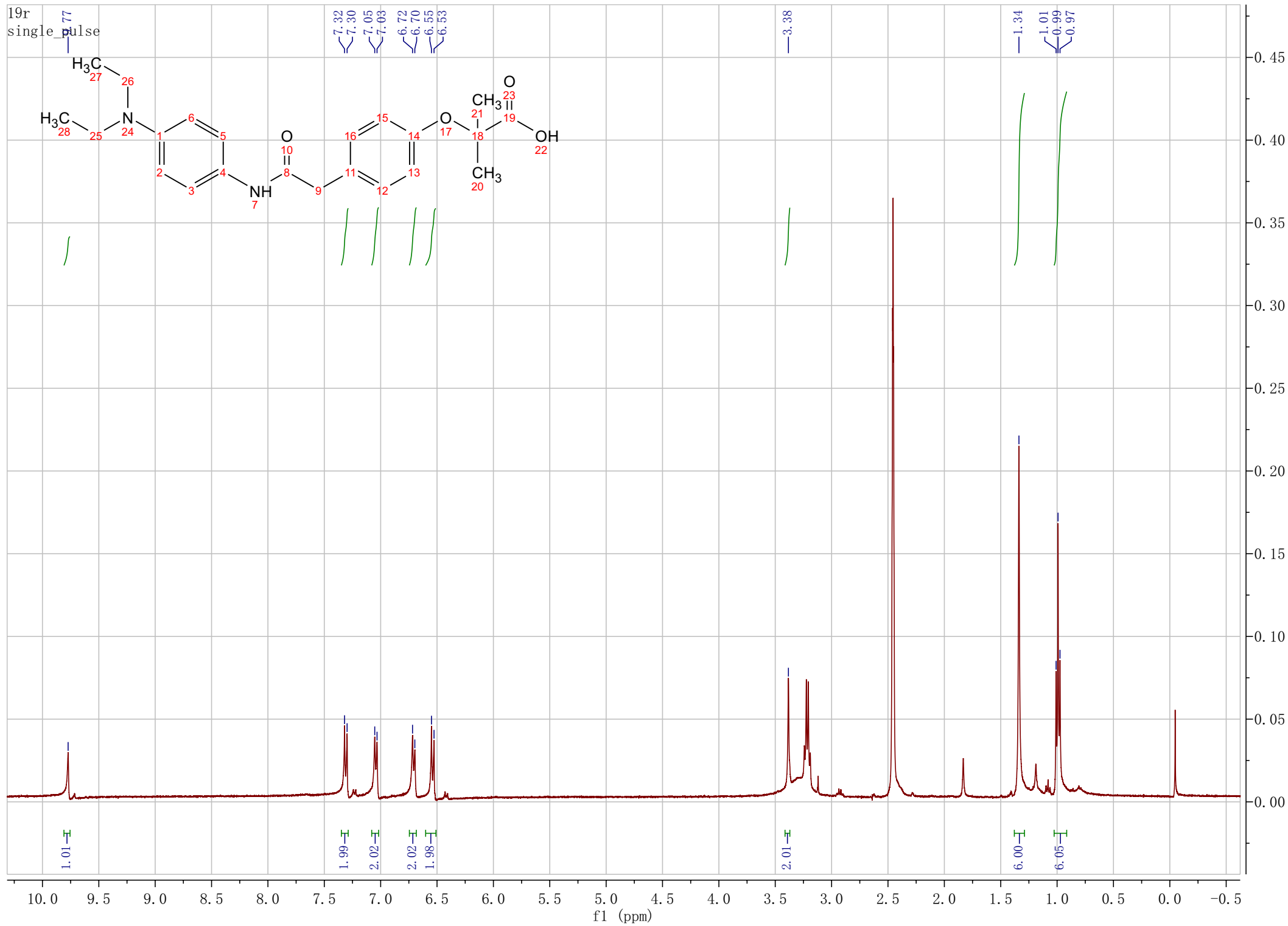

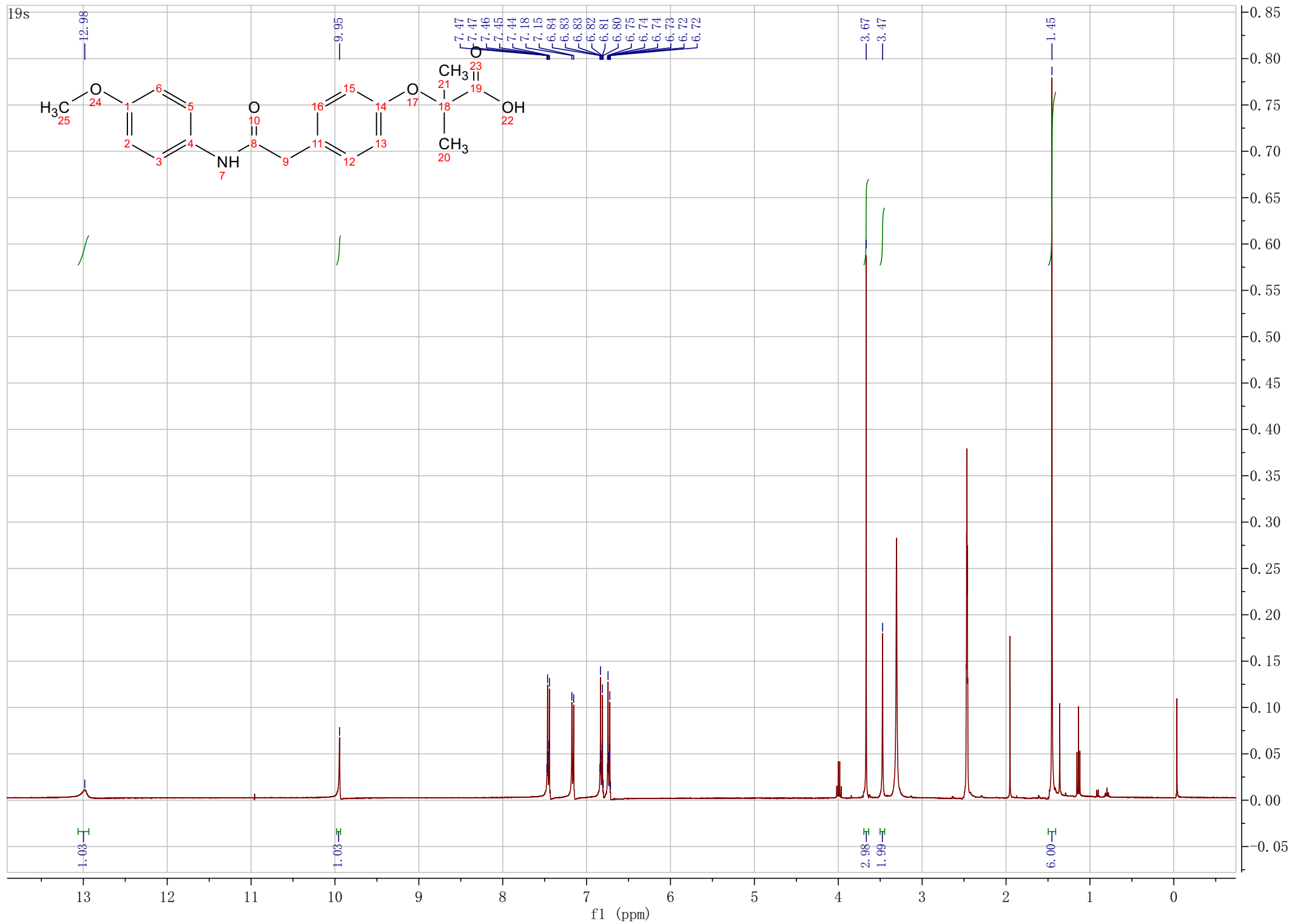

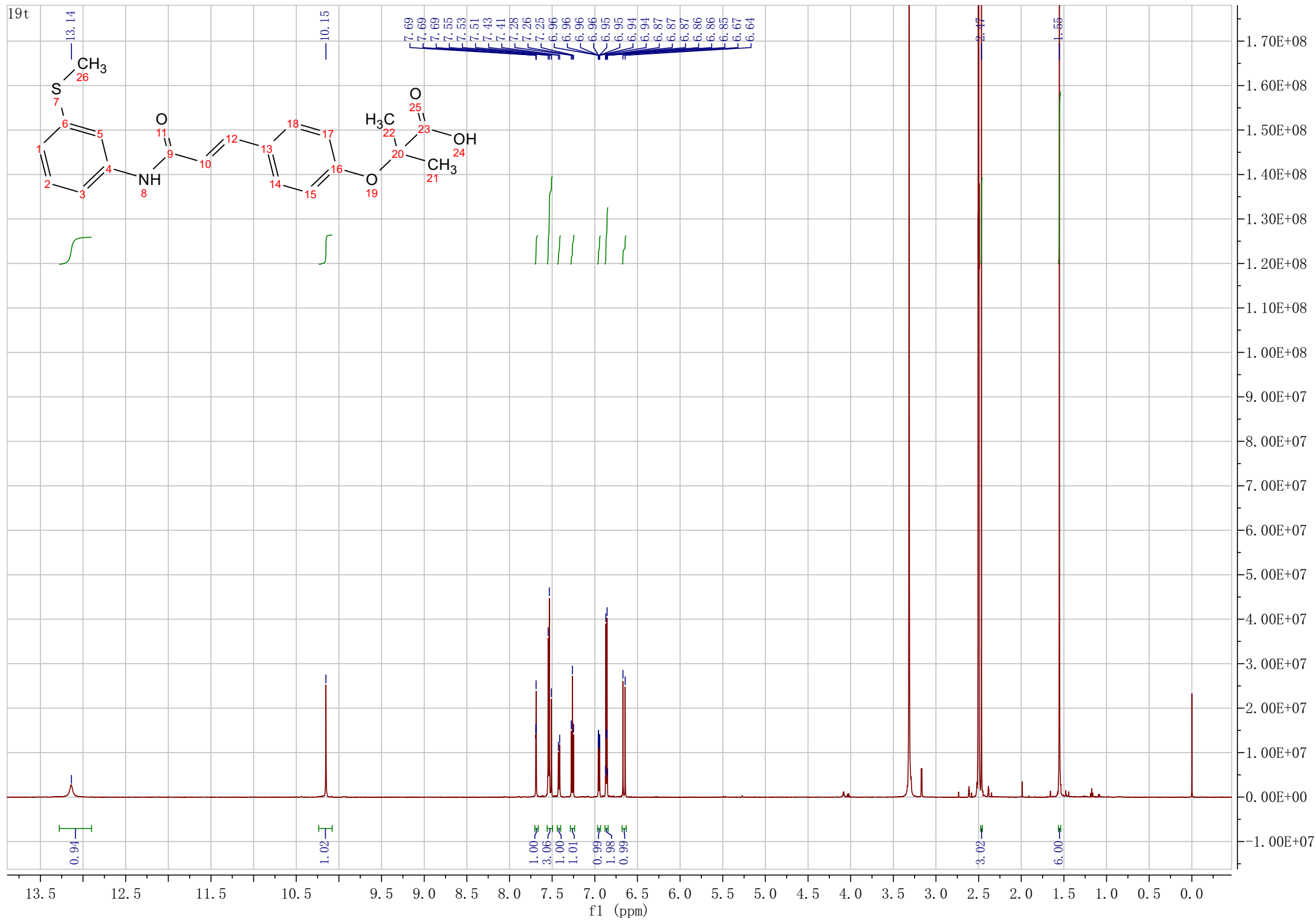

19u

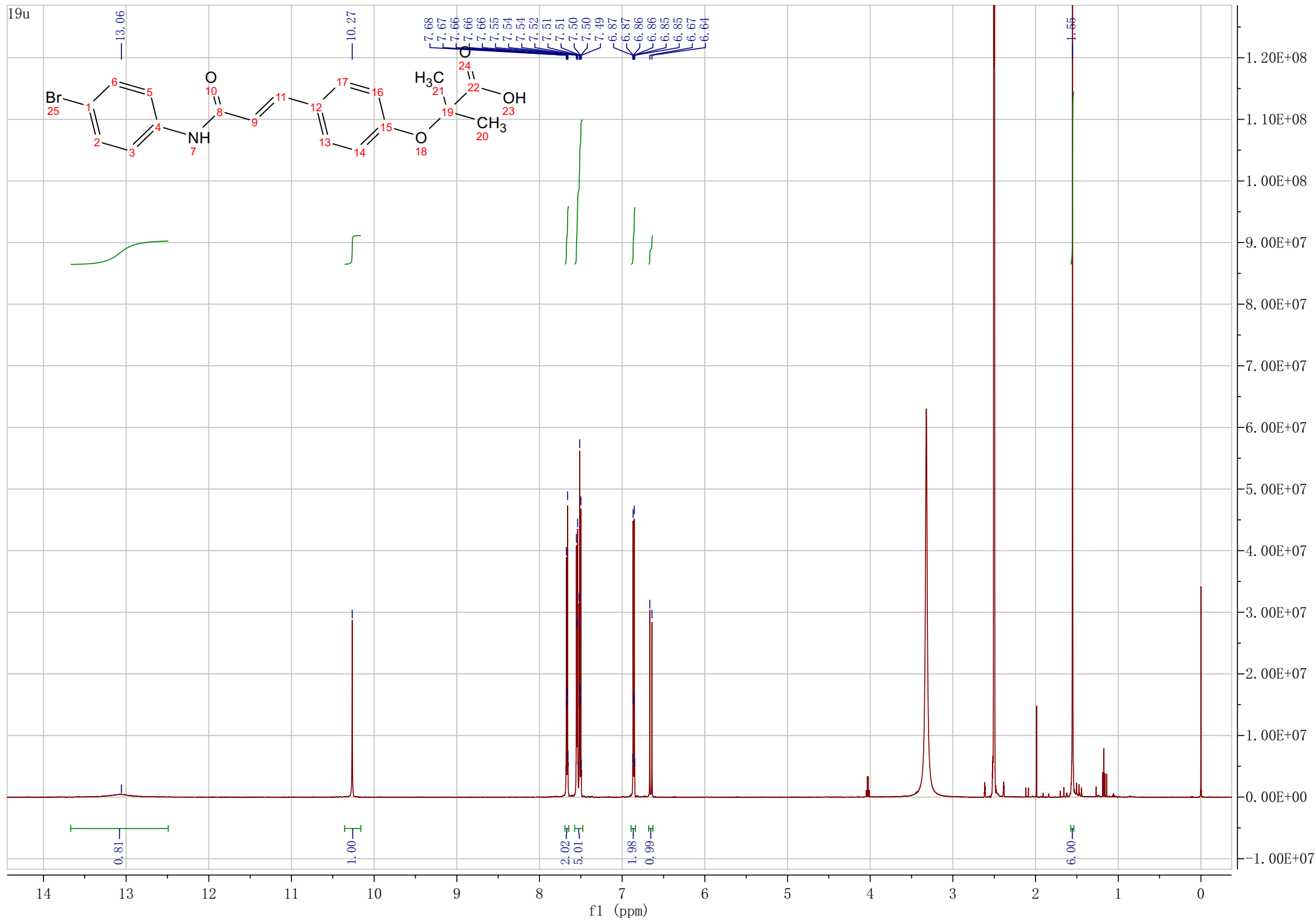

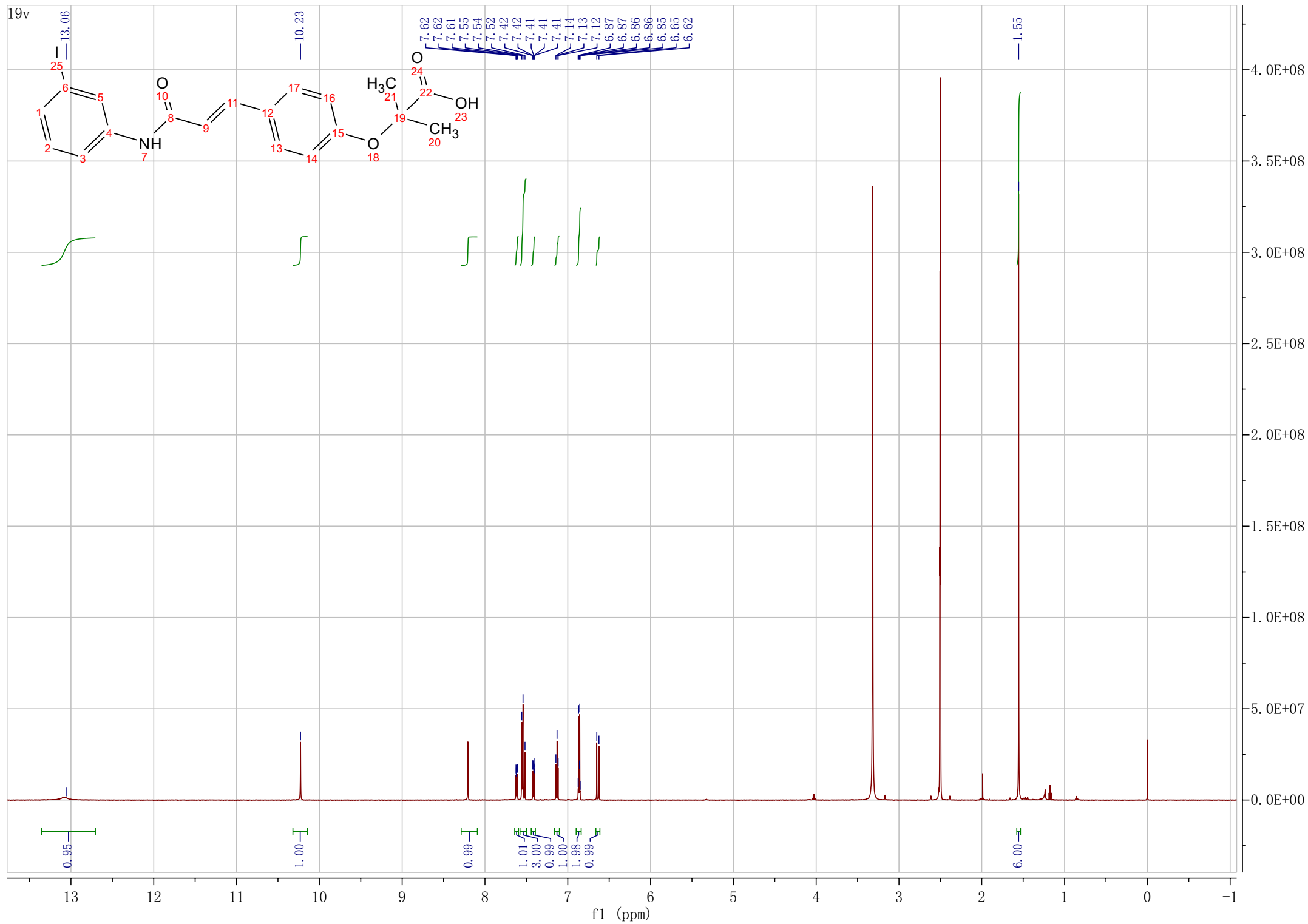

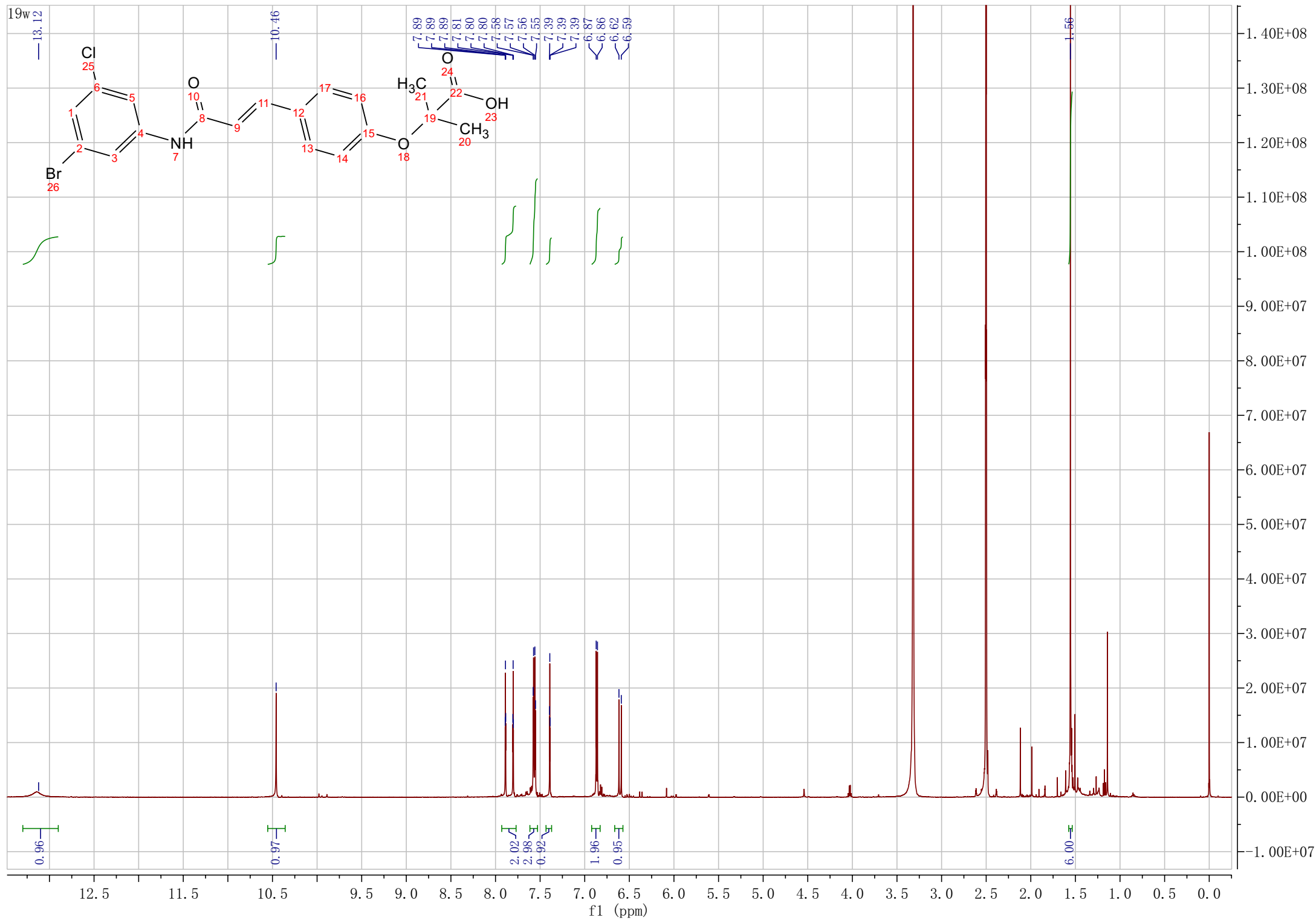

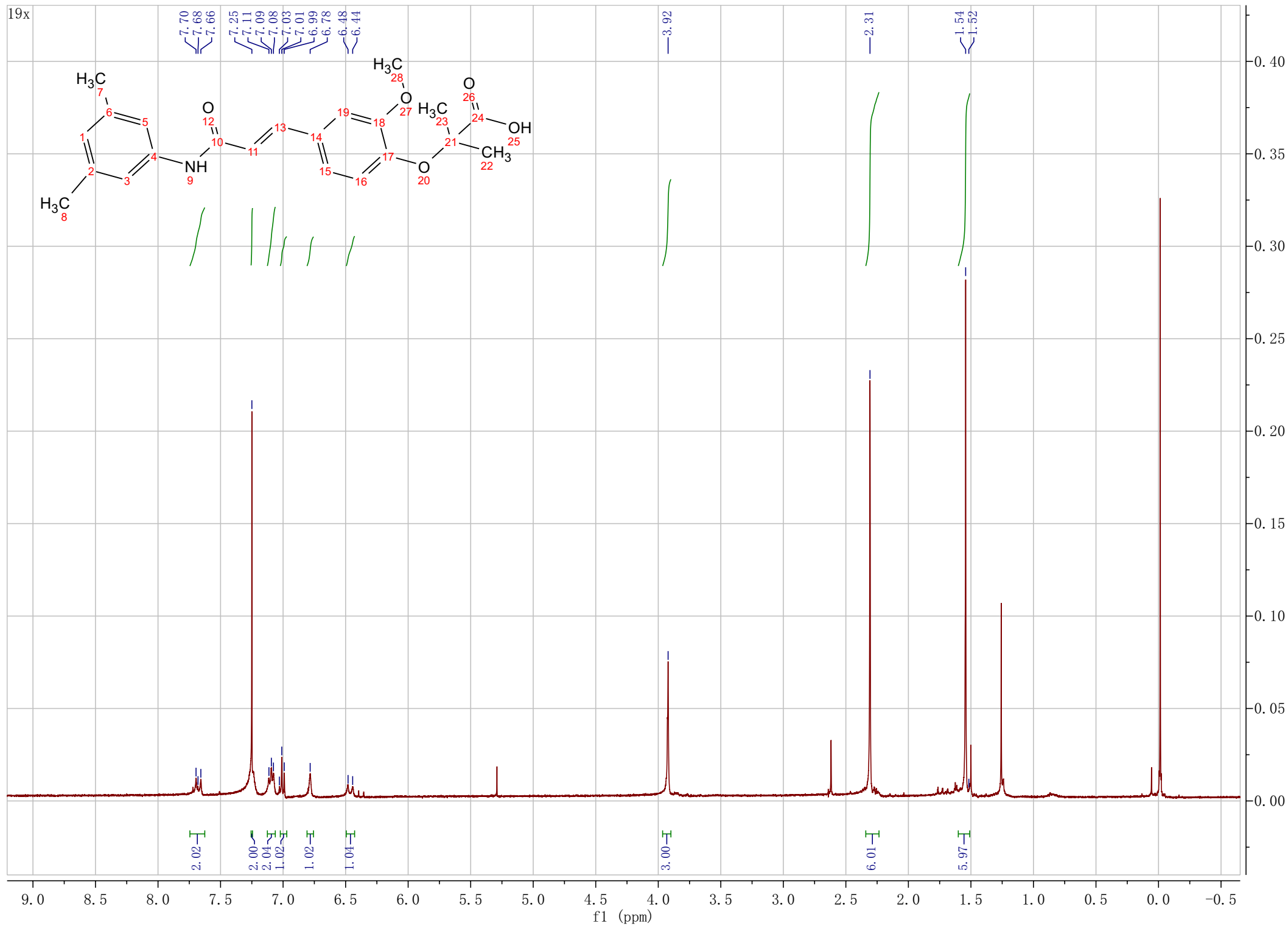

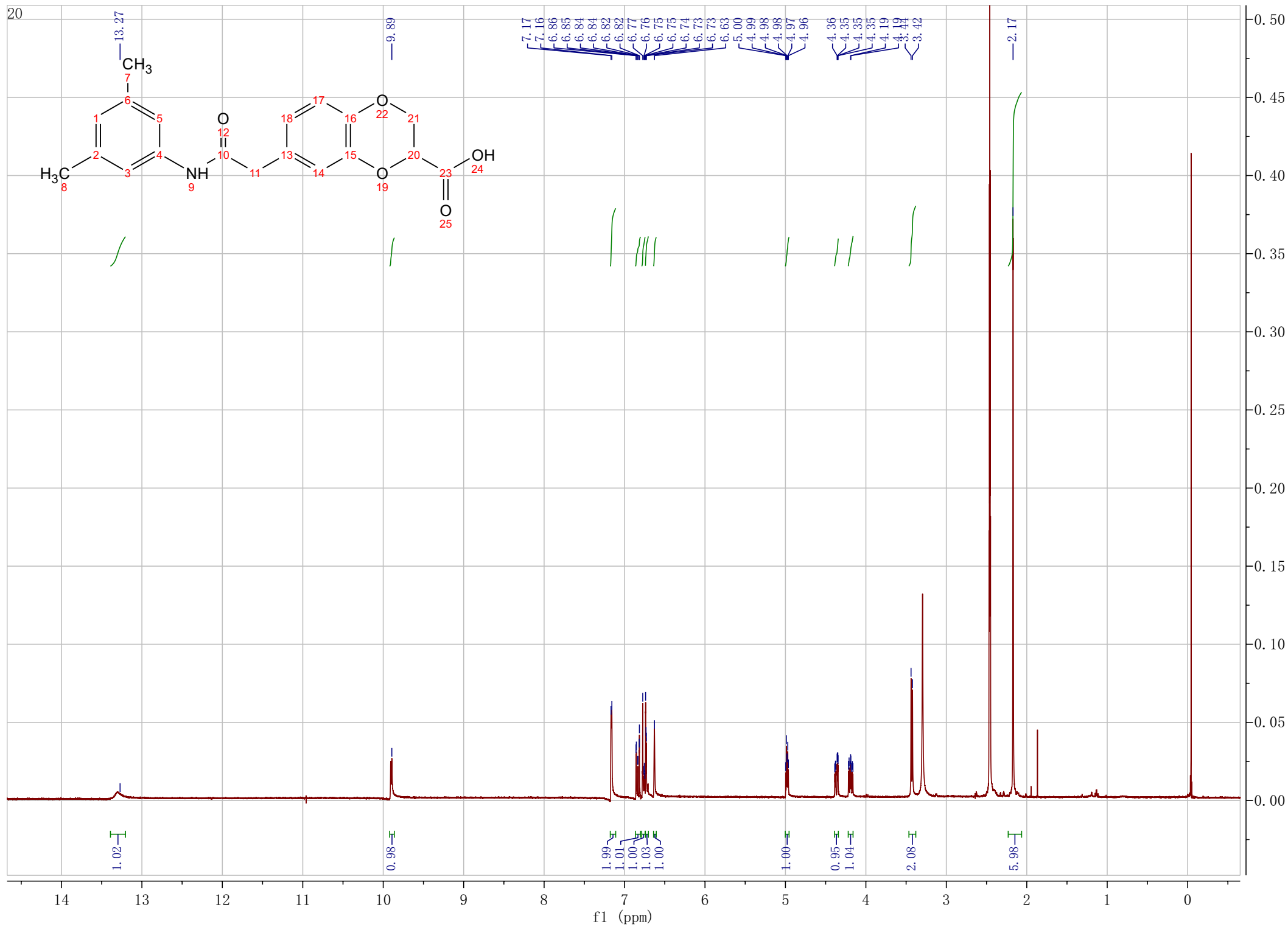

24a

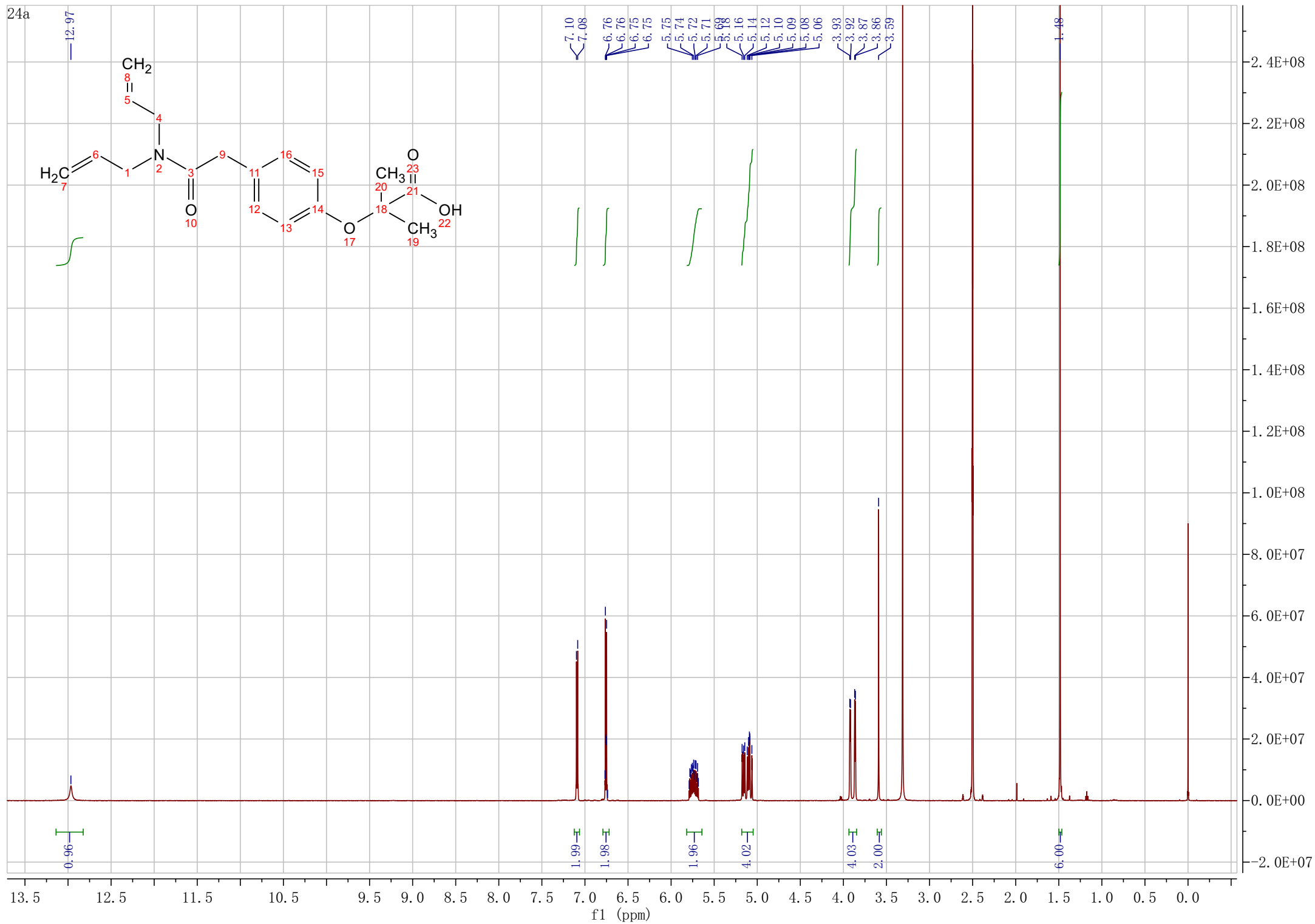

24b

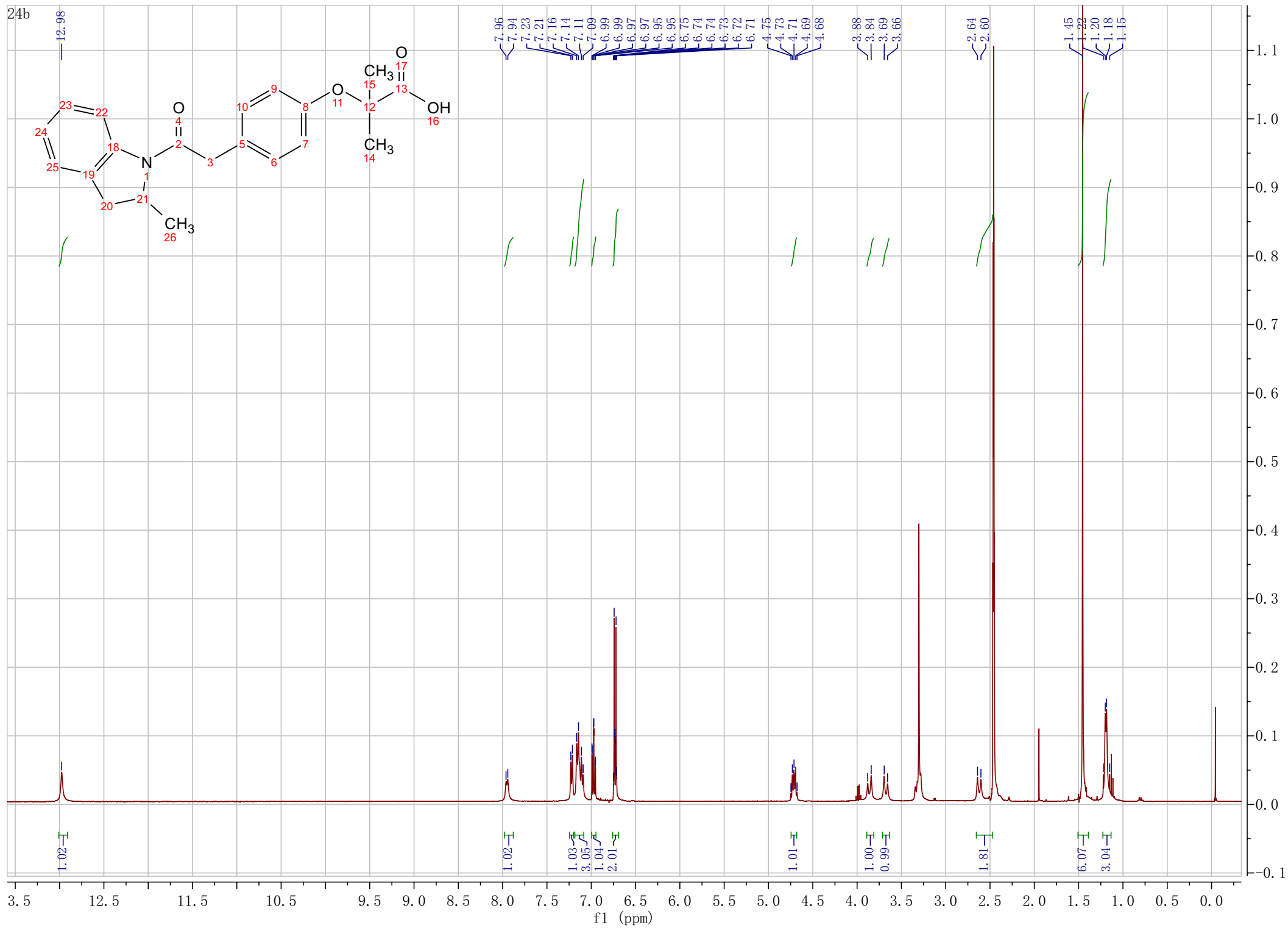

24c

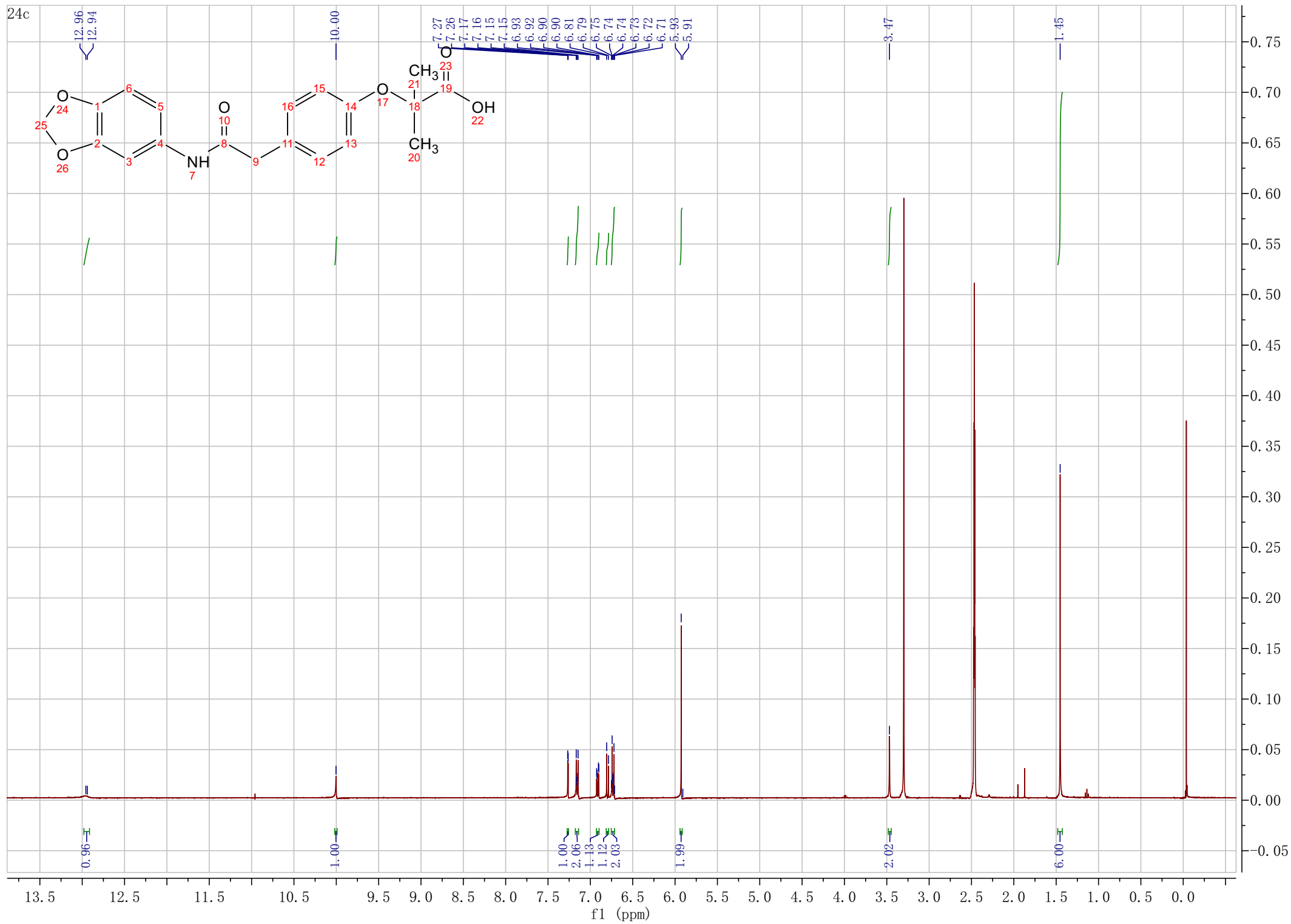

24d

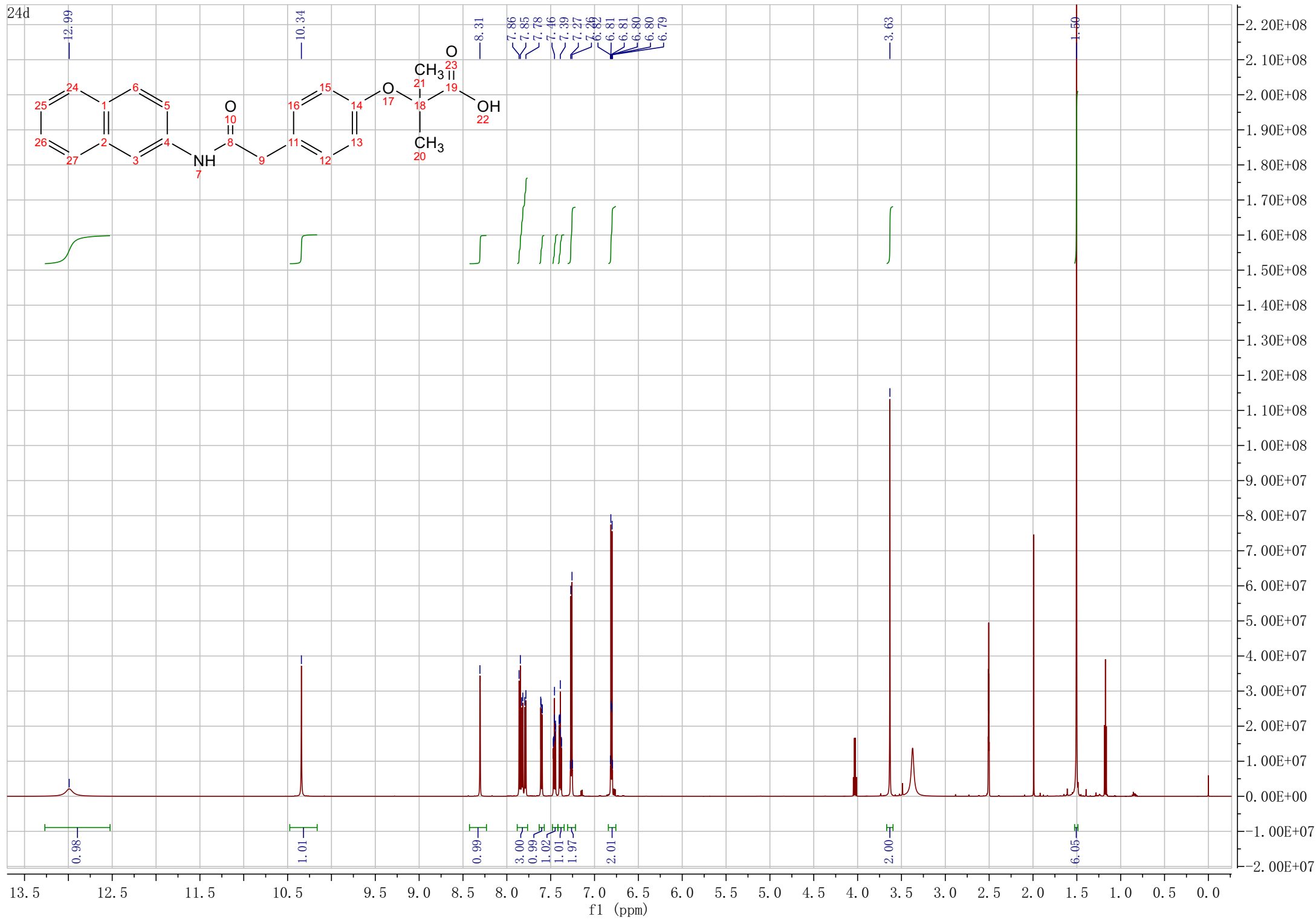

24e

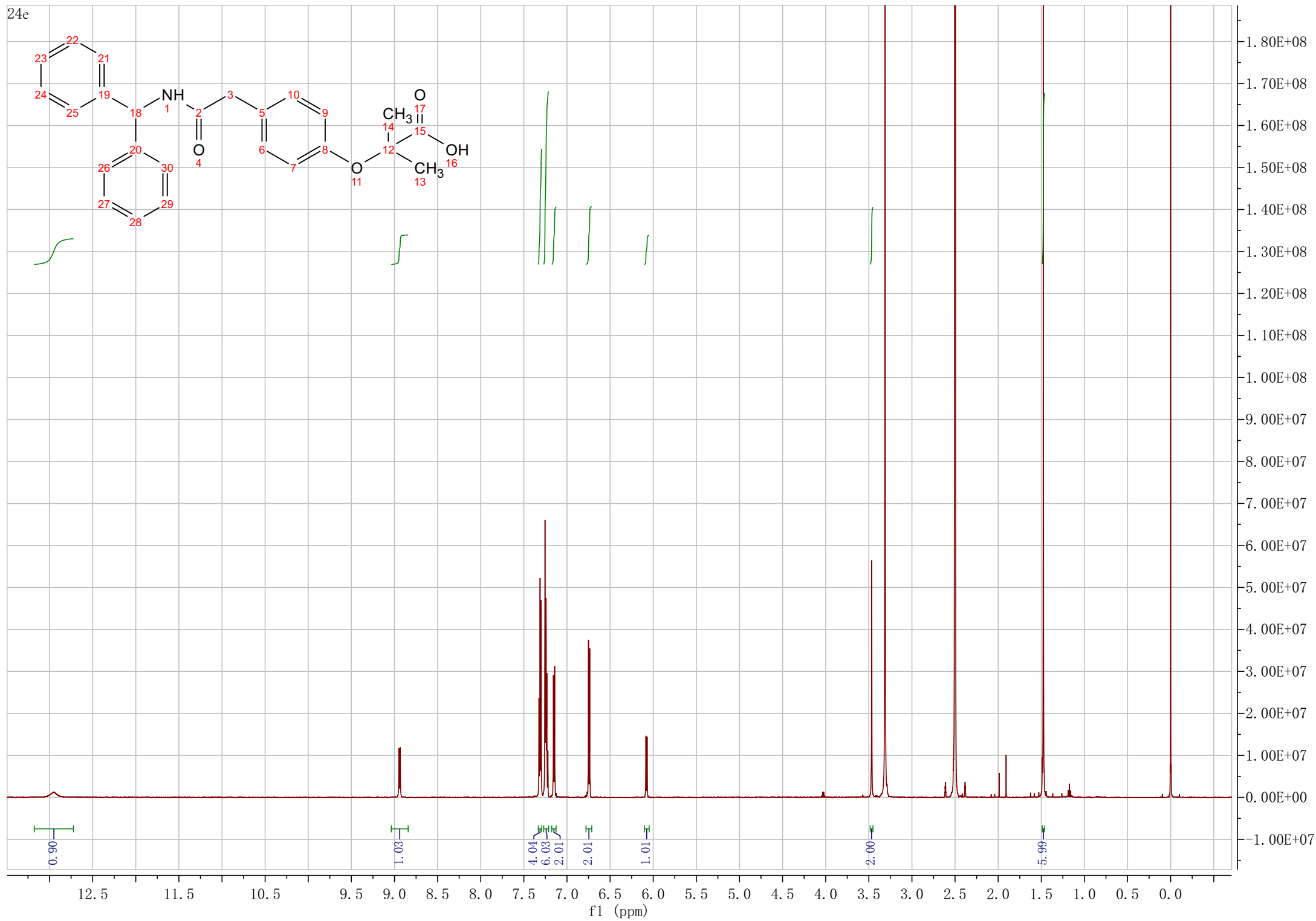

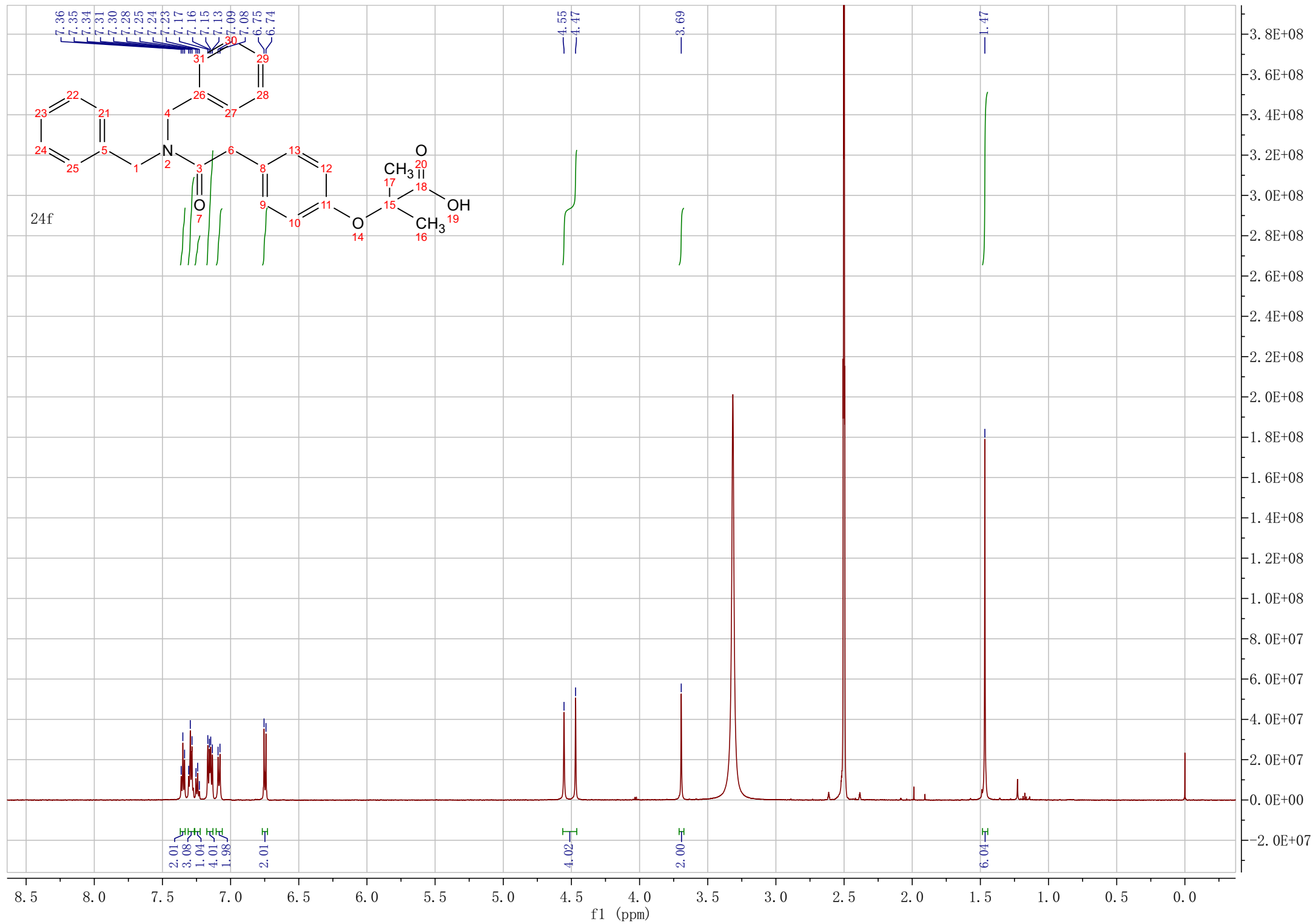

24g

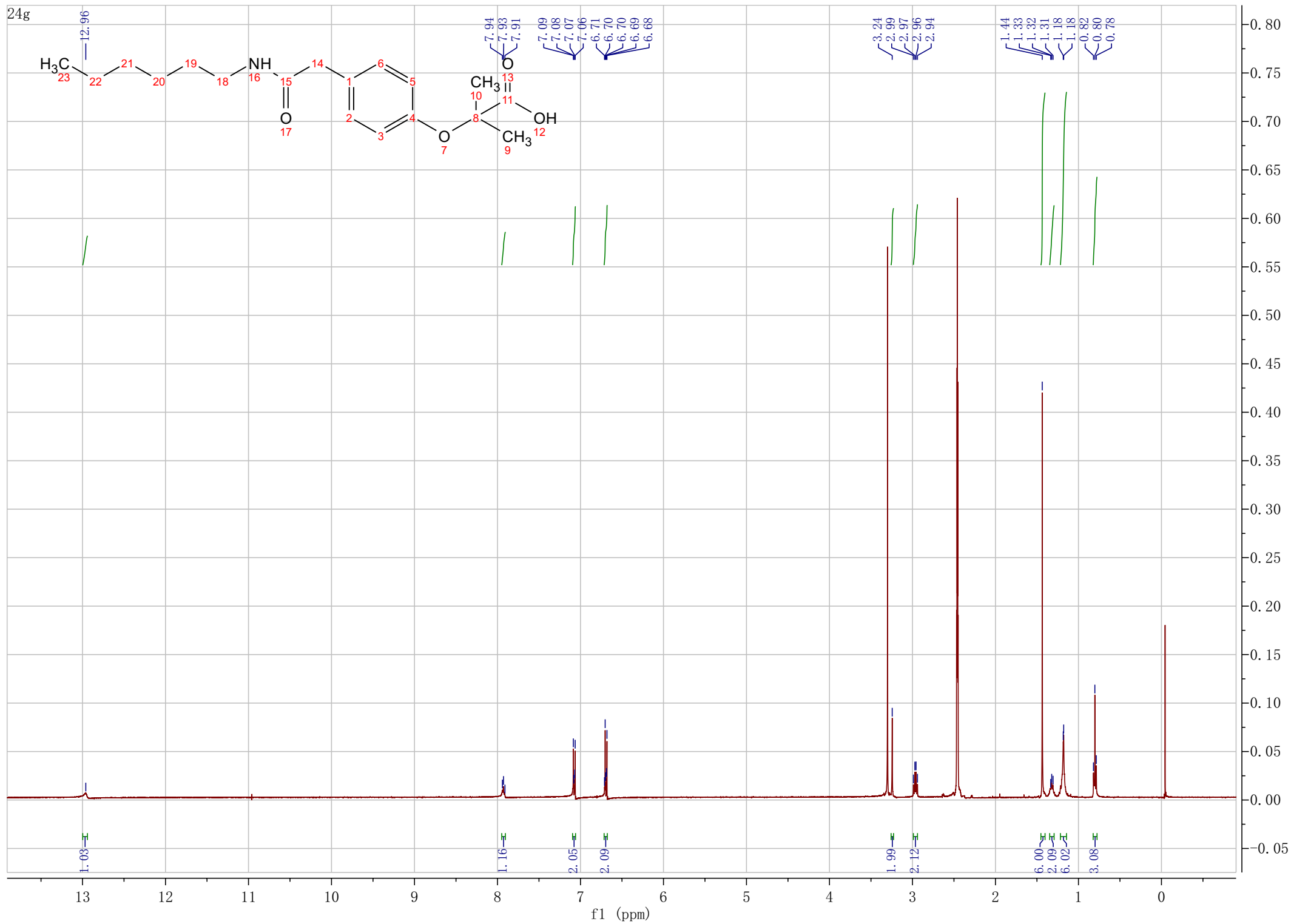

24h

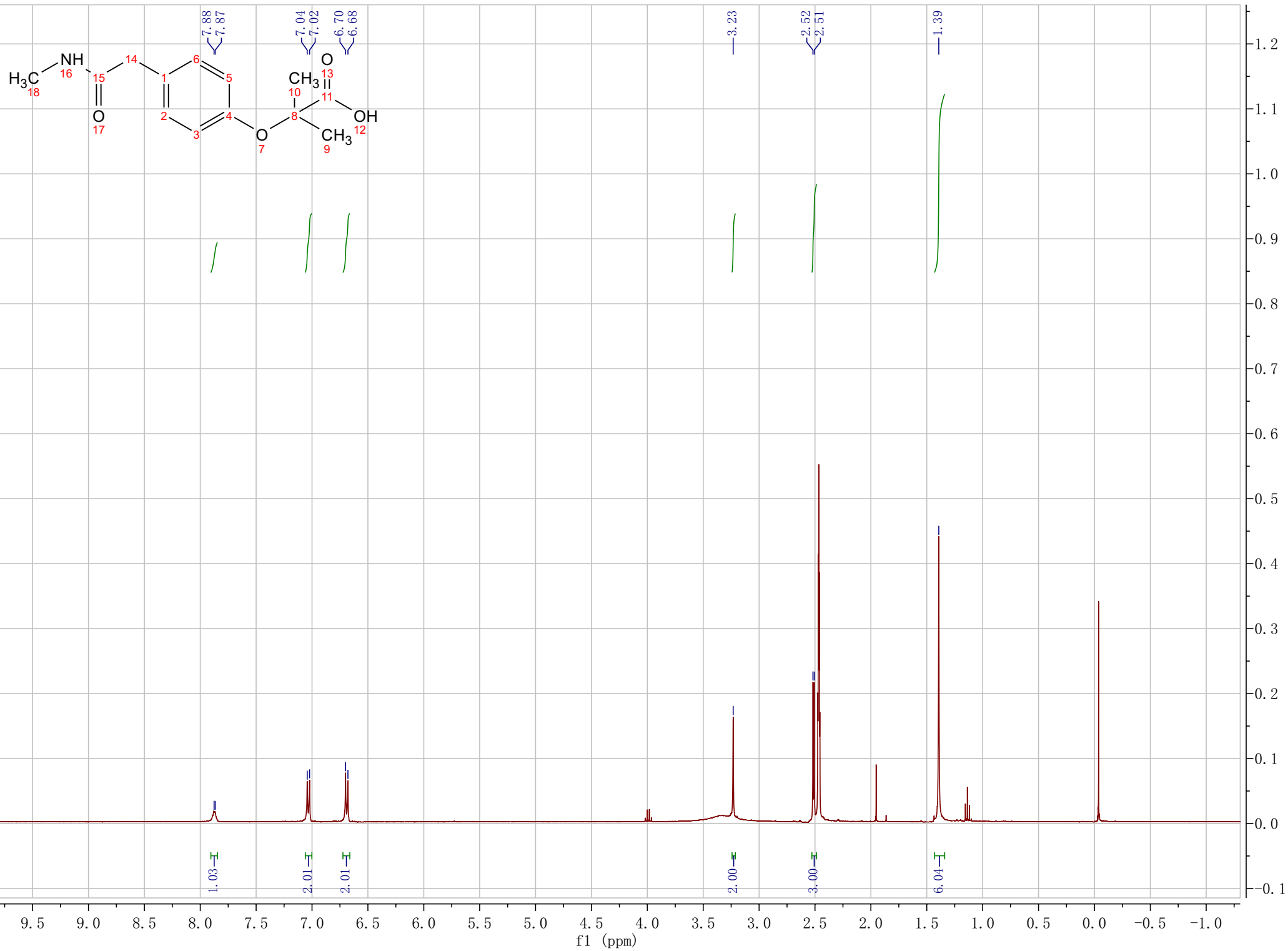

Supplement: Supplementary file 1 [file molecules-27-02428-s001.zip › Figure S2 .pdf]

15a

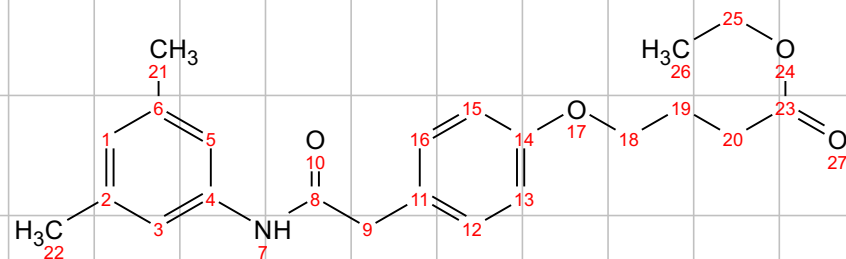

173.02

169.79

157.66

139.56

138.09

130.49

128.53

125.15

117.34

114.73

66.89

60.34

42.97

30.61

24.69

21.54

14.56

210

200

190

180

170

160

150

140

130

120

110

100

90

80

70

60

50

40

30

20

10

0

-10

f1 (ppm)

1.00E+09

9.00E+08

8.00E+08

7.00E+08

6.00E+08

5.00E+08

4.00E+08

3.00E+08

2.00E+08

1.00E+08

0.00E+00

15b

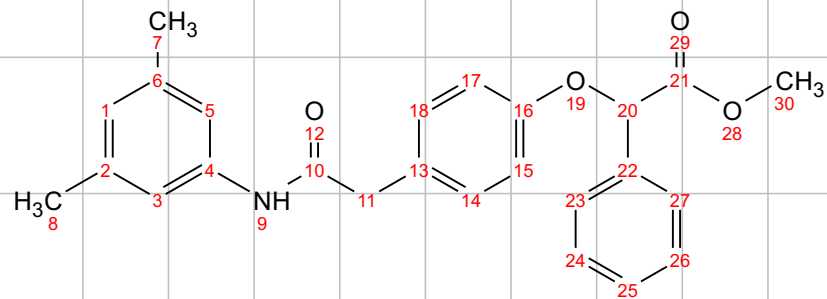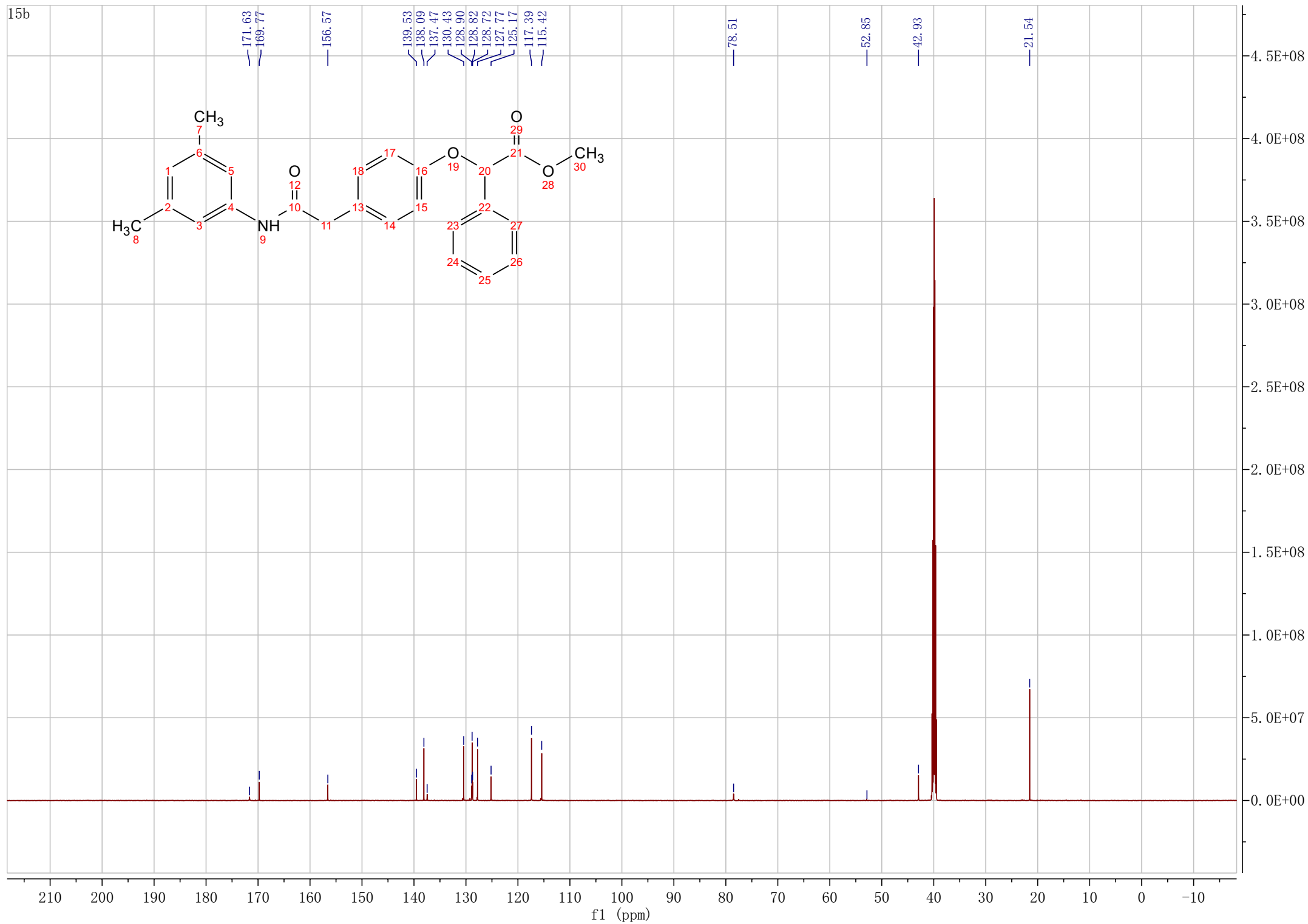

15c

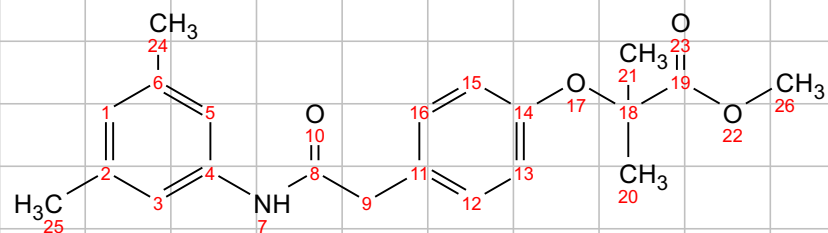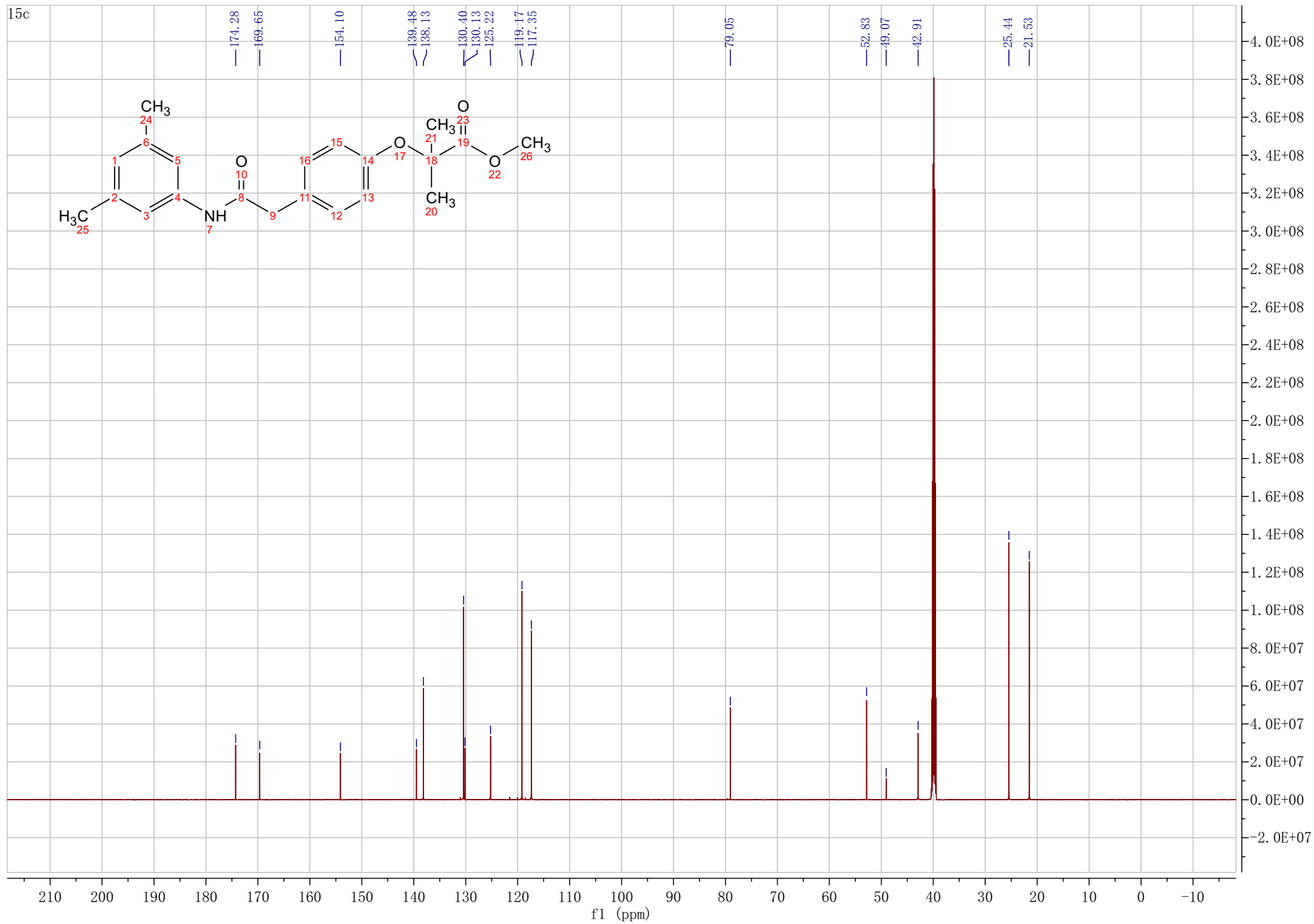

16a

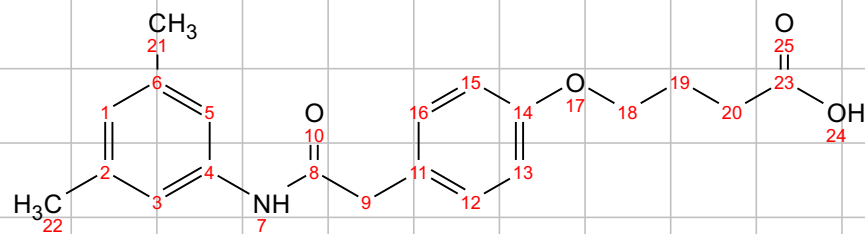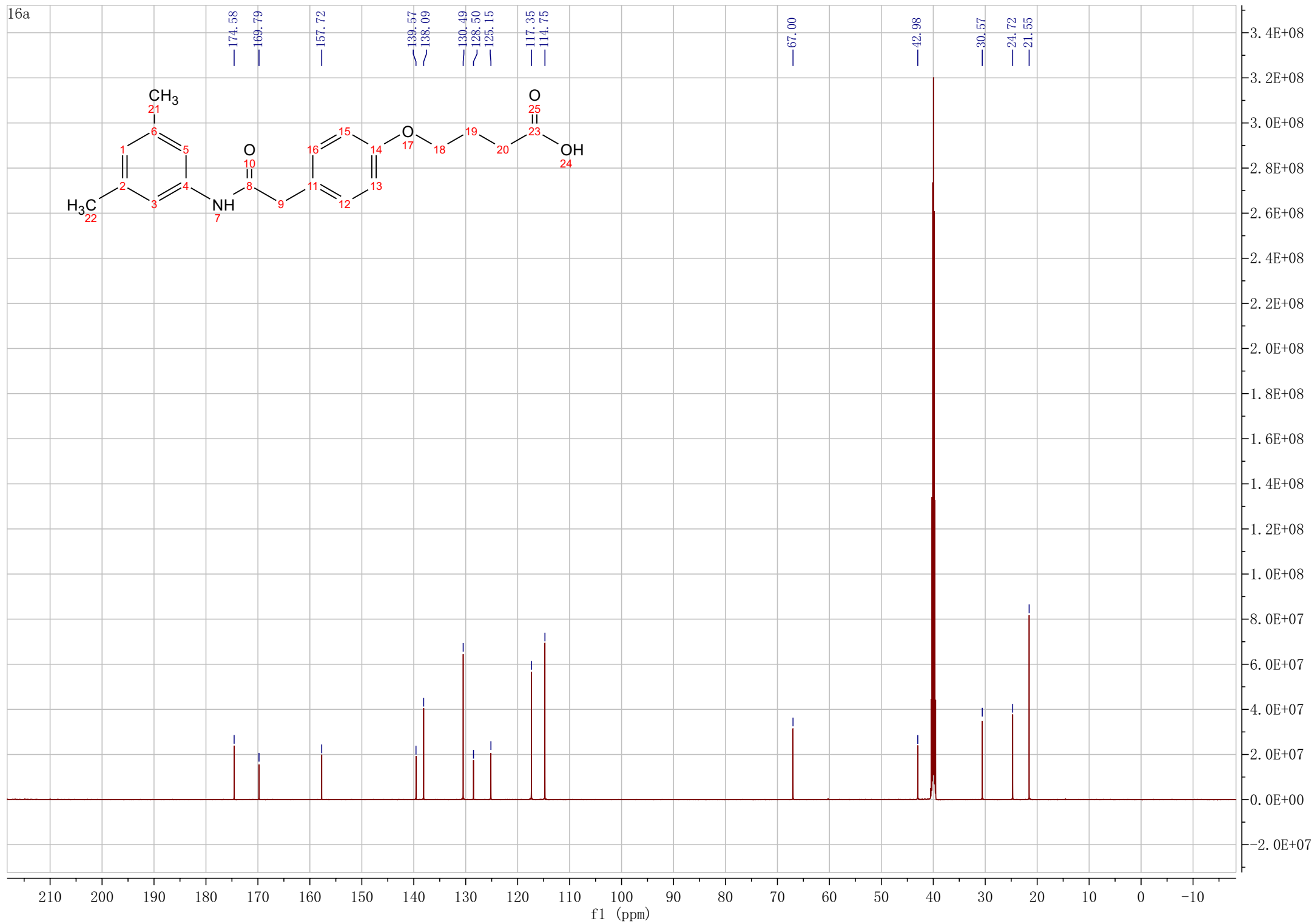

16b

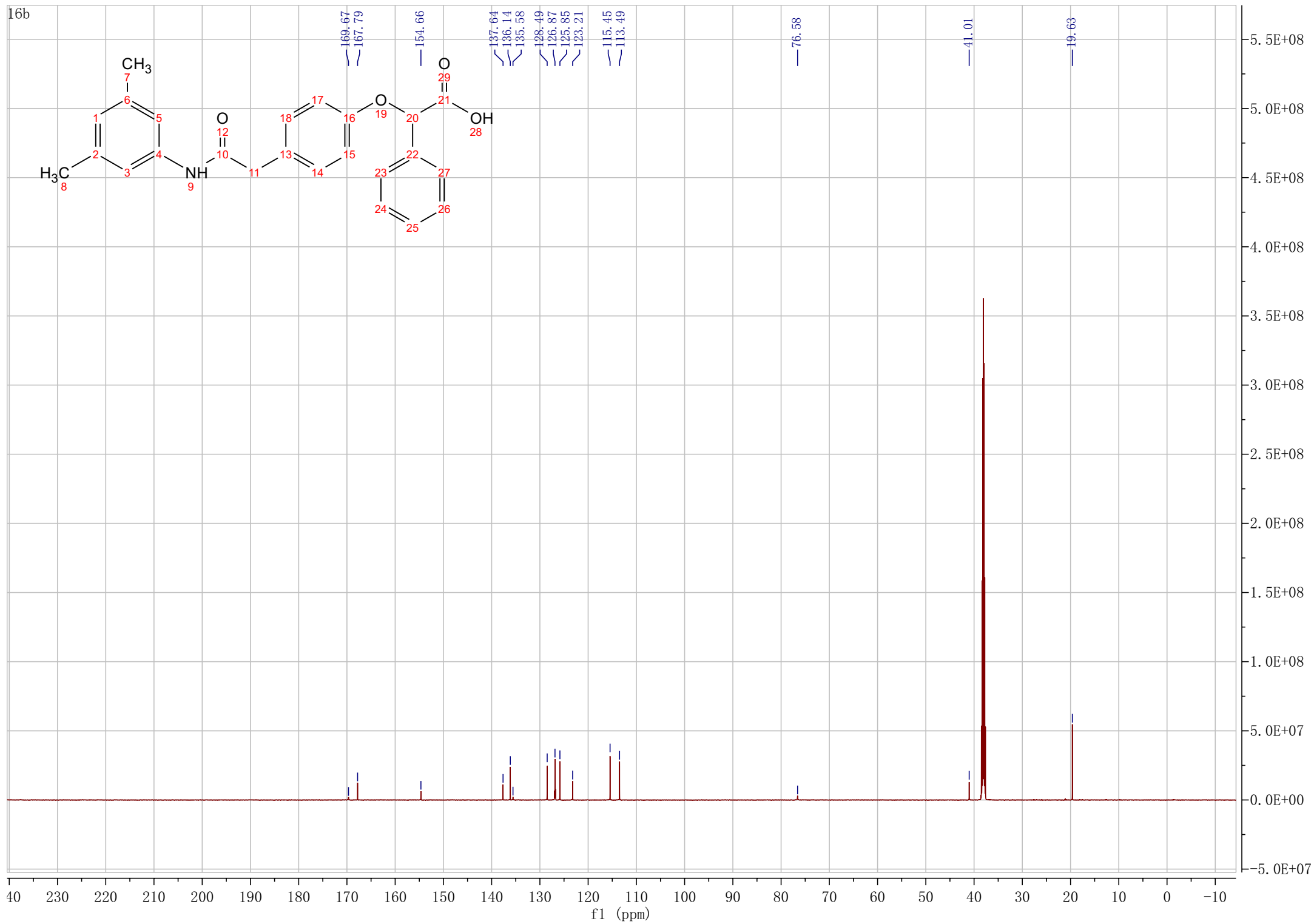

18a

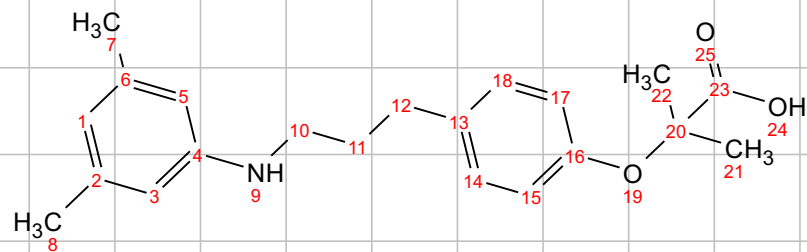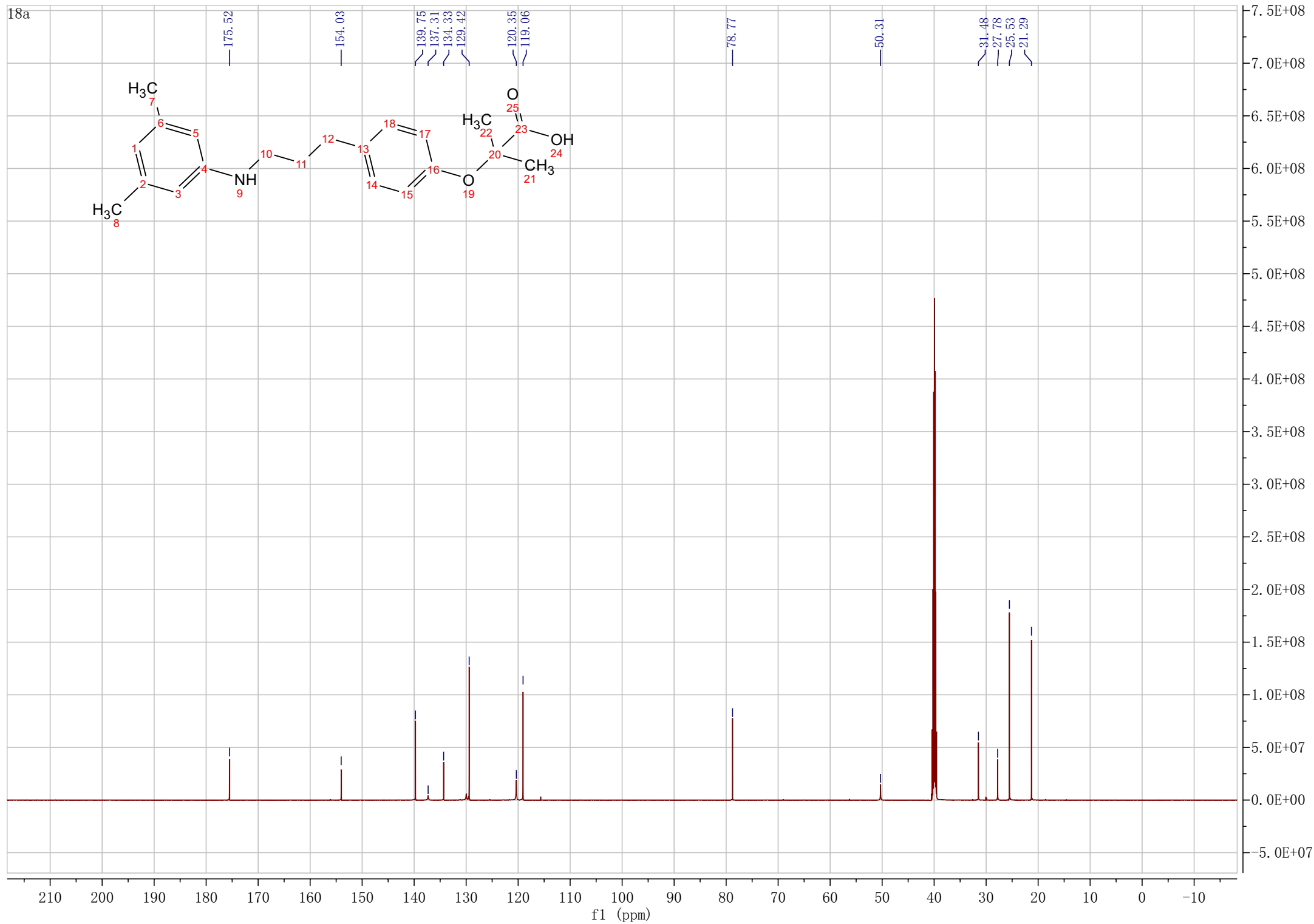

18b

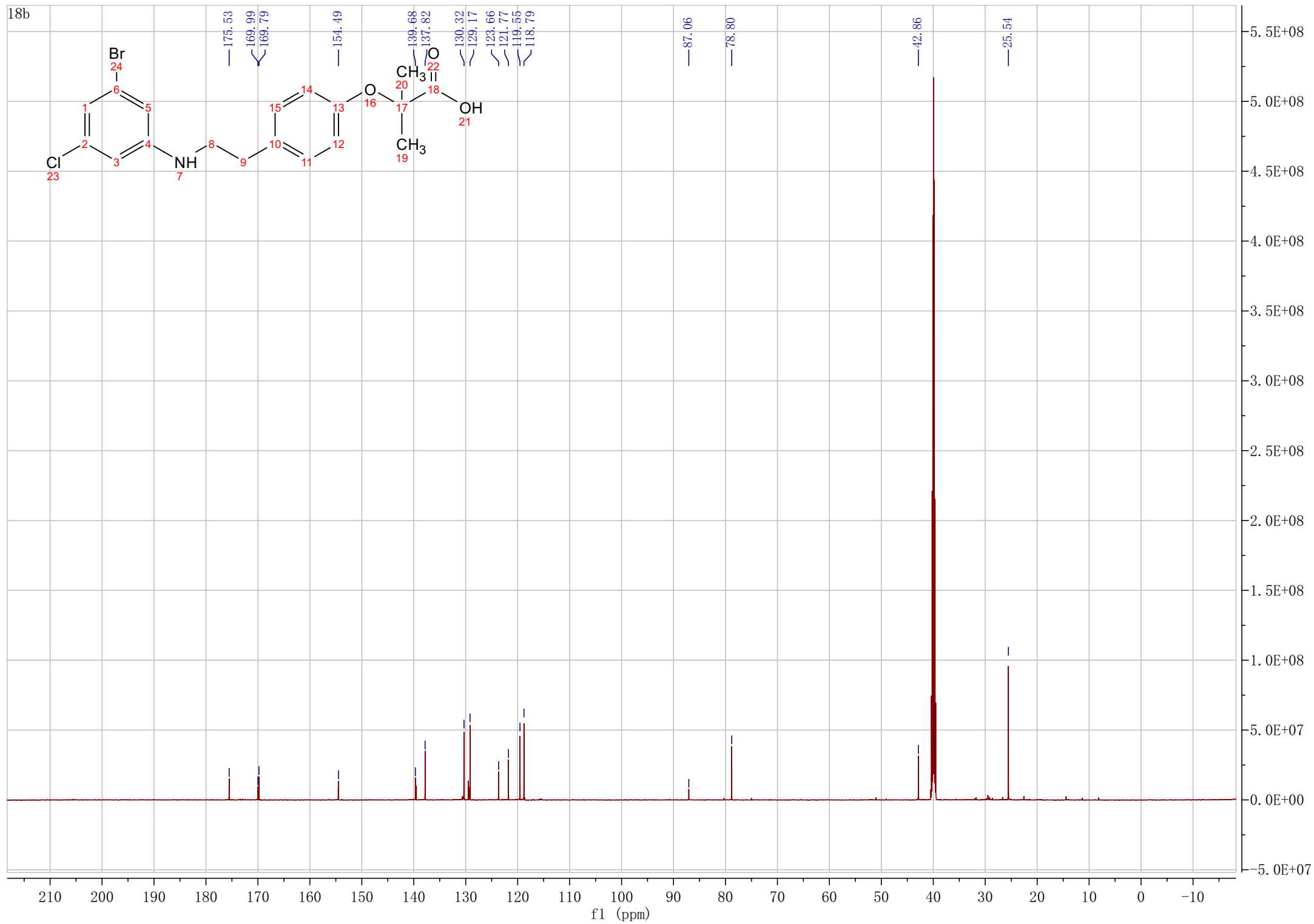

18c

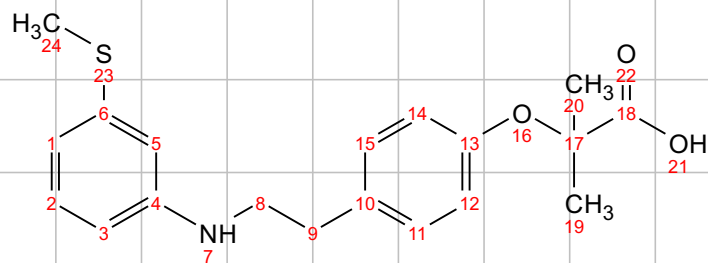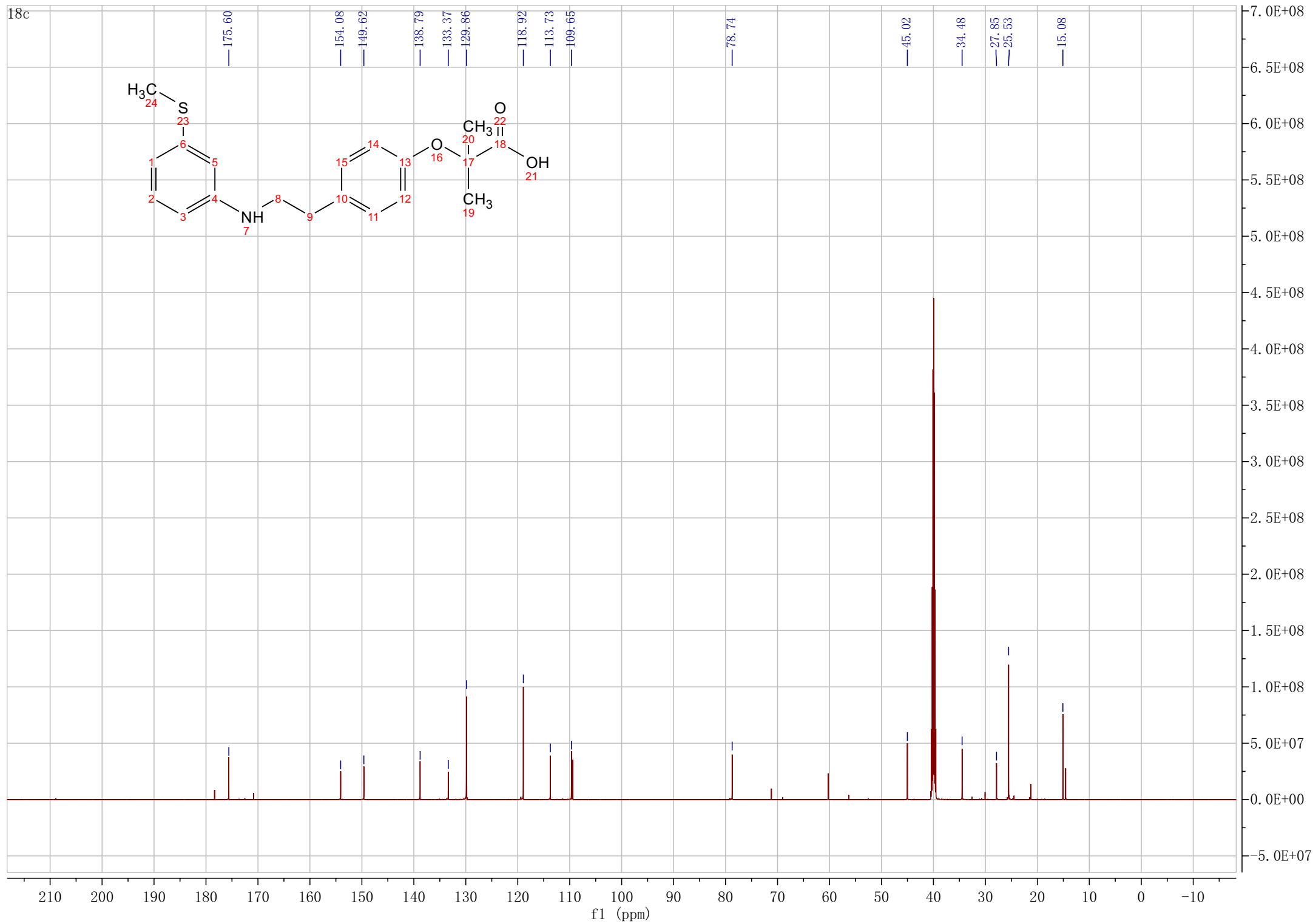

18d

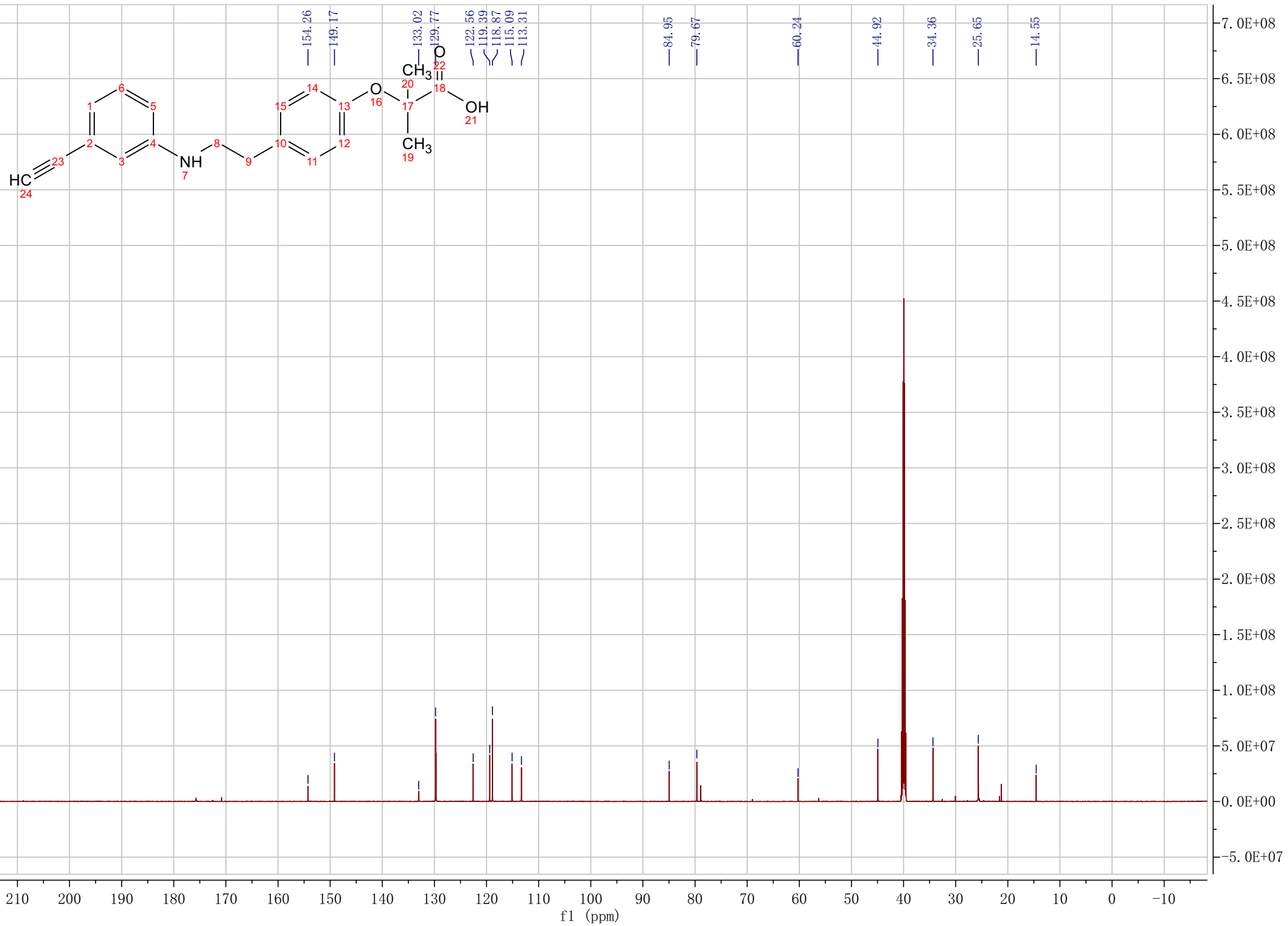

18e

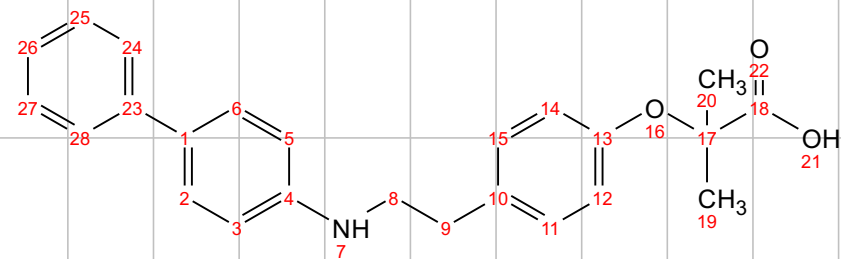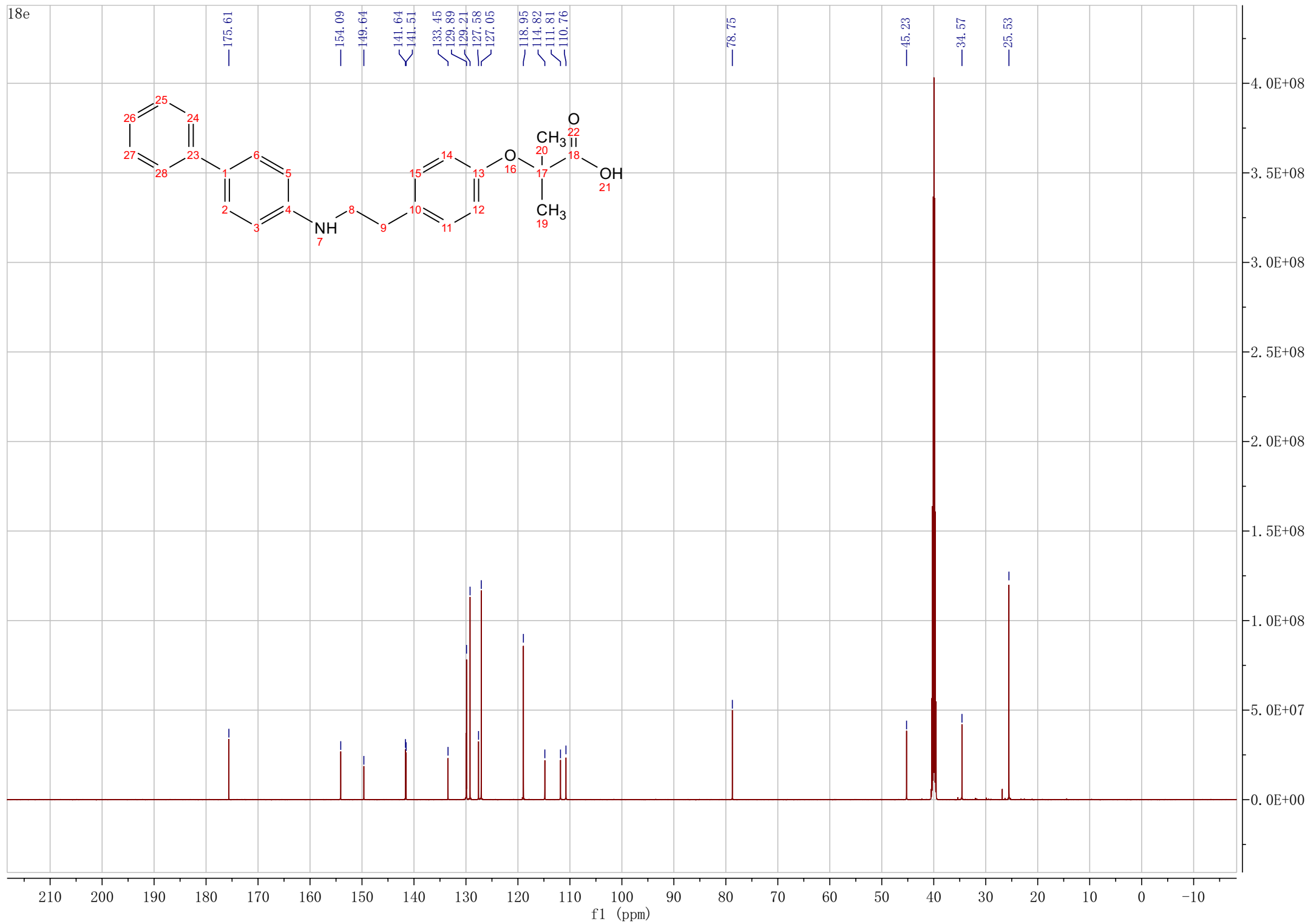

18f

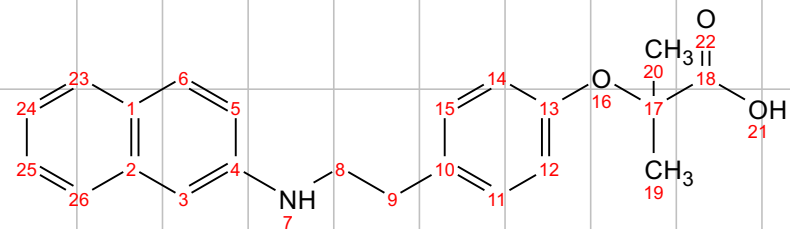

175.73

154.28

147.02

135.71

133.20

129.84

128.76

127.85

126.88

126.39

125.94

121.40

118.90

118.78

102.77

78.91

45.33

34.28

25.66

210 200 190 180 170 160 150 140 130 120 110 100 90 80 70 60 50 40 30 20 10 0 -10

f1 (ppm)

18g

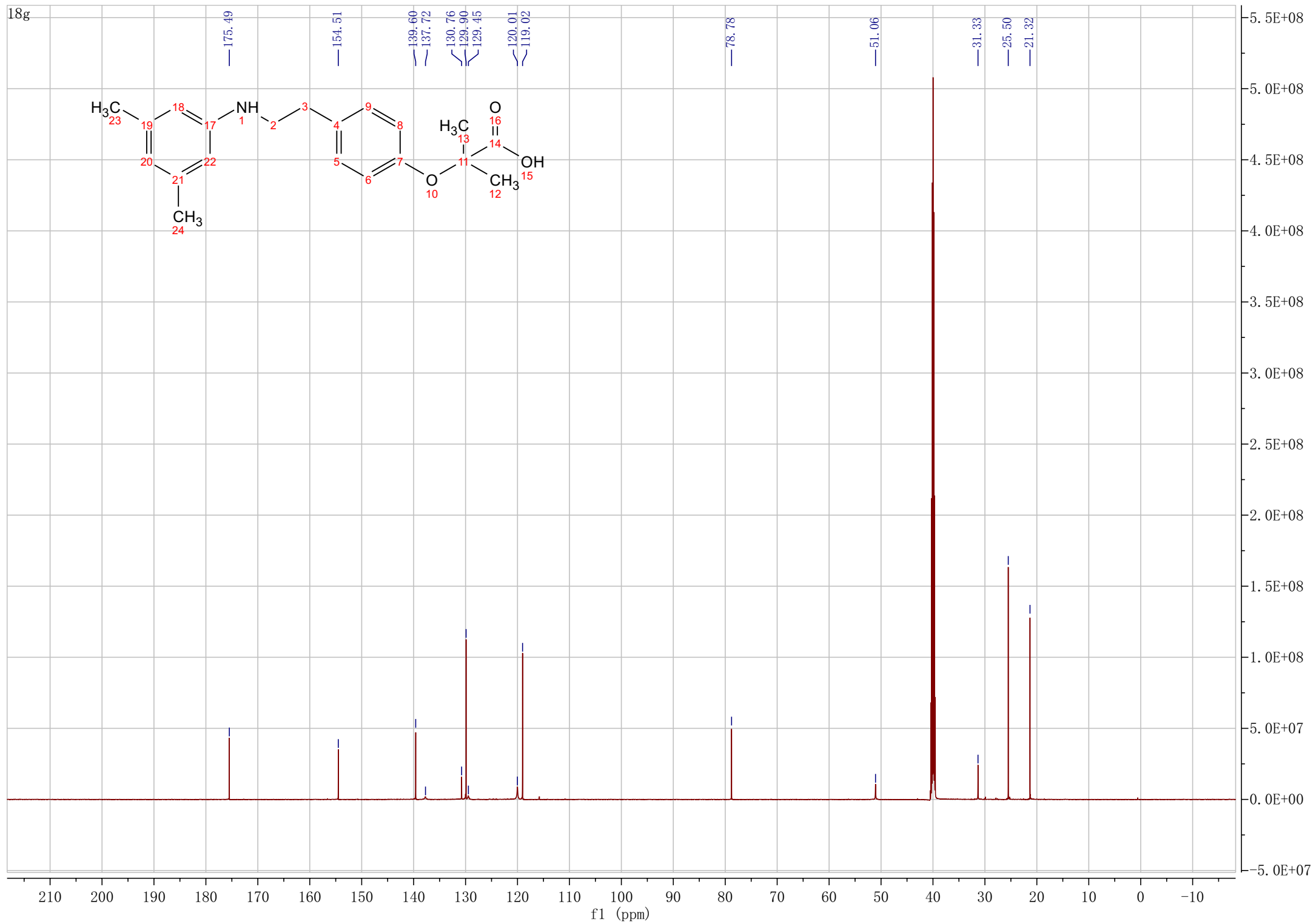

19a

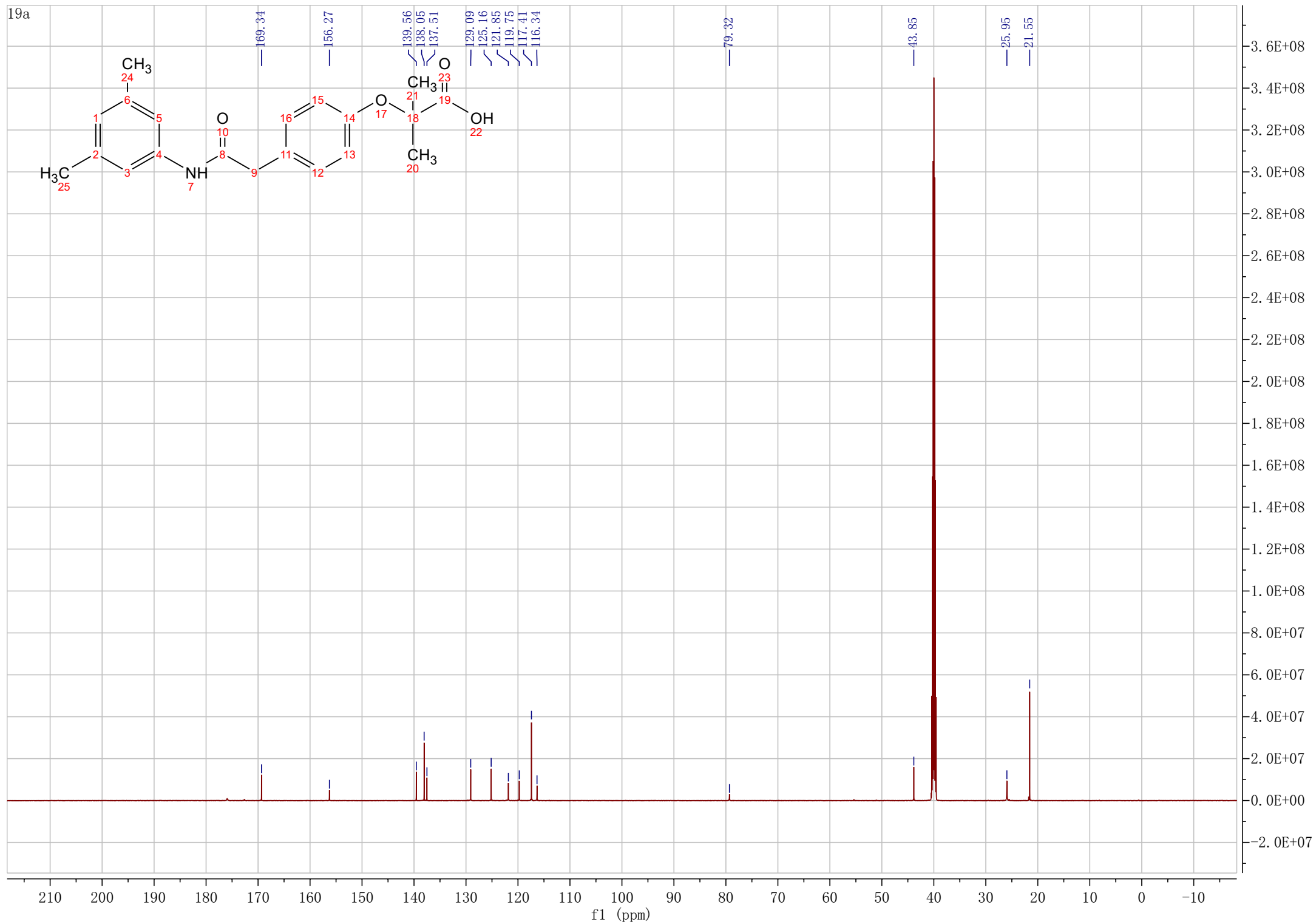

19b

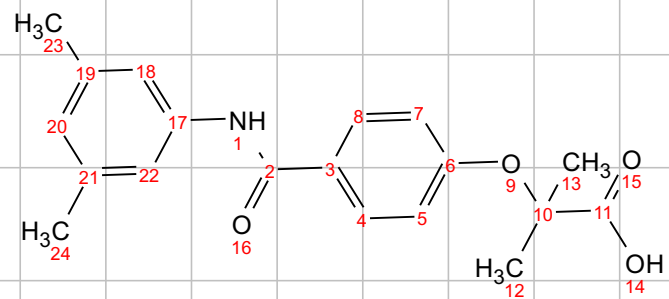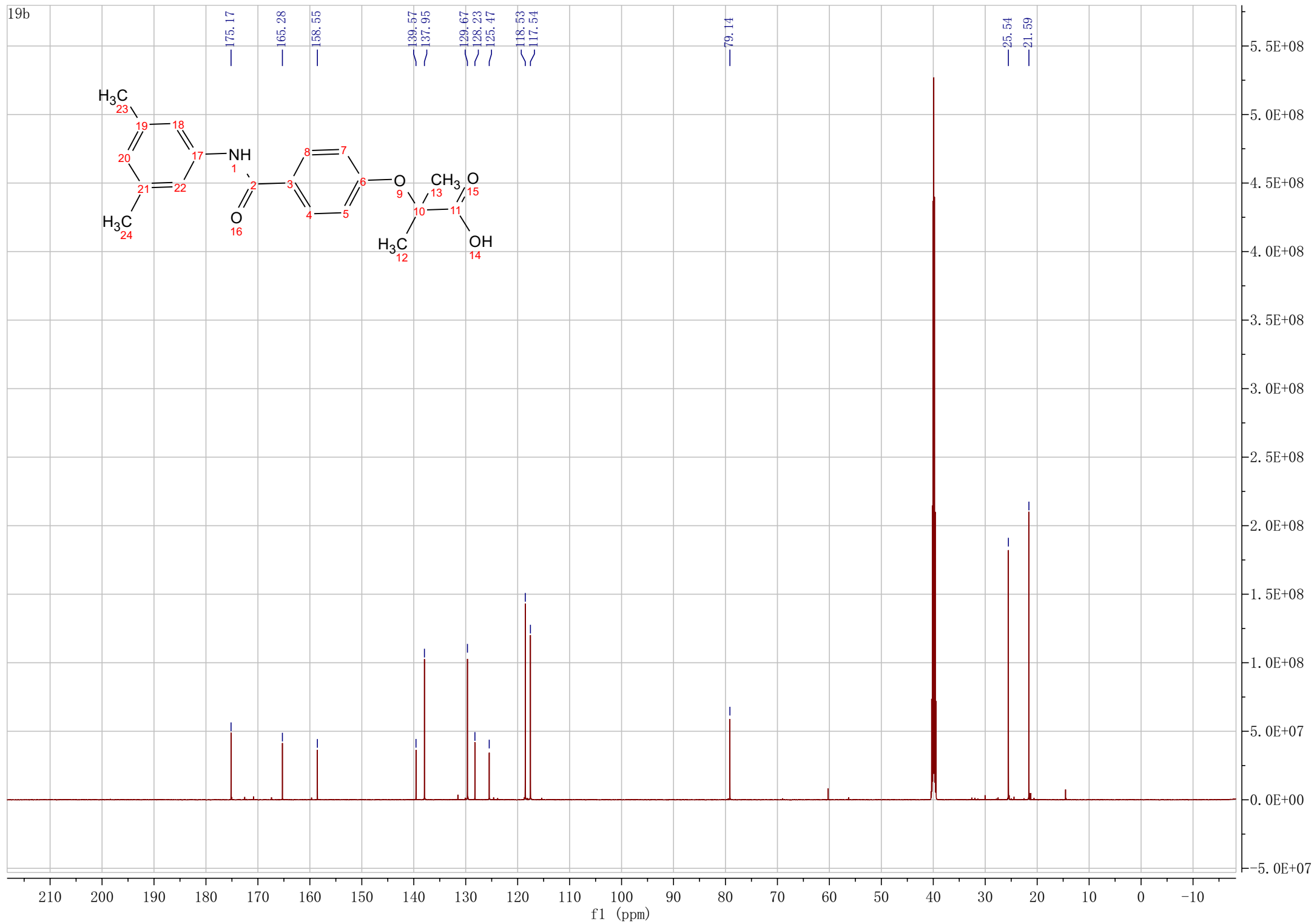

19c

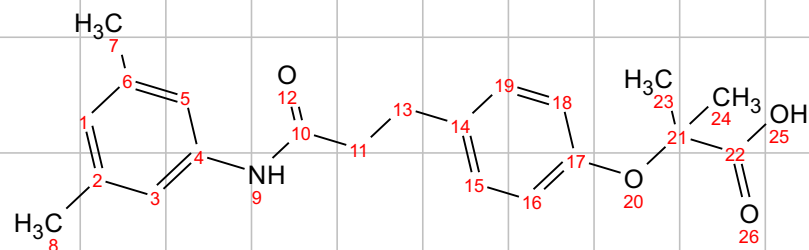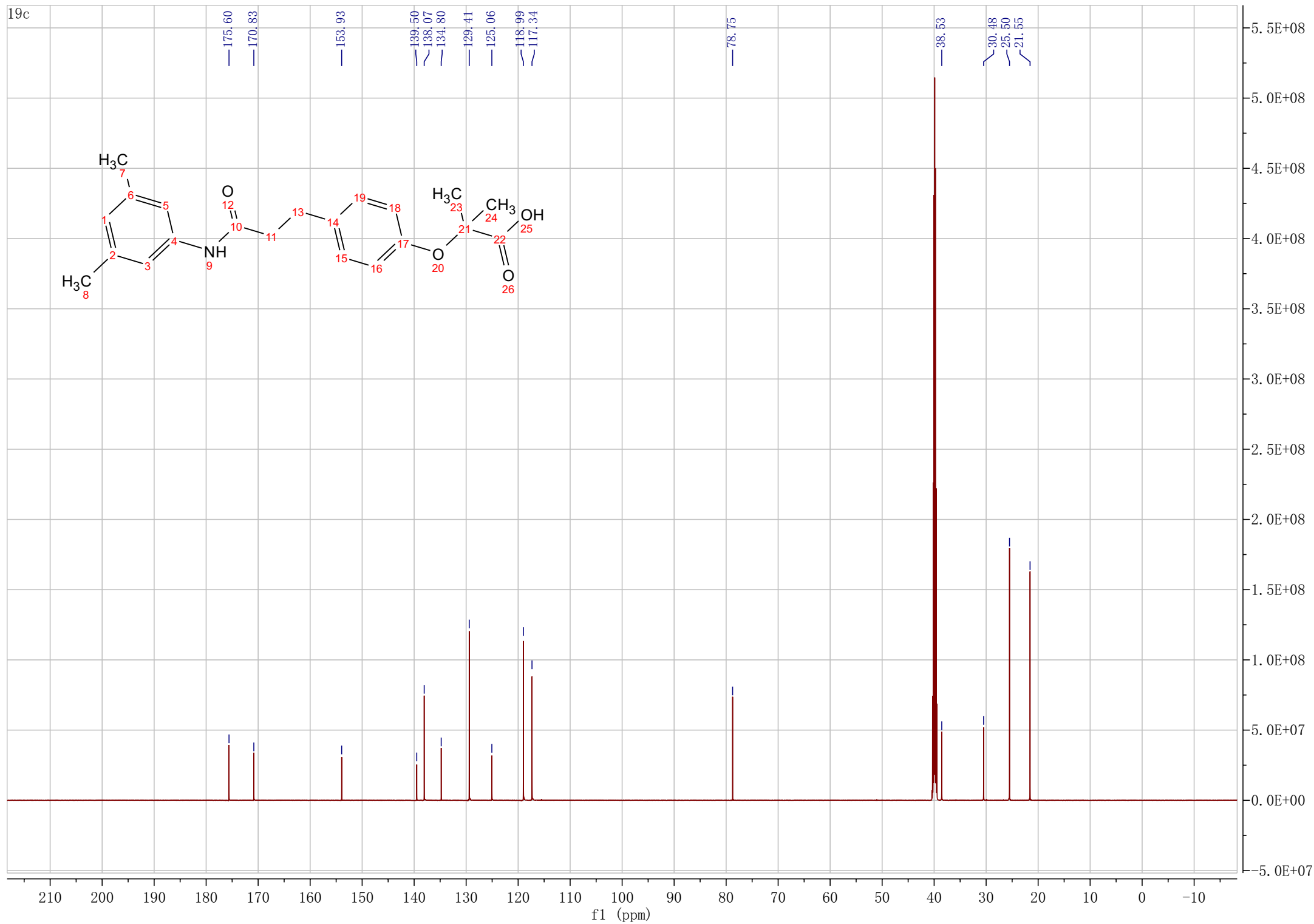

19d

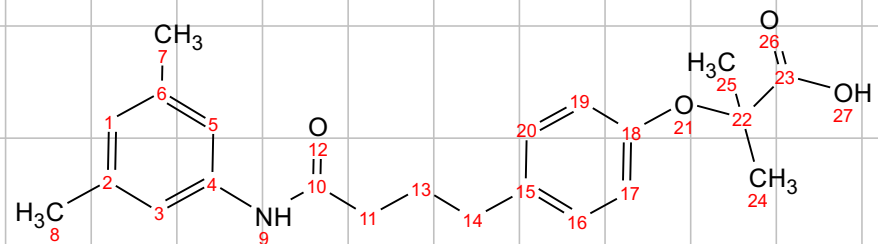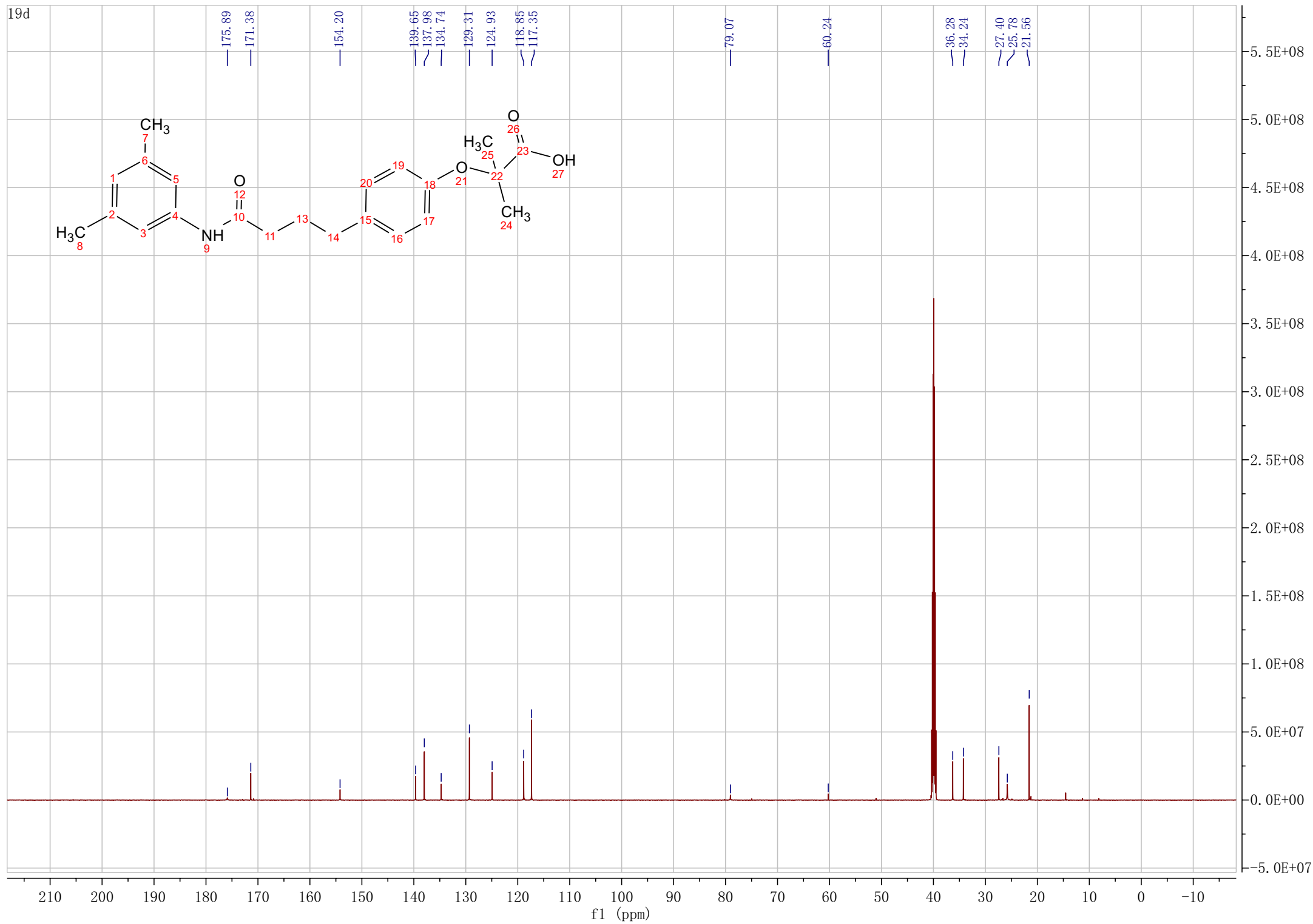

19e

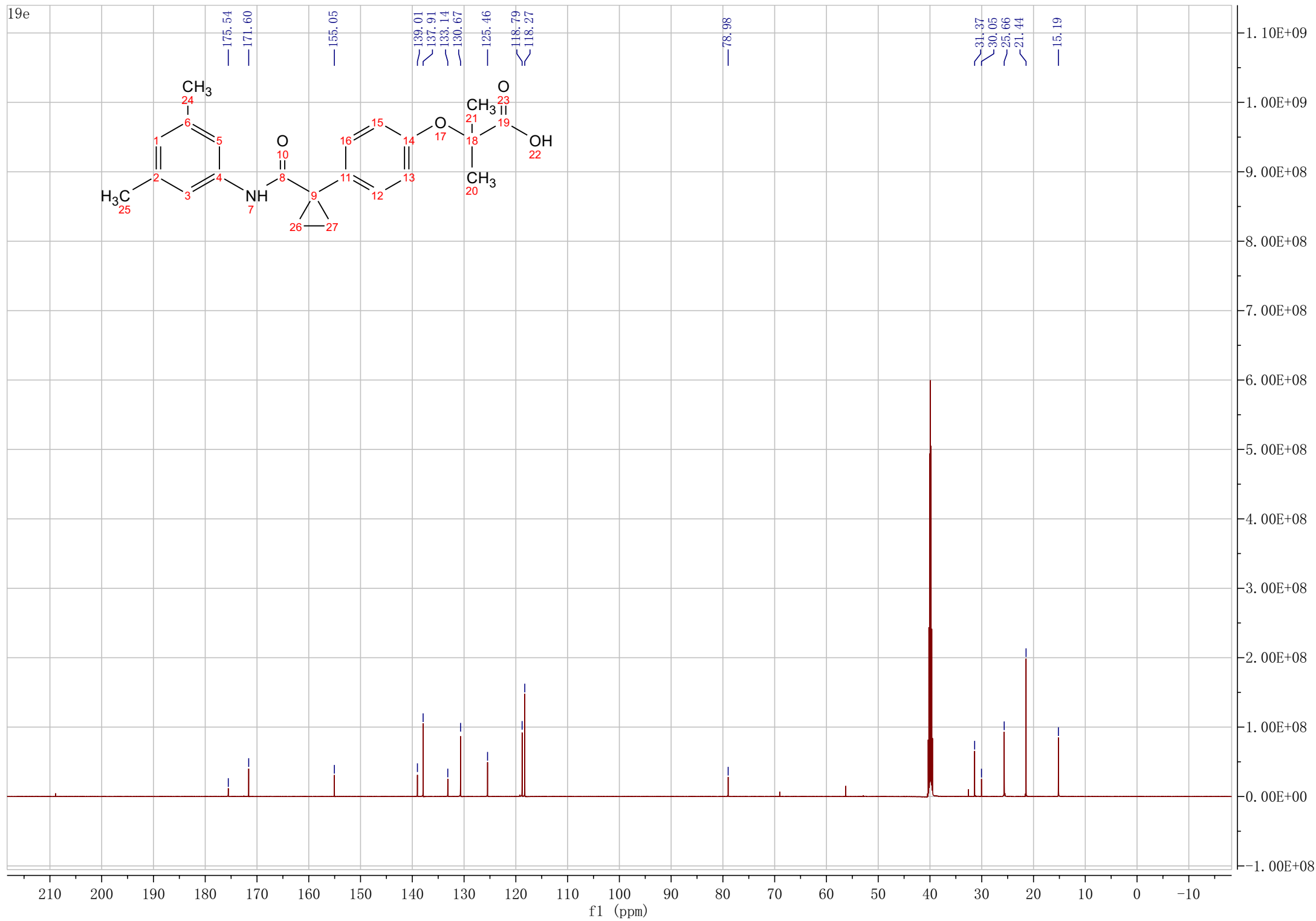

19f

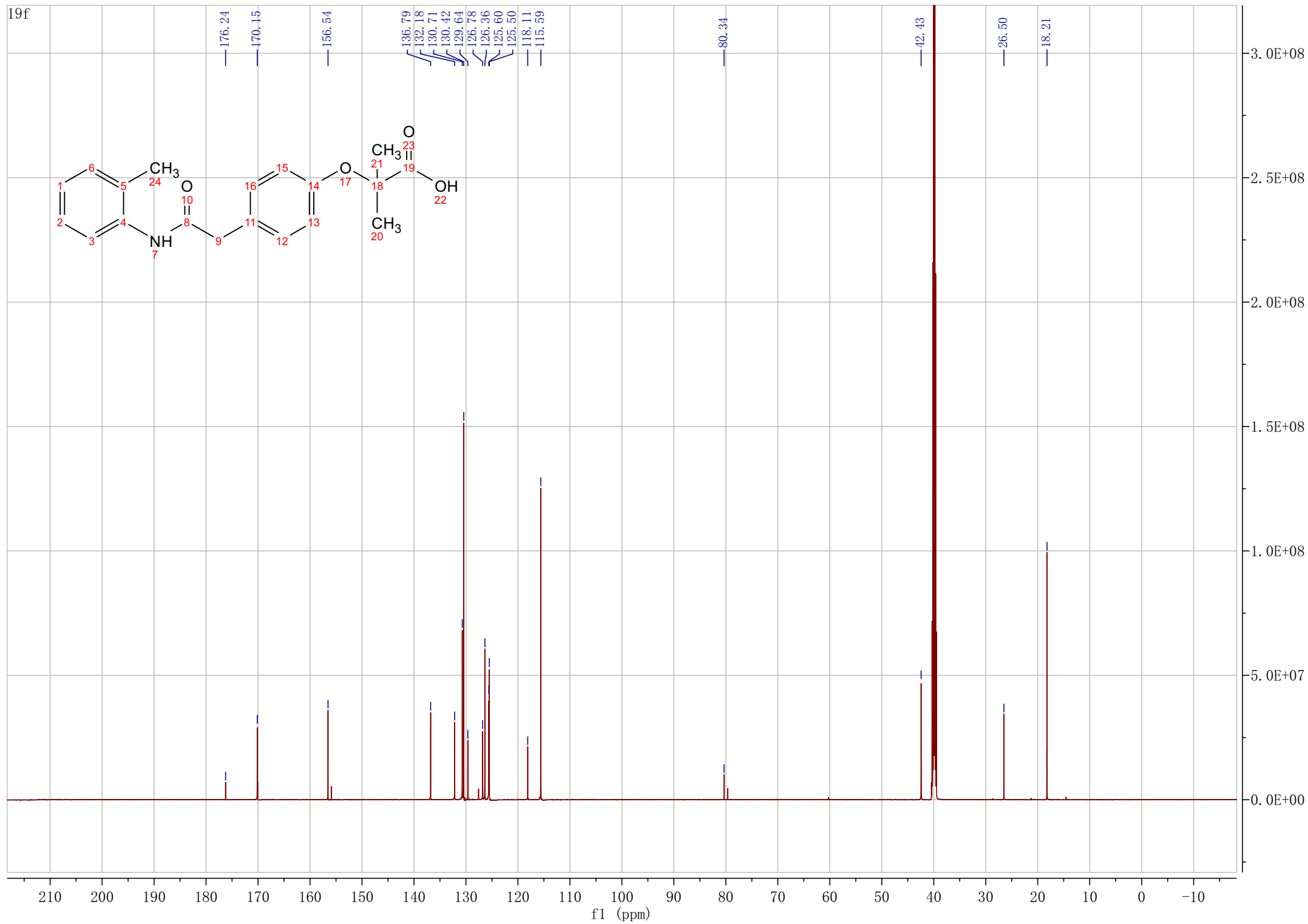

19g

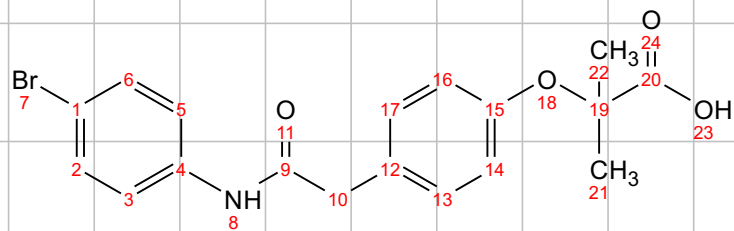

176.35  
170.09  
154.90  
139.13  
131.95  
130.14  
128.67  
121.47  
118.66  
115.12

79.10

42.87

25.81

210 200 190 180 170 160 150 140 130 120 110 100 90 80 70 60 50 40 30 20 10 0 -10

f1 (ppm)

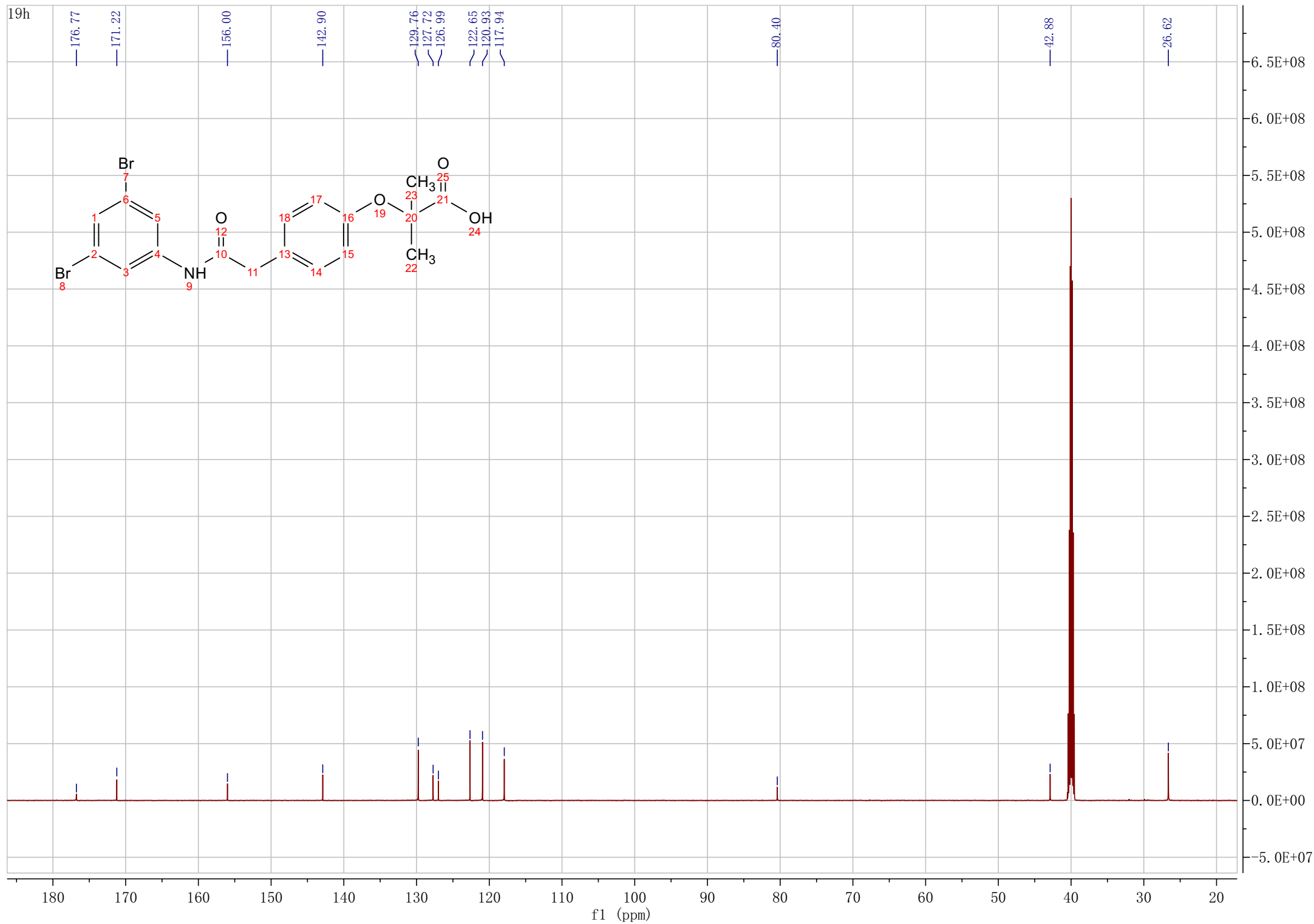

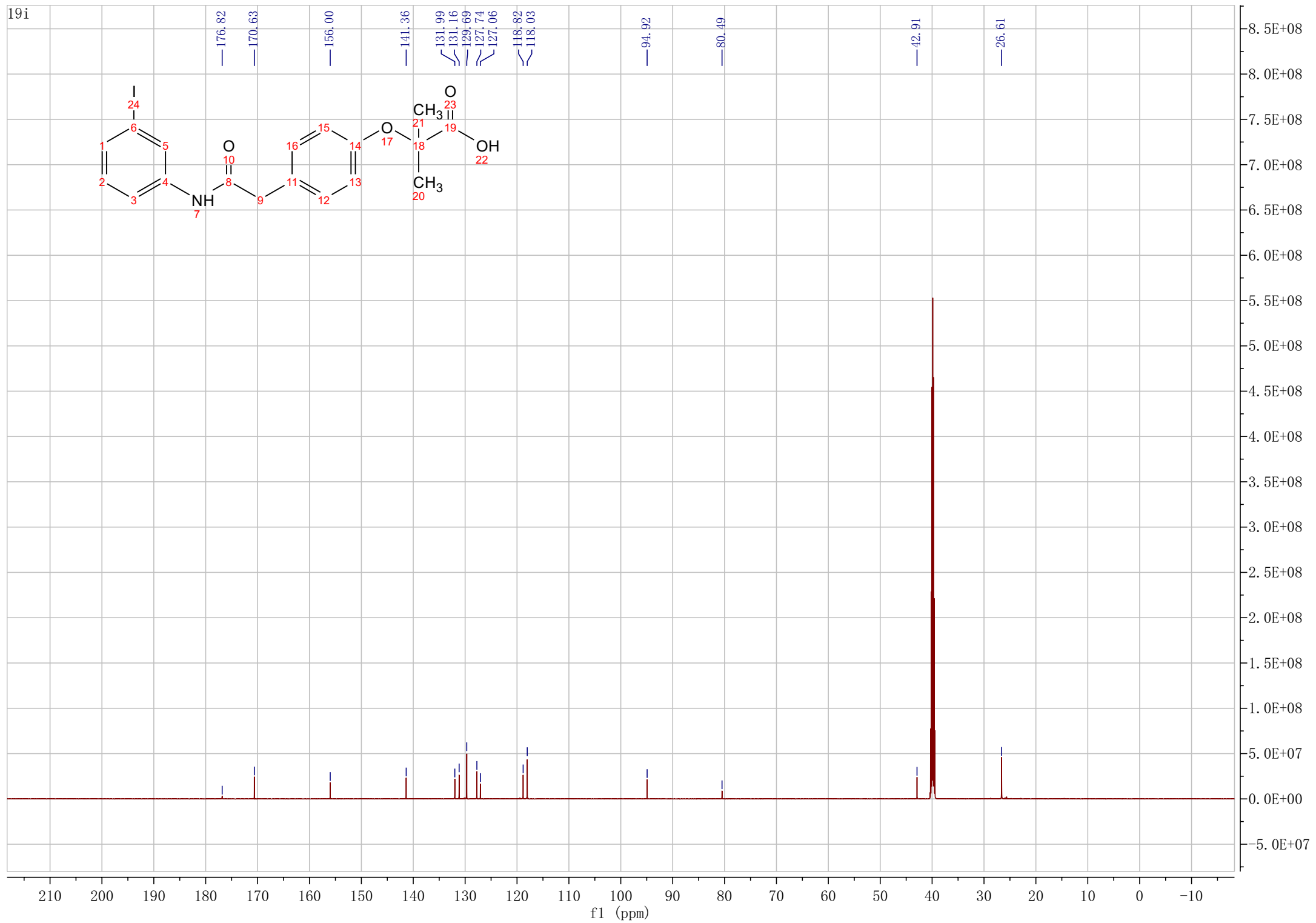

19j

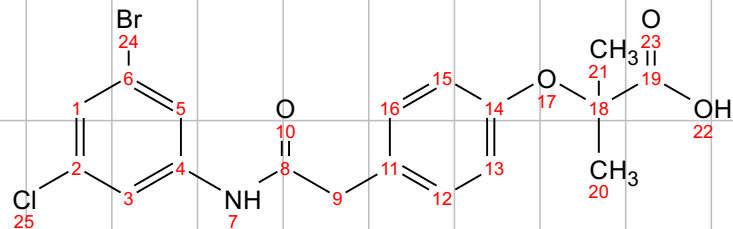

175.53  
170.61  
154.59  
142.04  
134.70  
130.41  
128.79  
125.60  
122.62  
120.49  
118.80  
118.01  
78.80  
42.82  
25.50

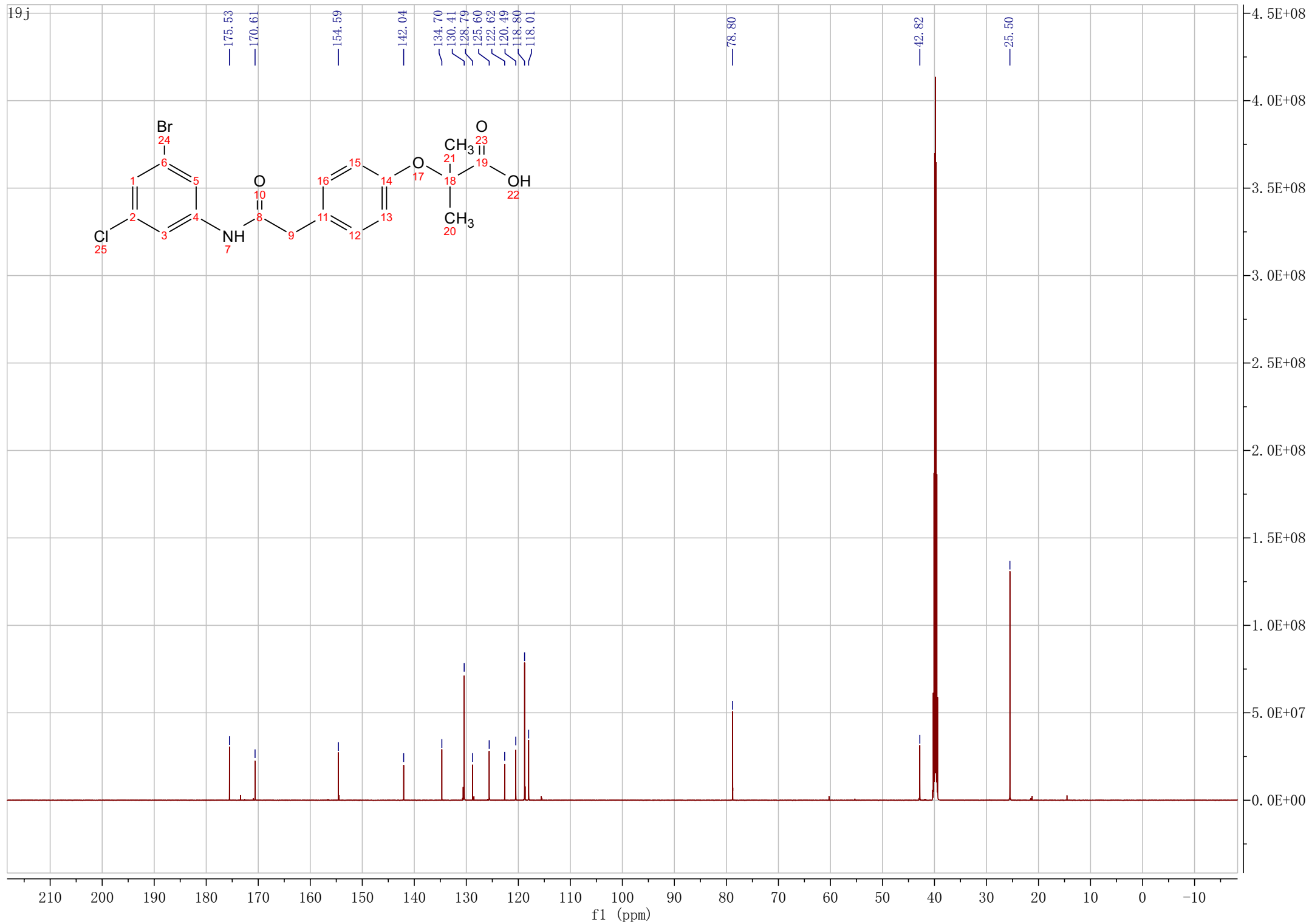

19k

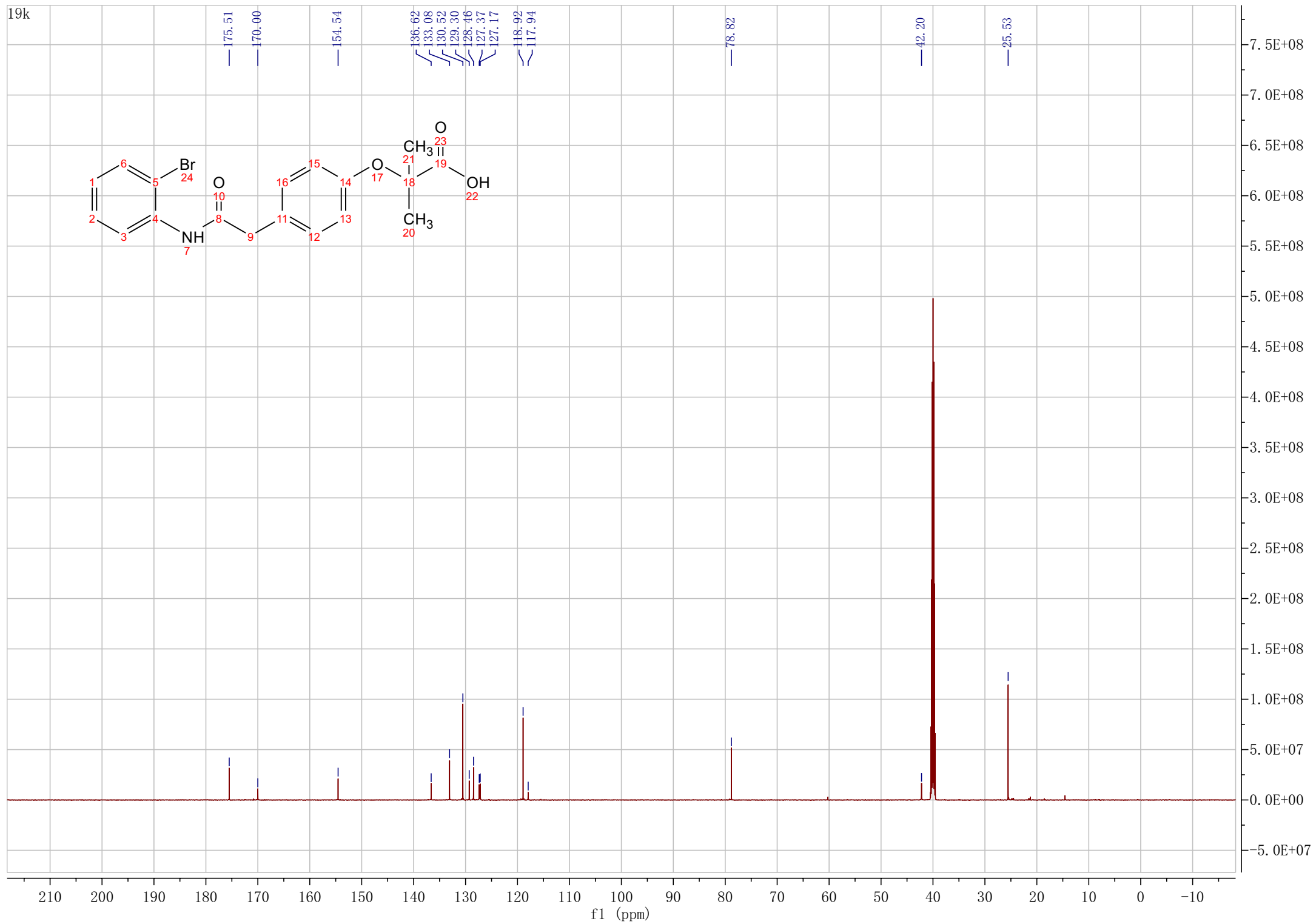

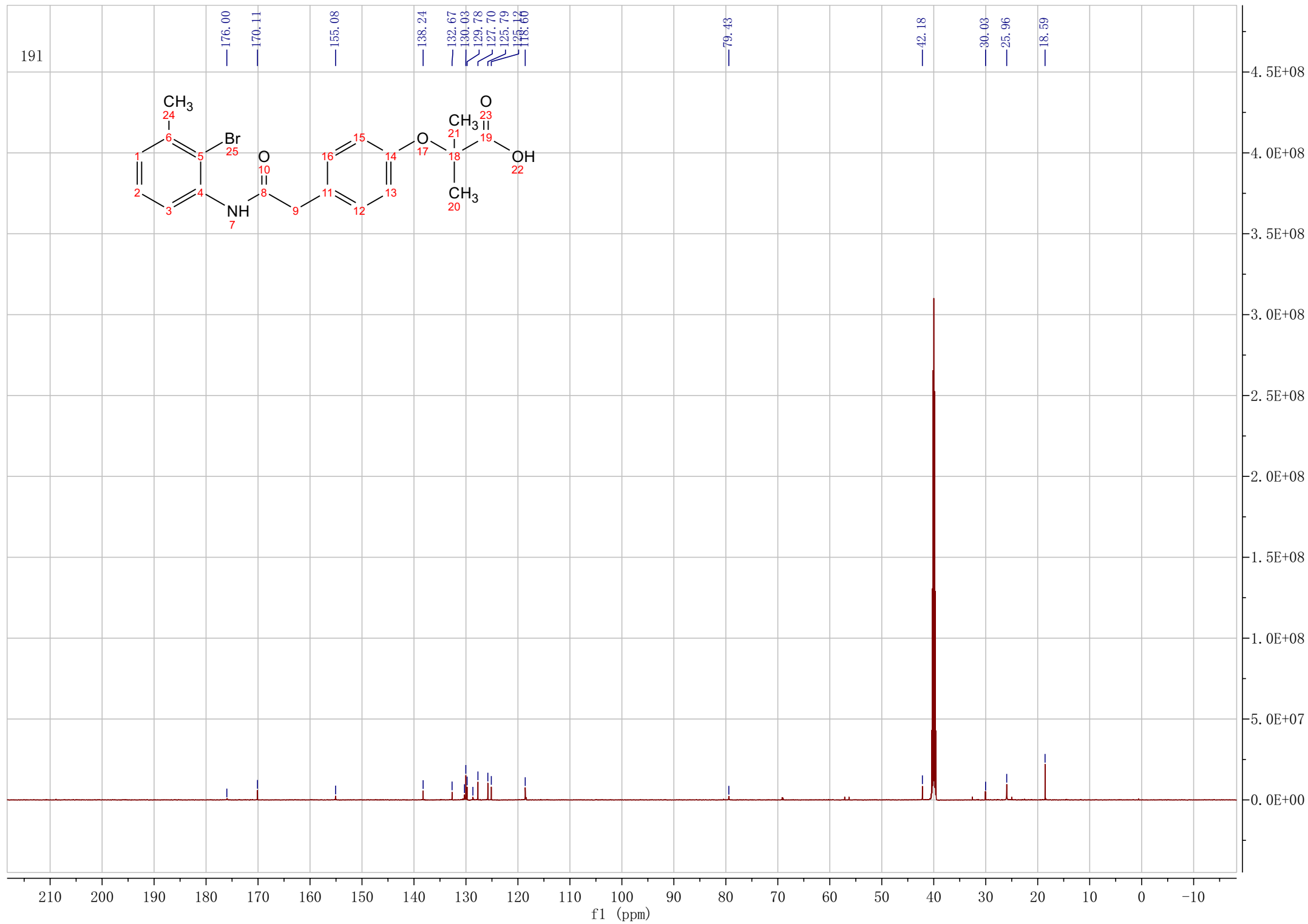

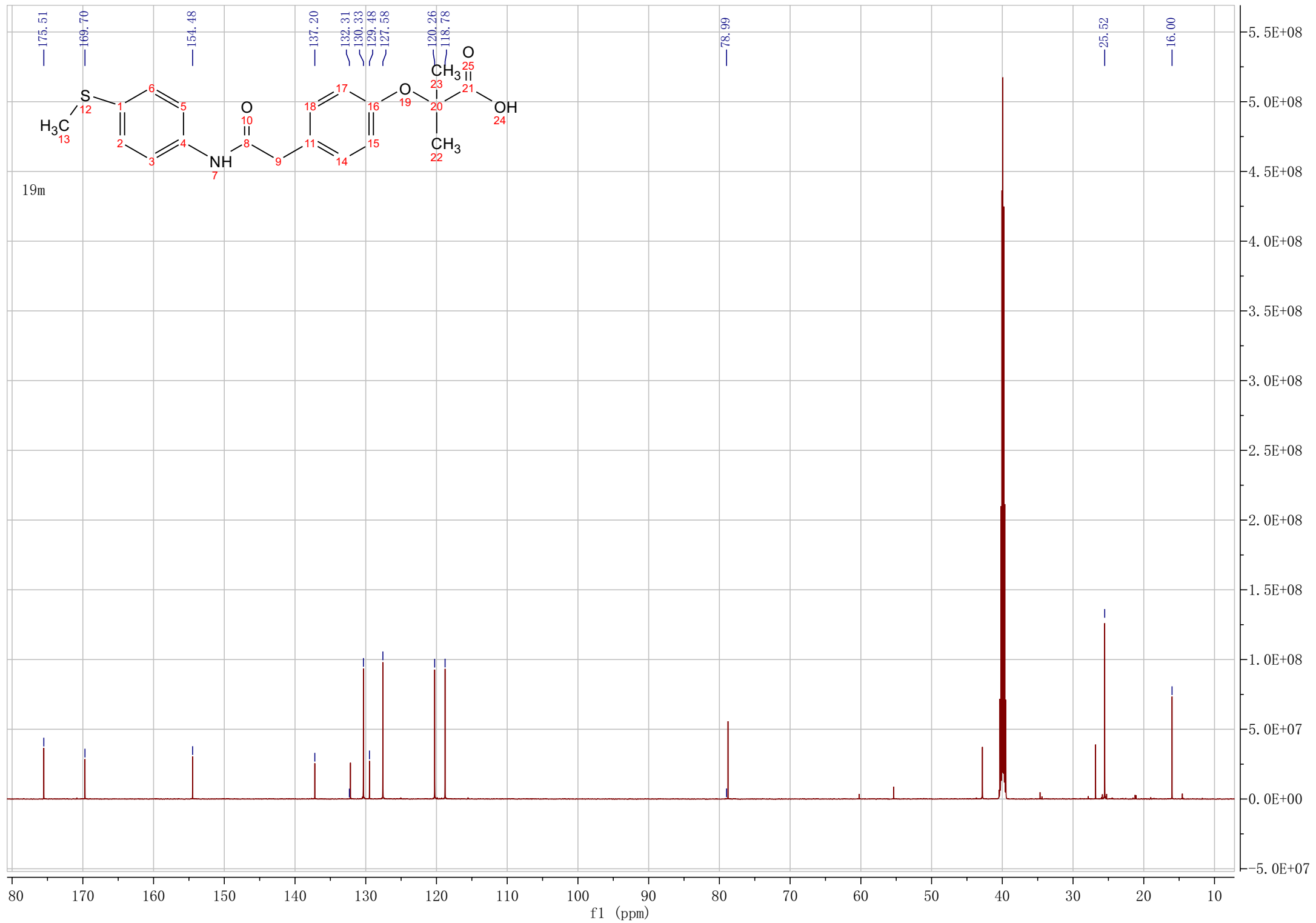

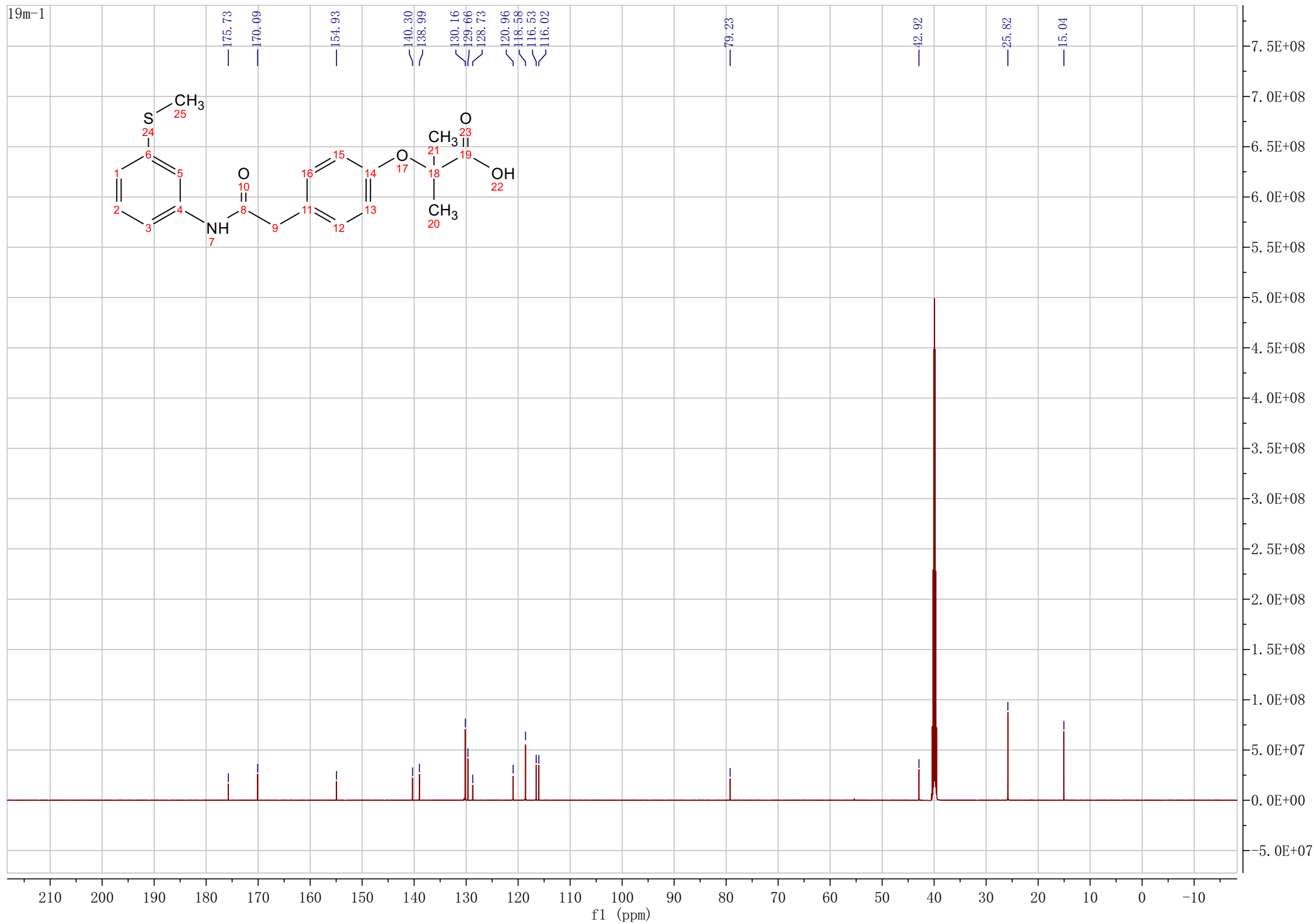

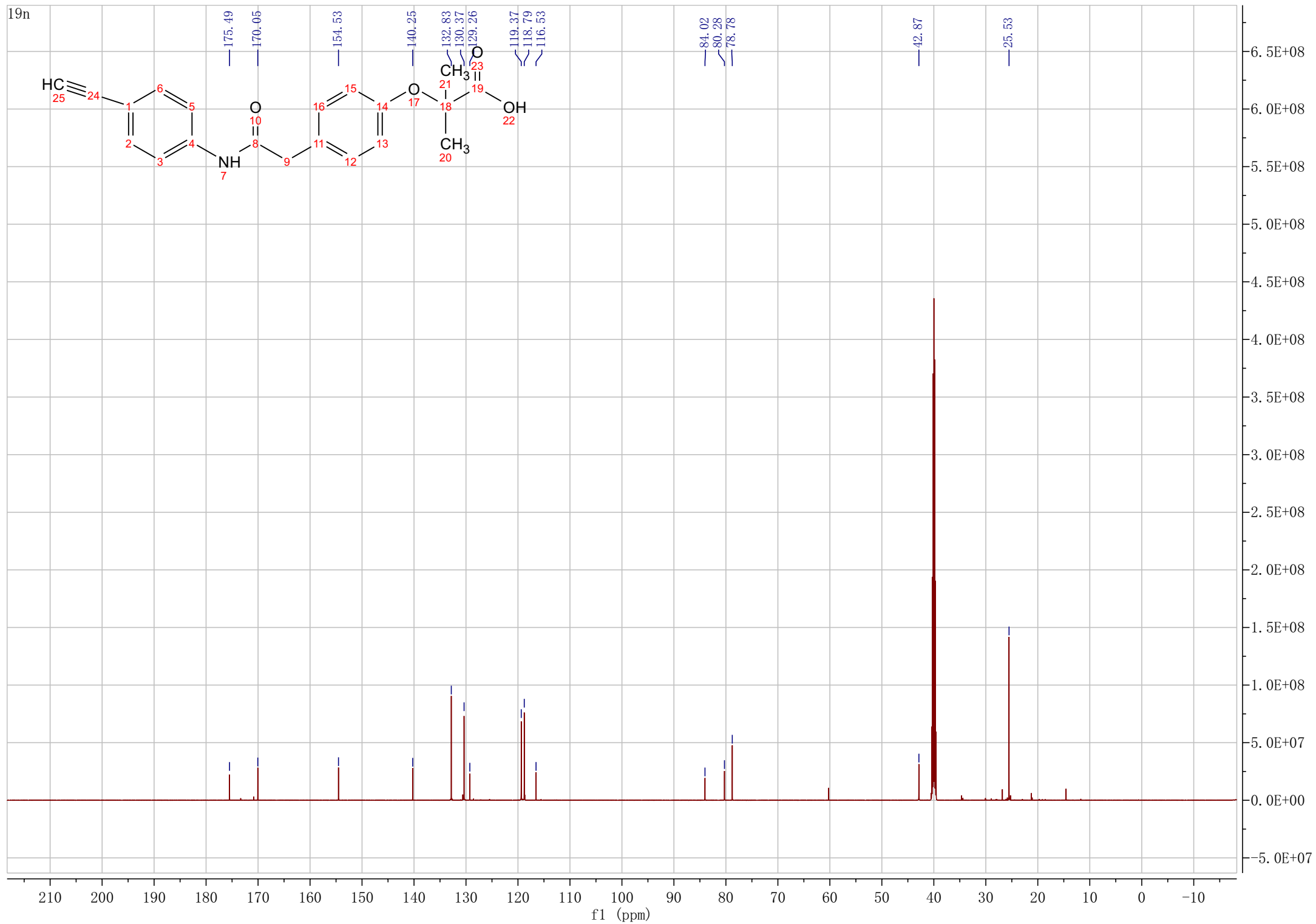

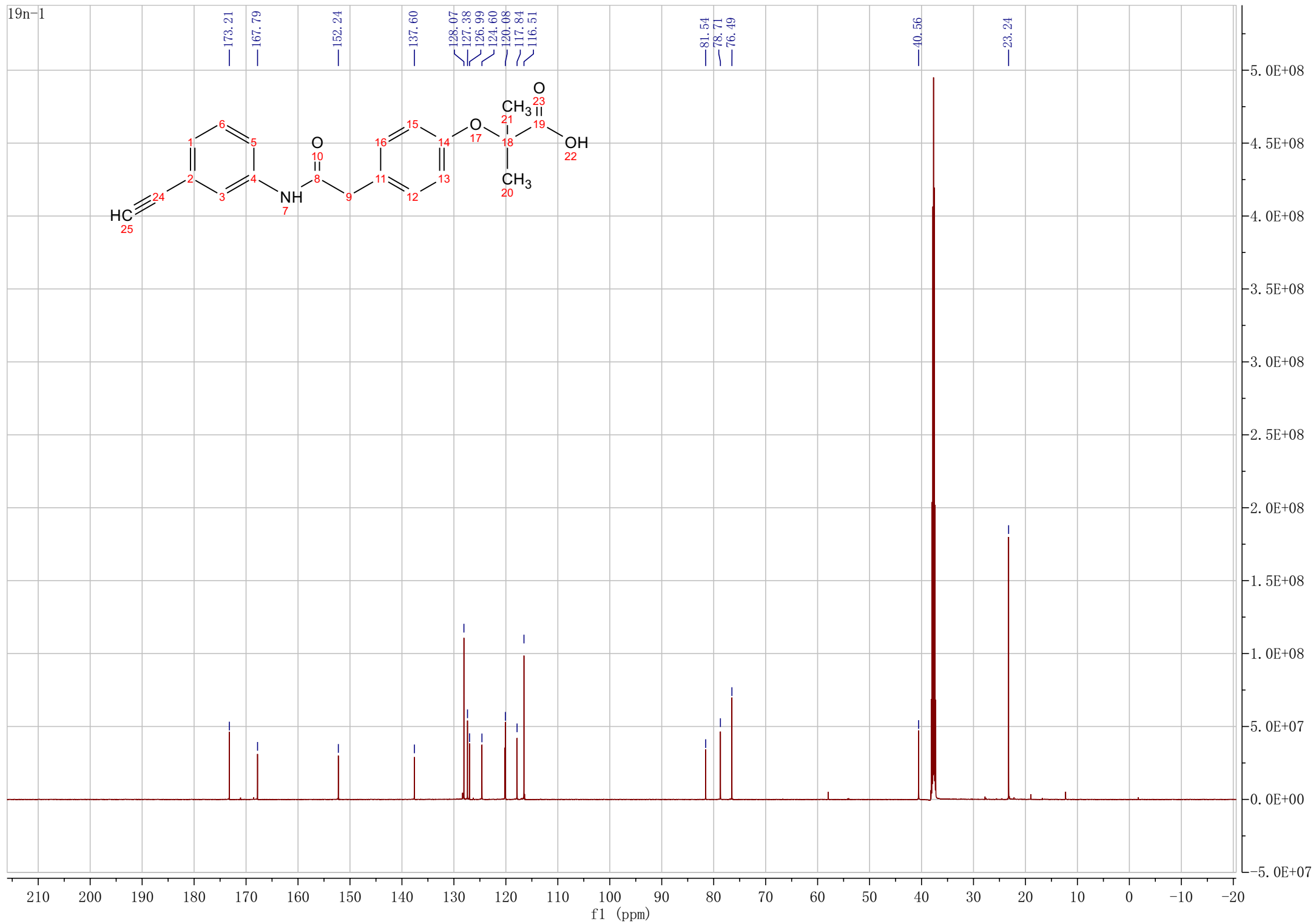

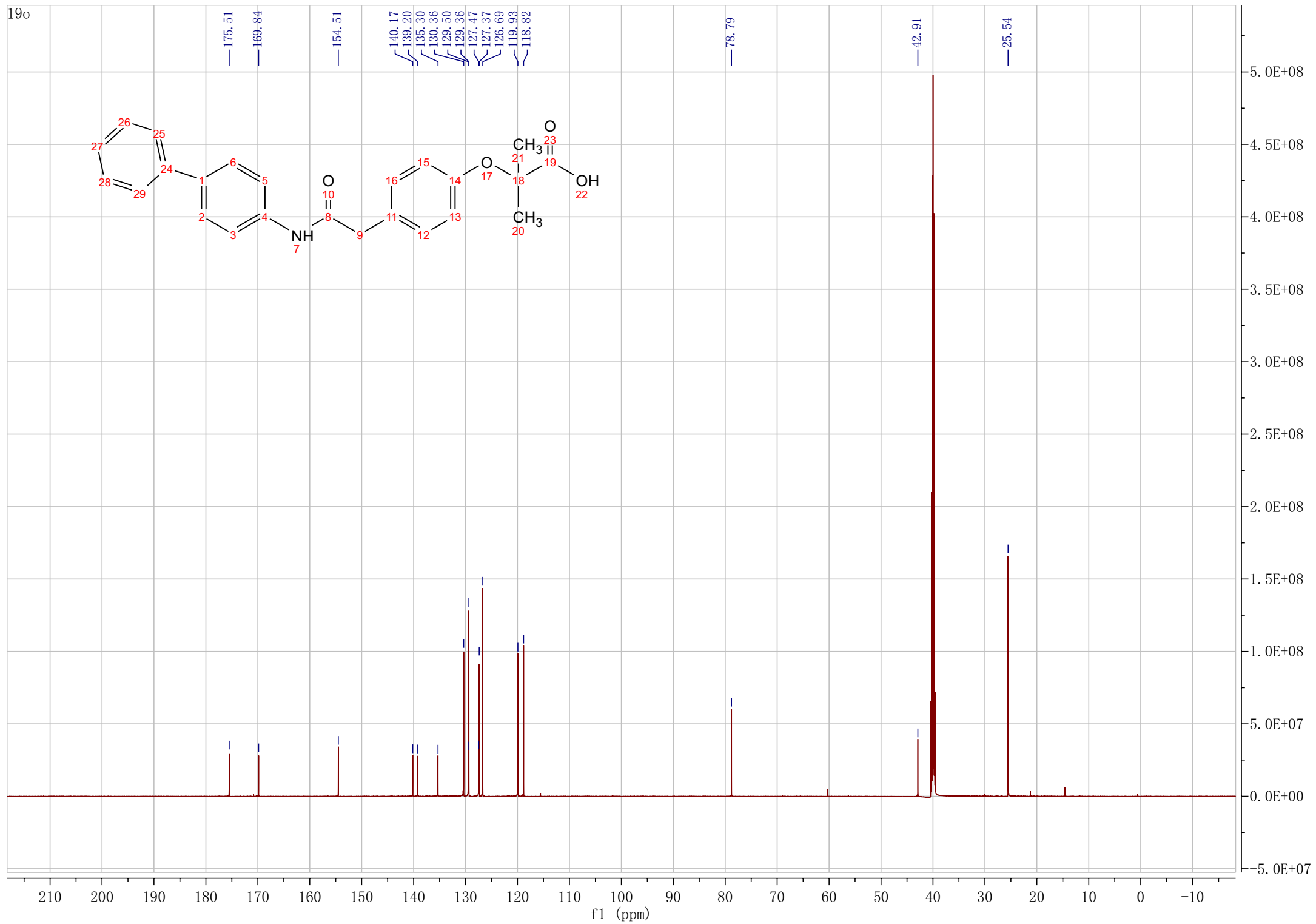

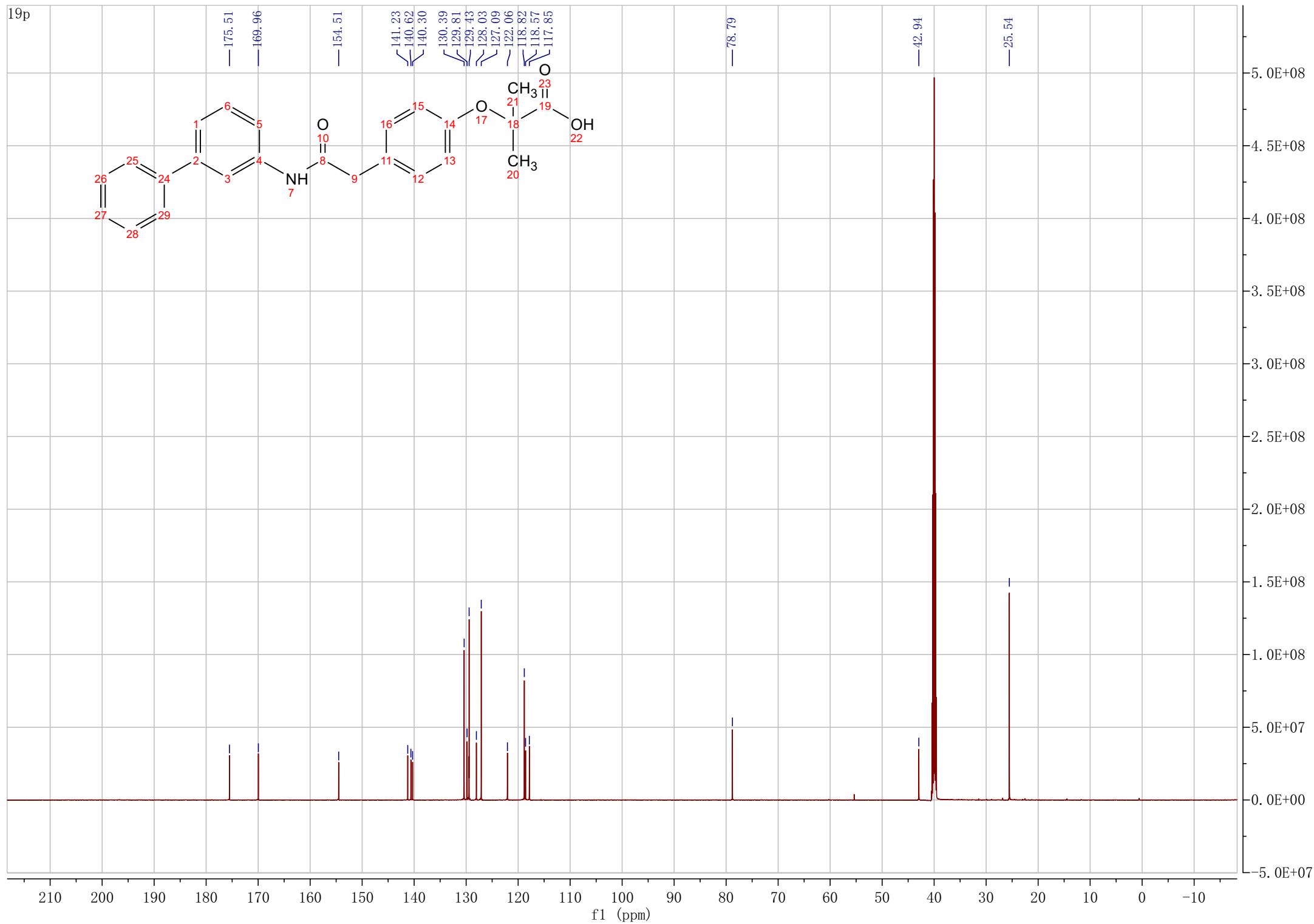

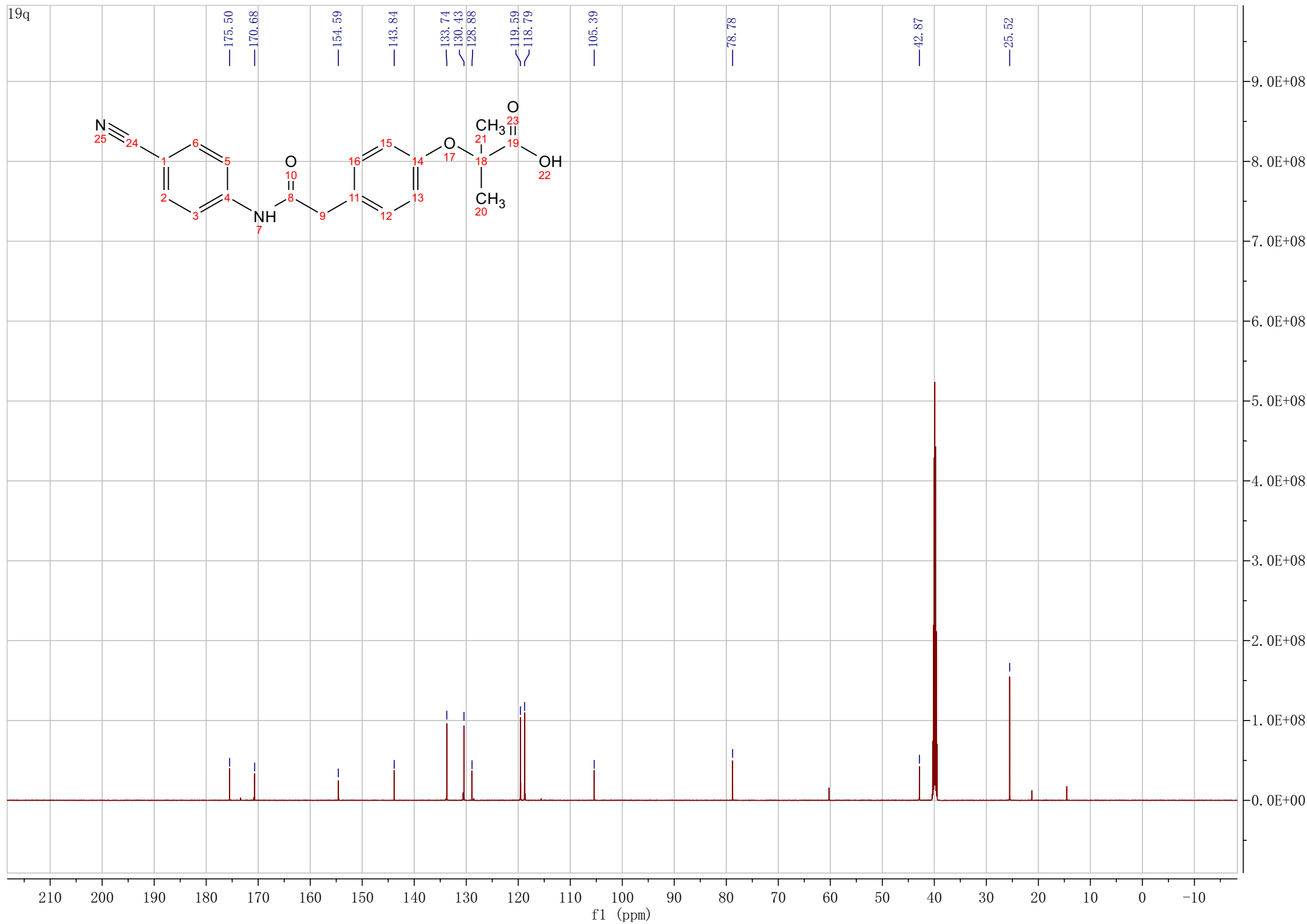

19r

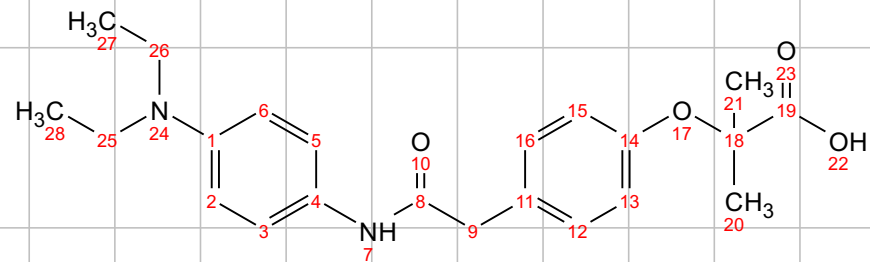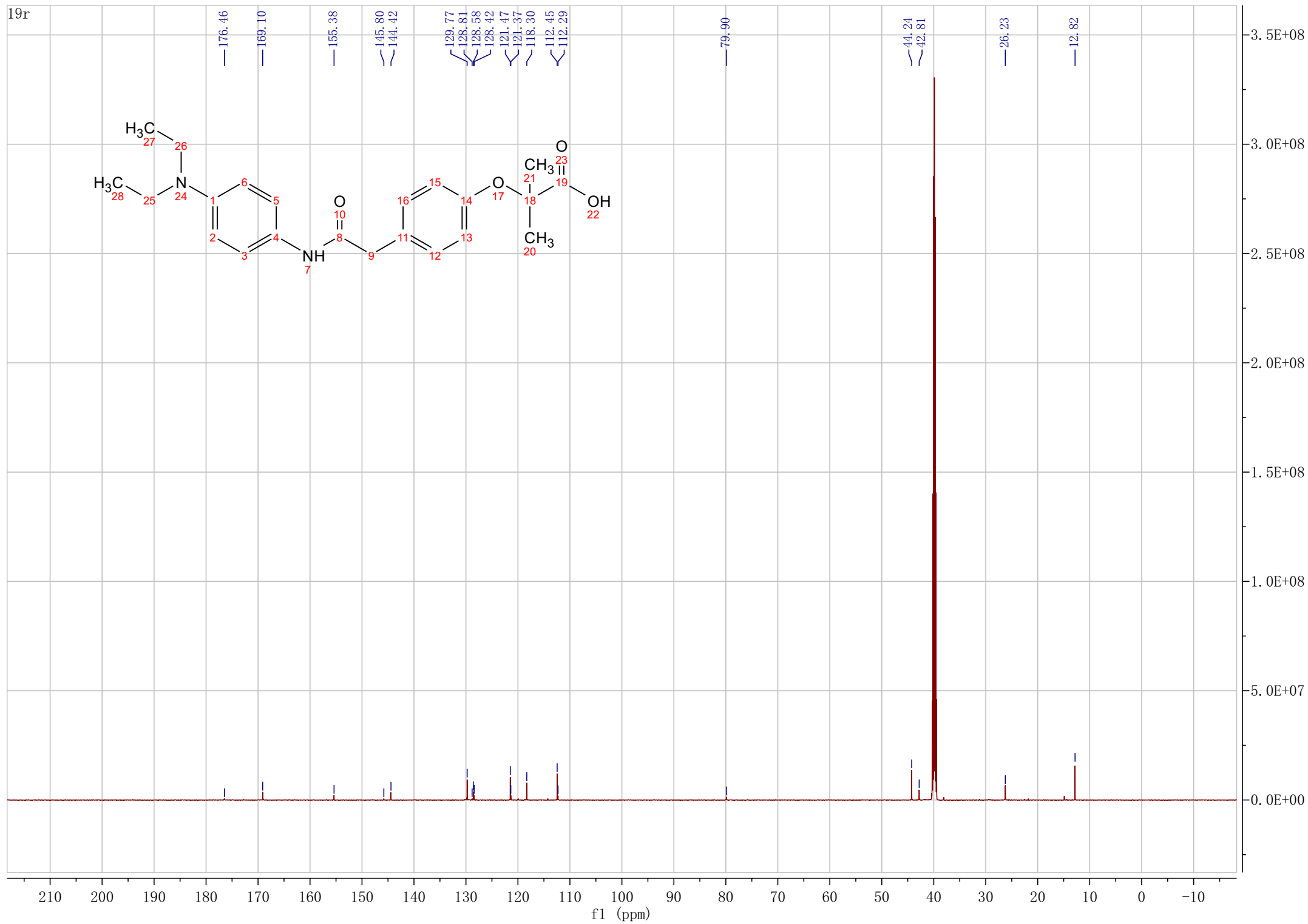

19s

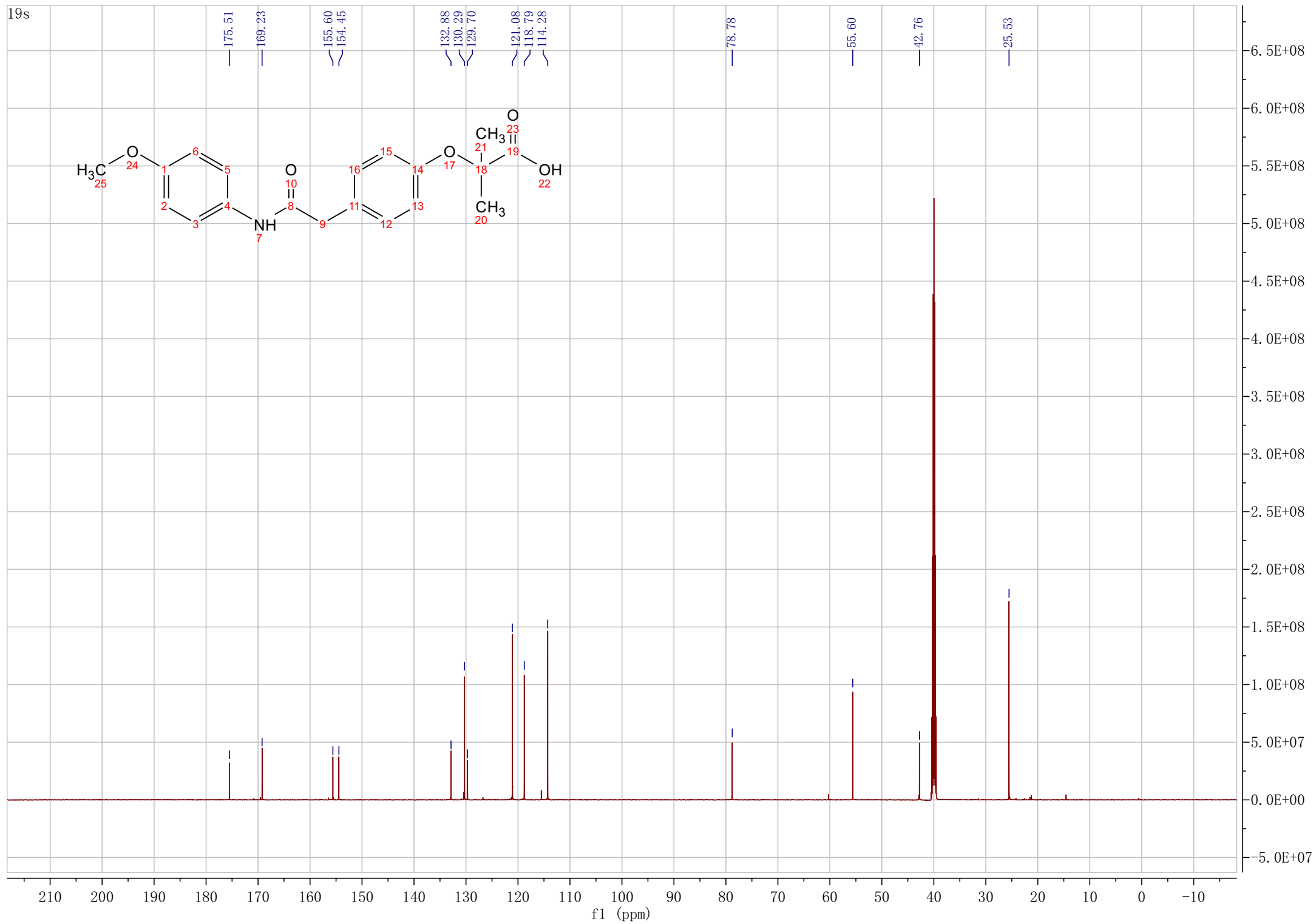

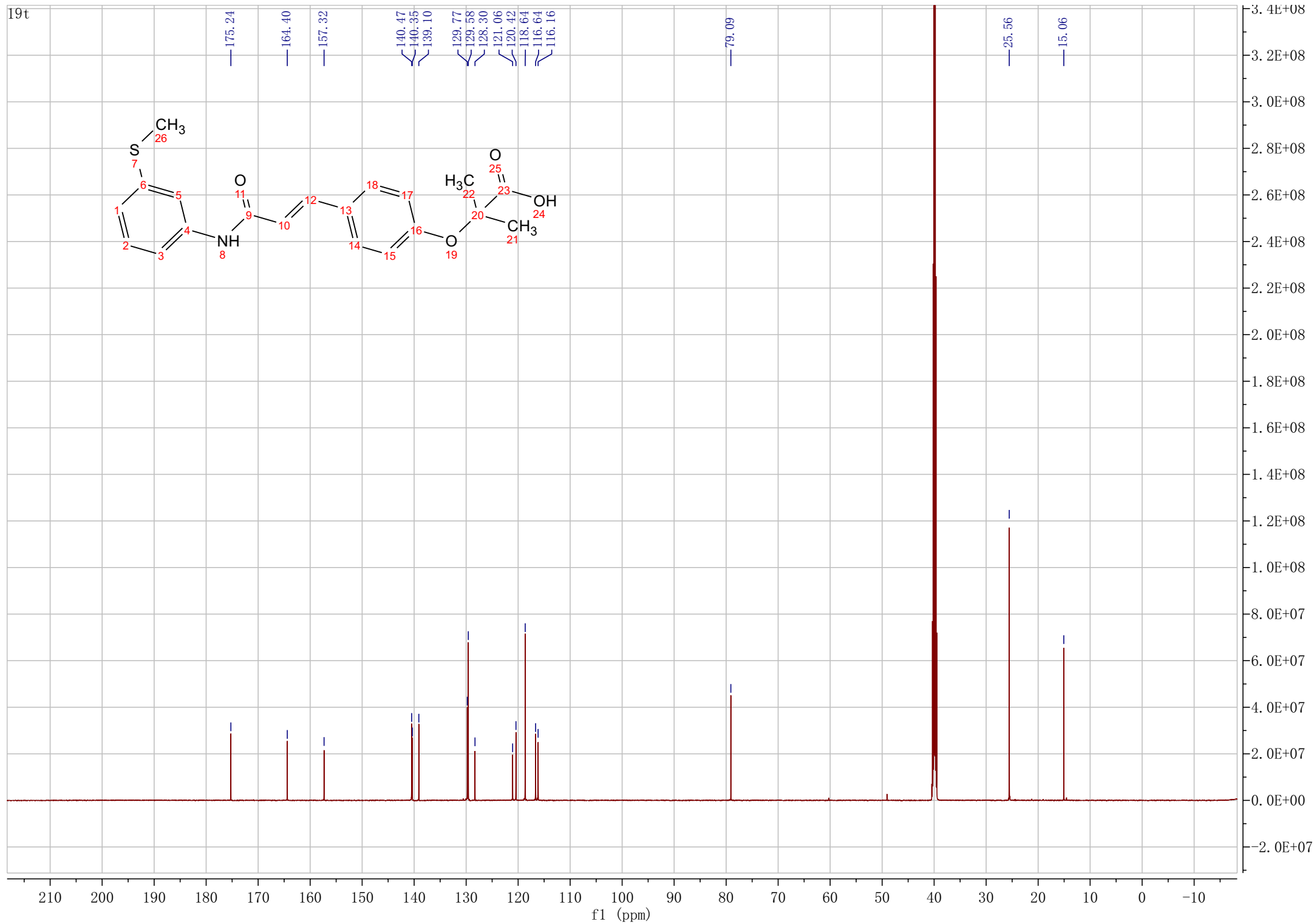

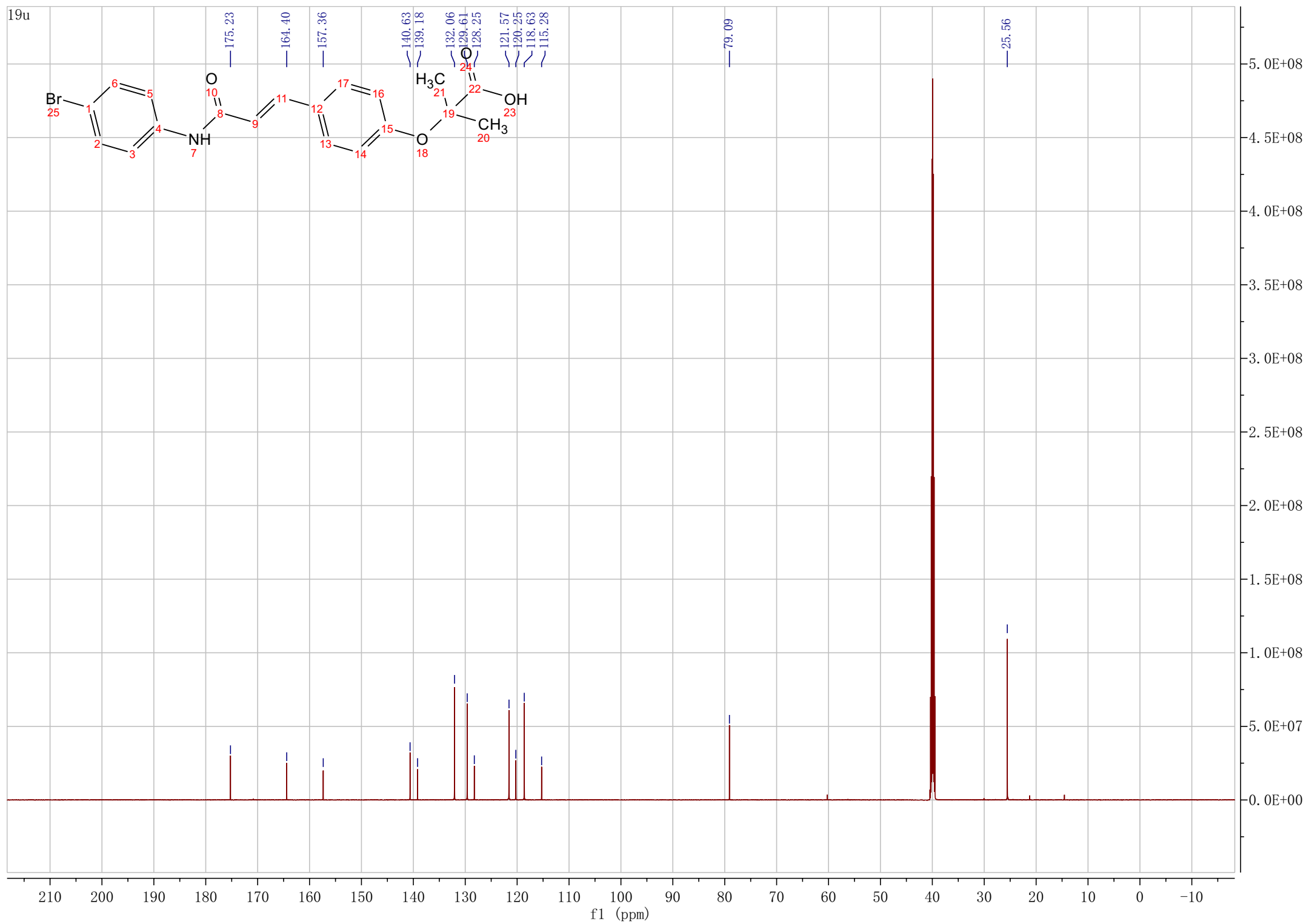

19v

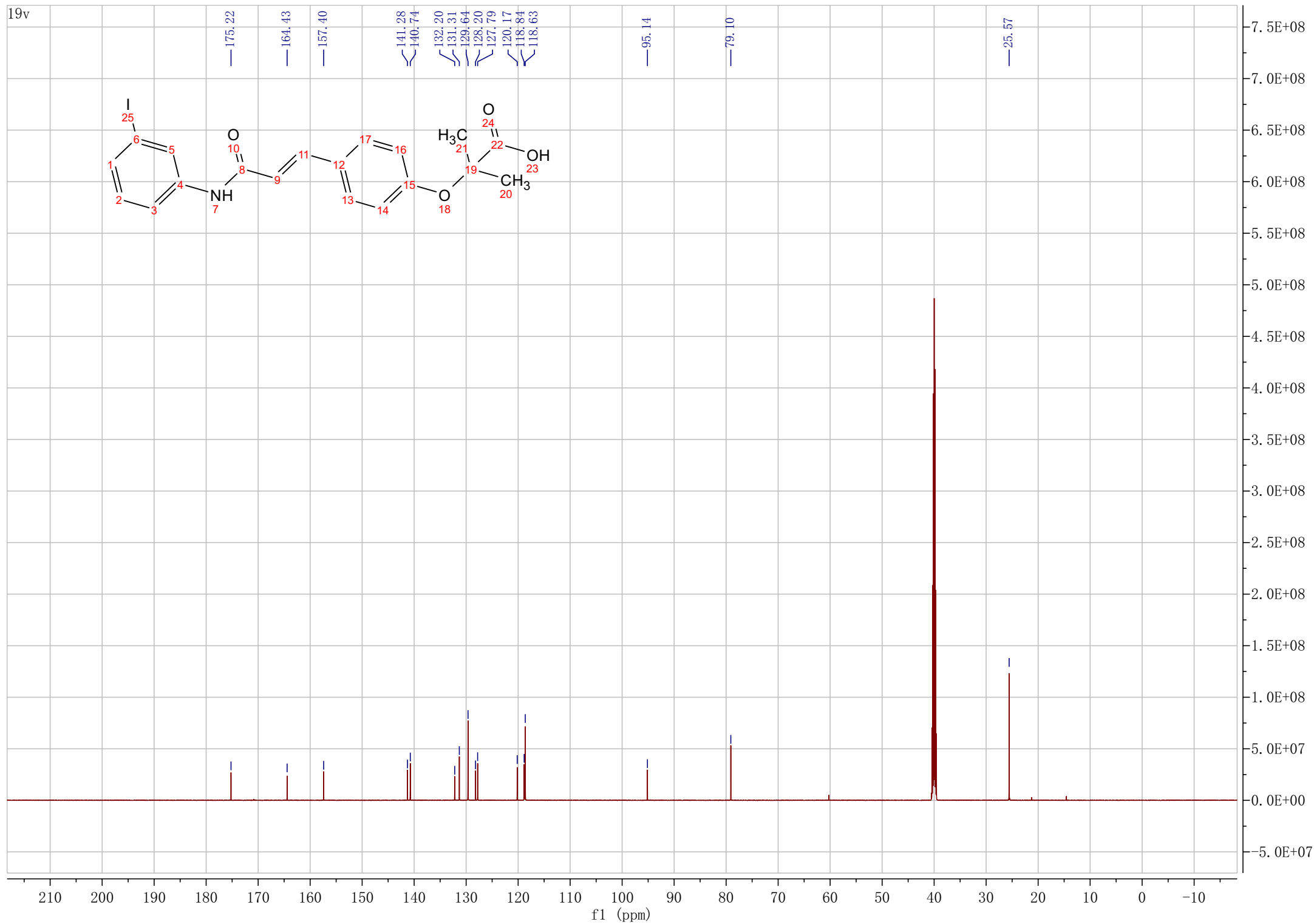

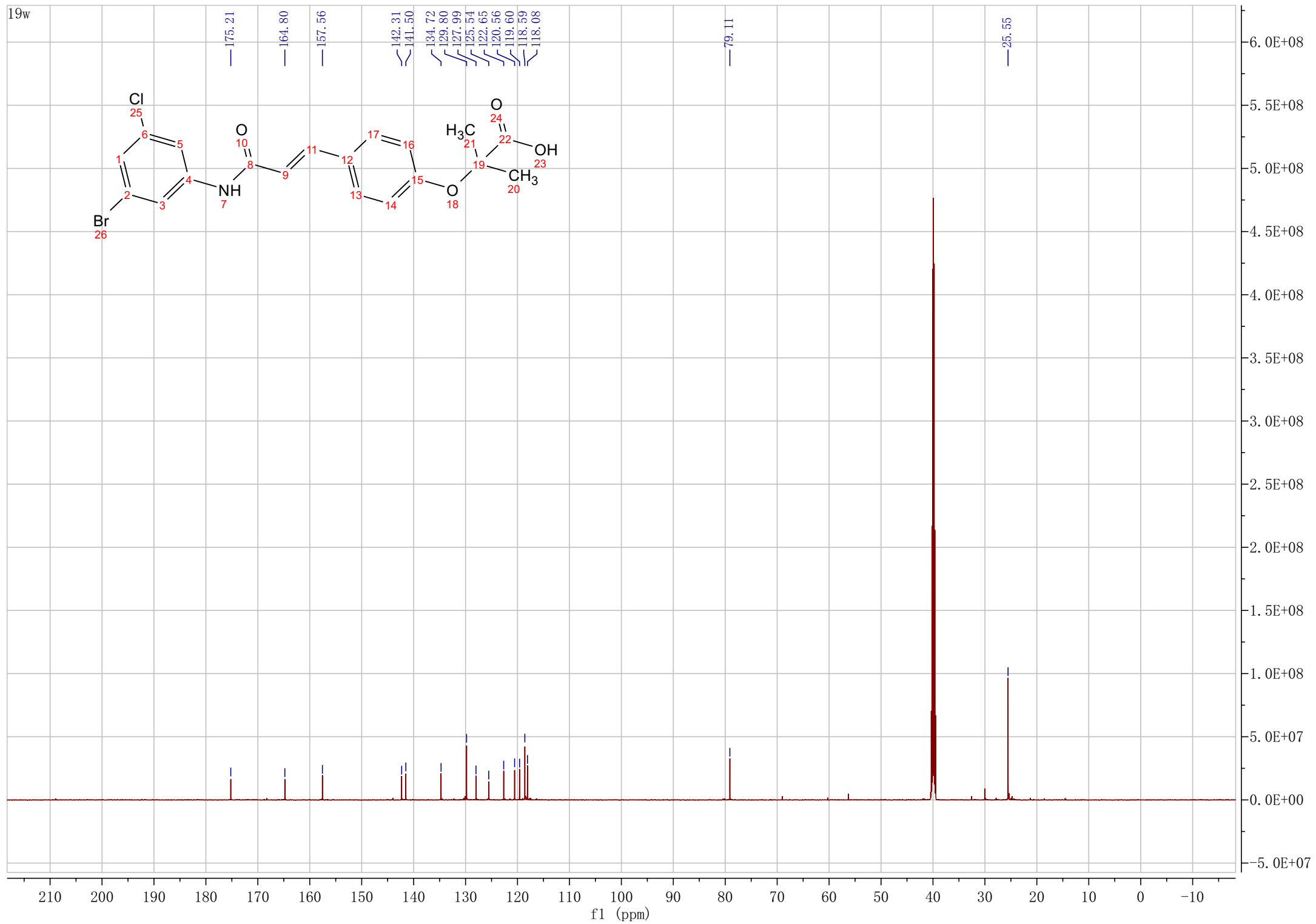

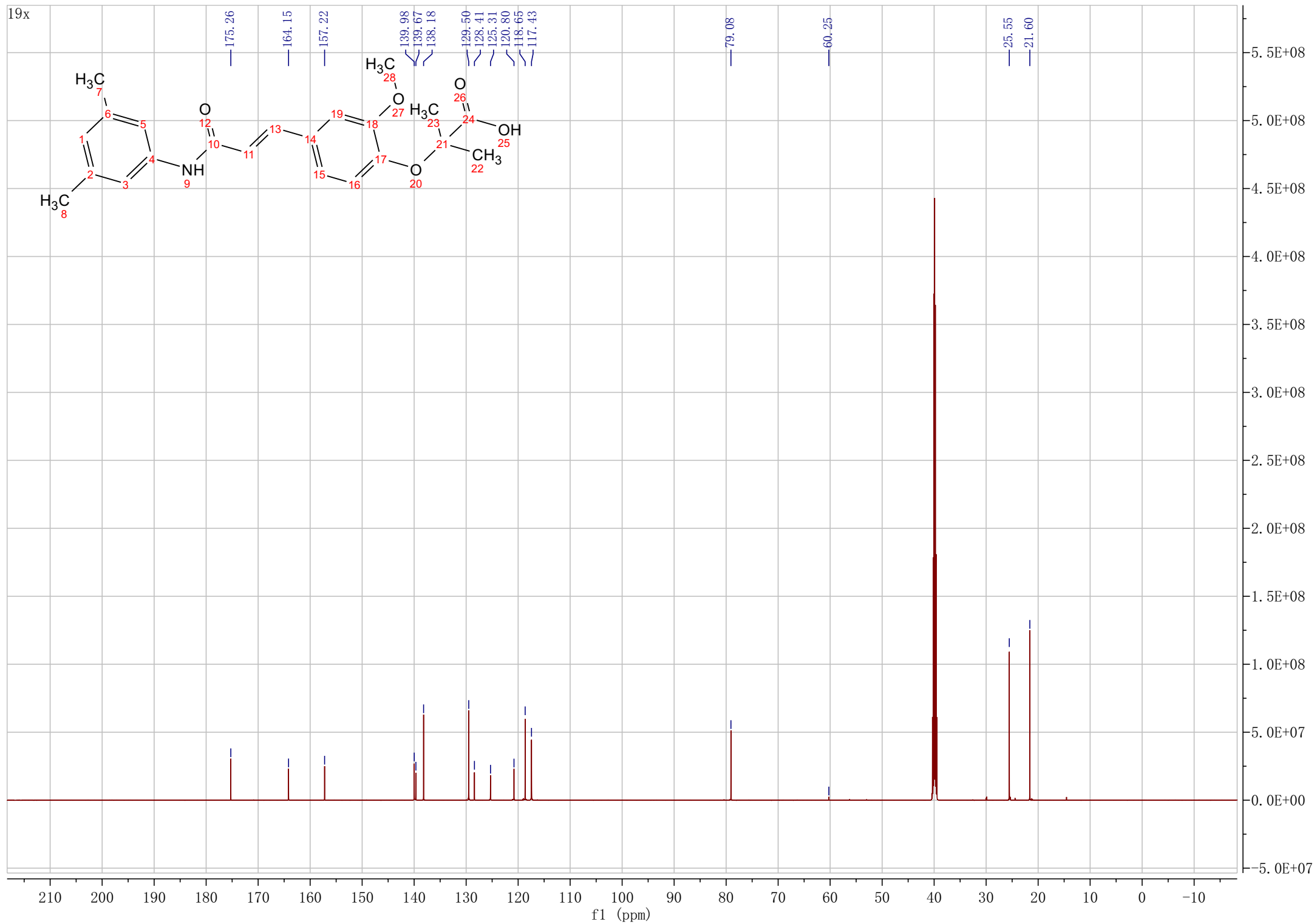

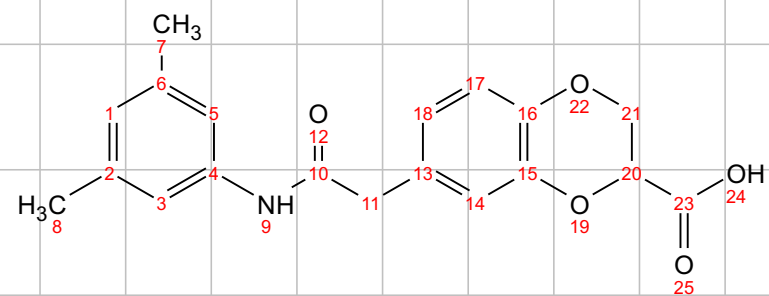

168.98  
168.45  
141.53  
141.10  
138.47  
137.03  
129.02  
124.10  
121.76  
121.11  
116.78  
116.27  
116.08

70.76

64.17

41.98

20.48

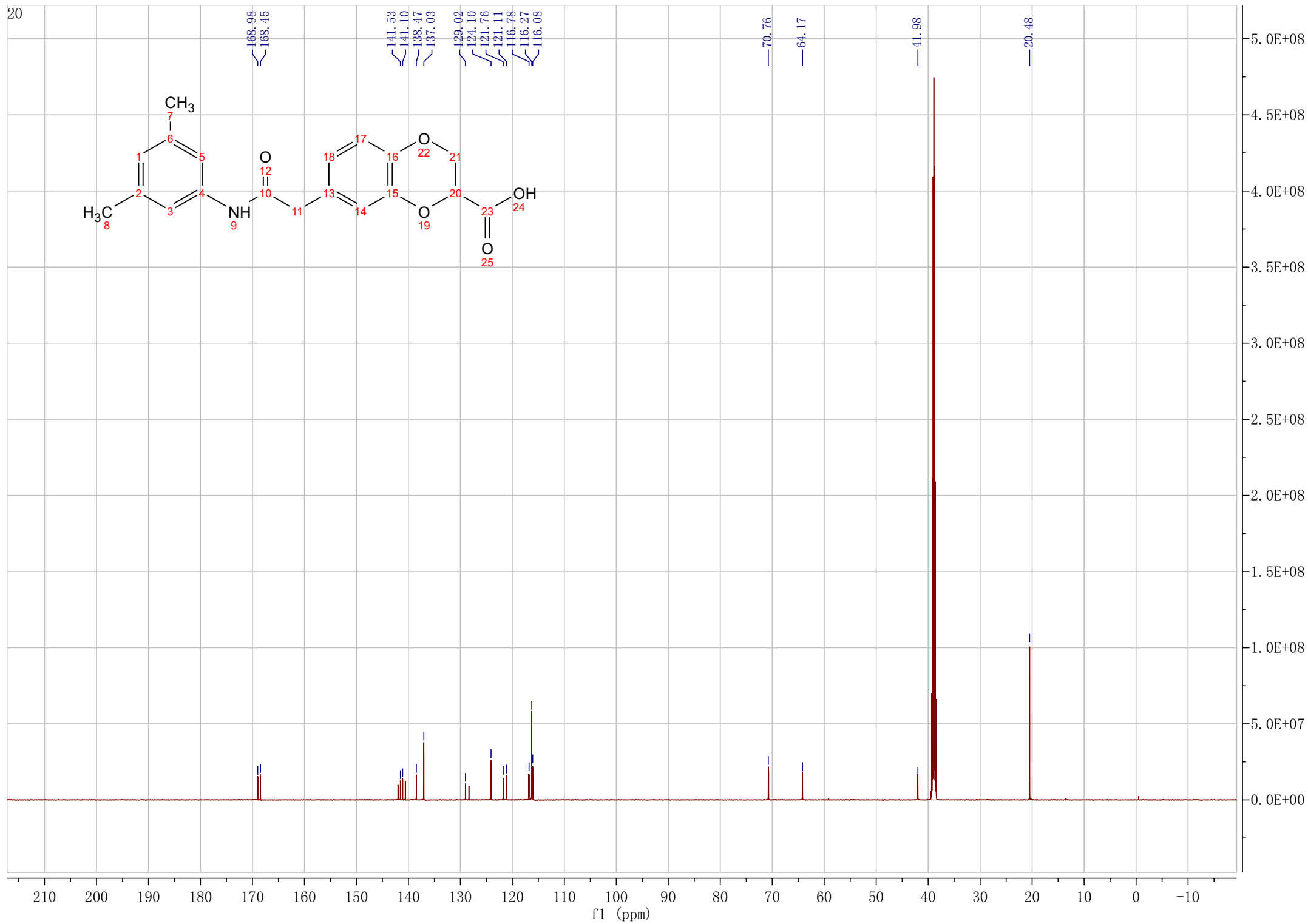

24a

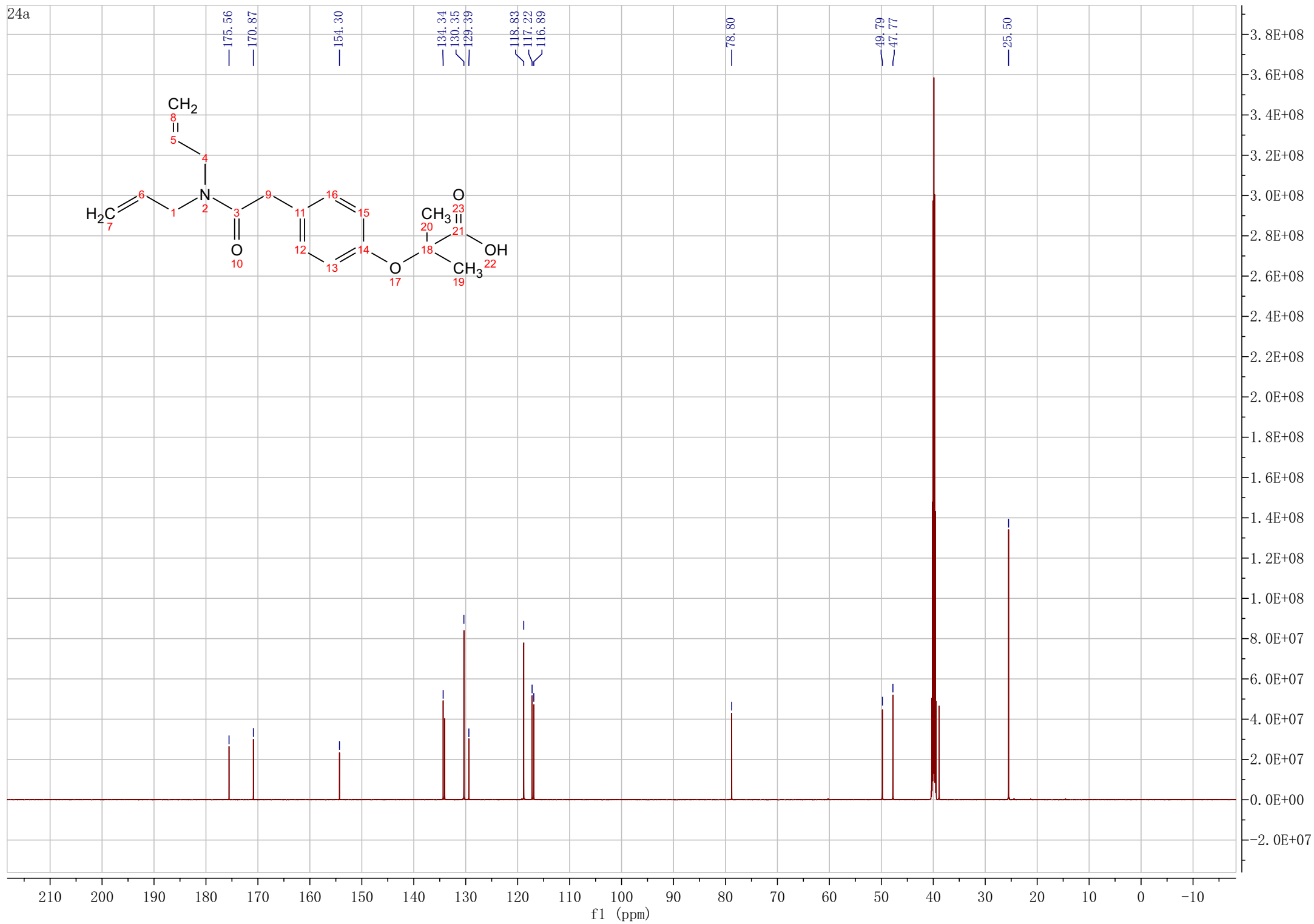

24b

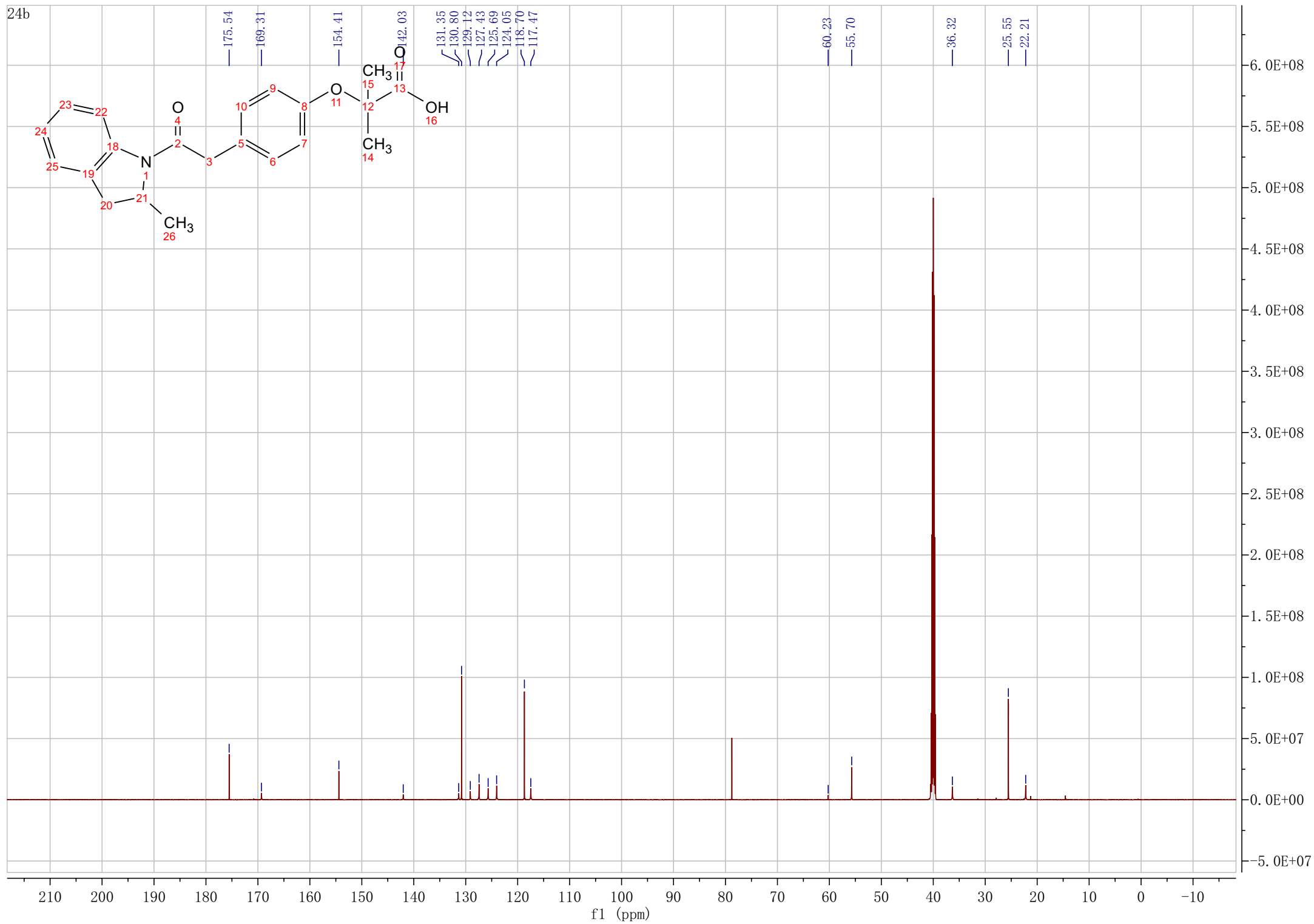

24c

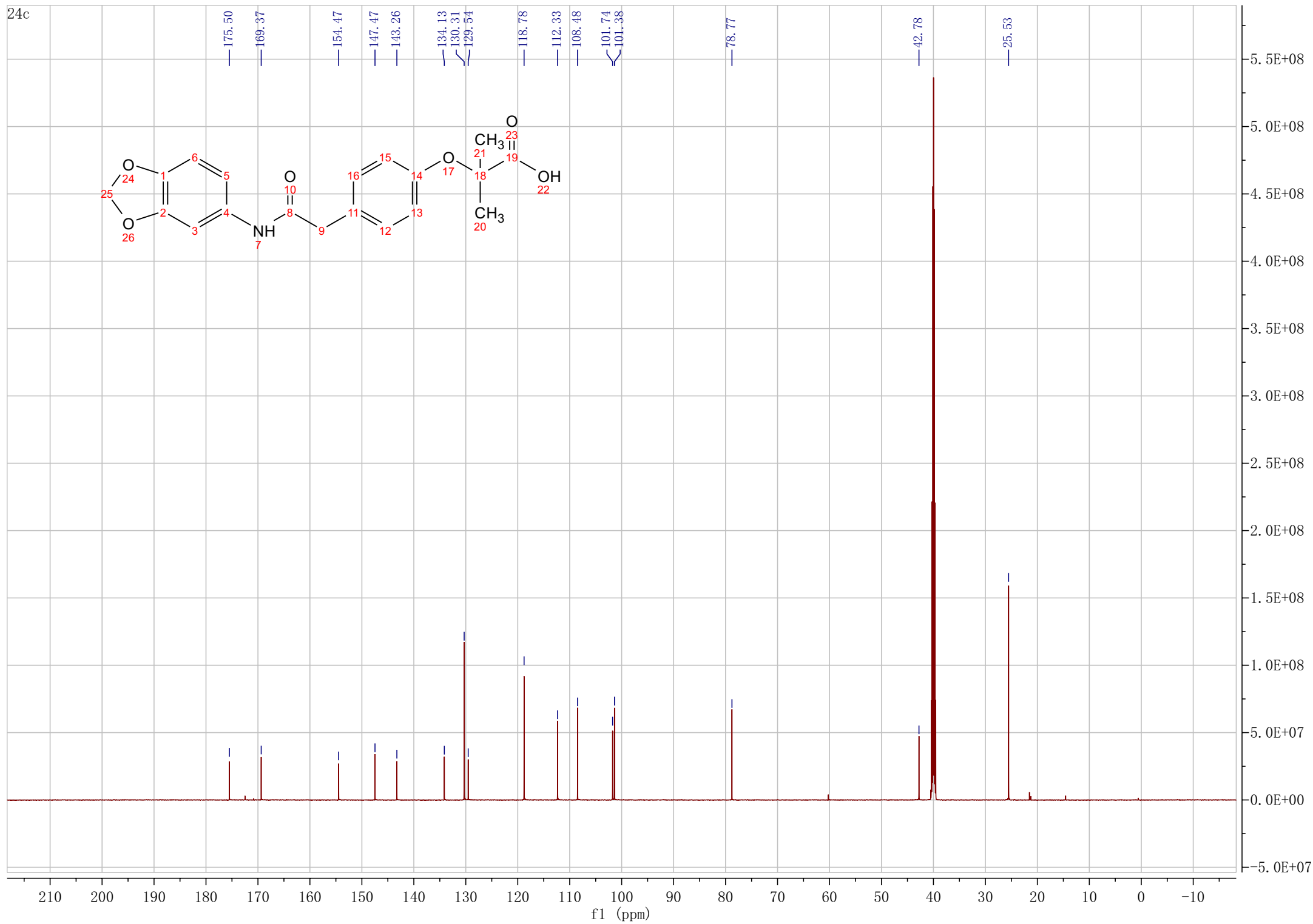

24d

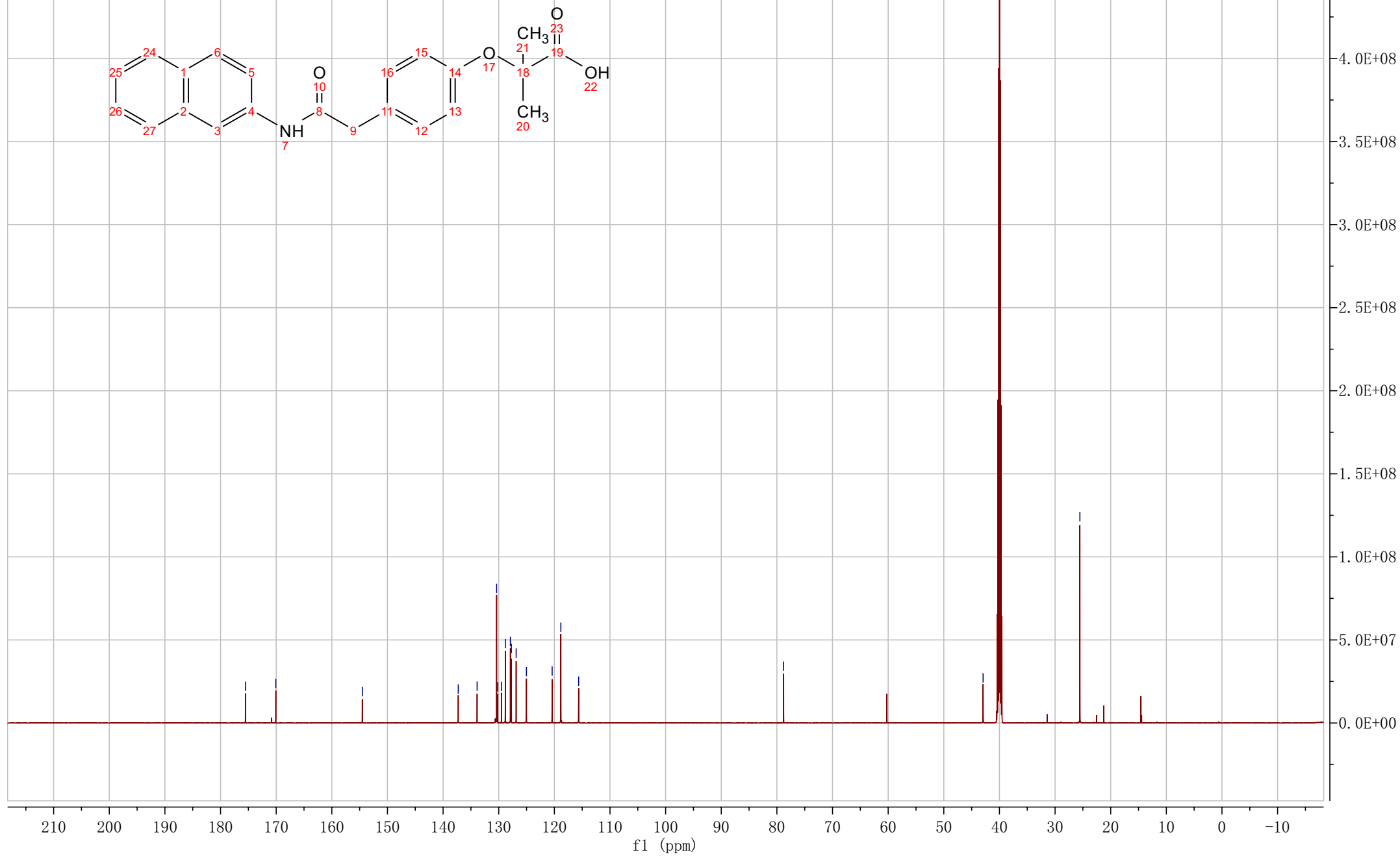

24e

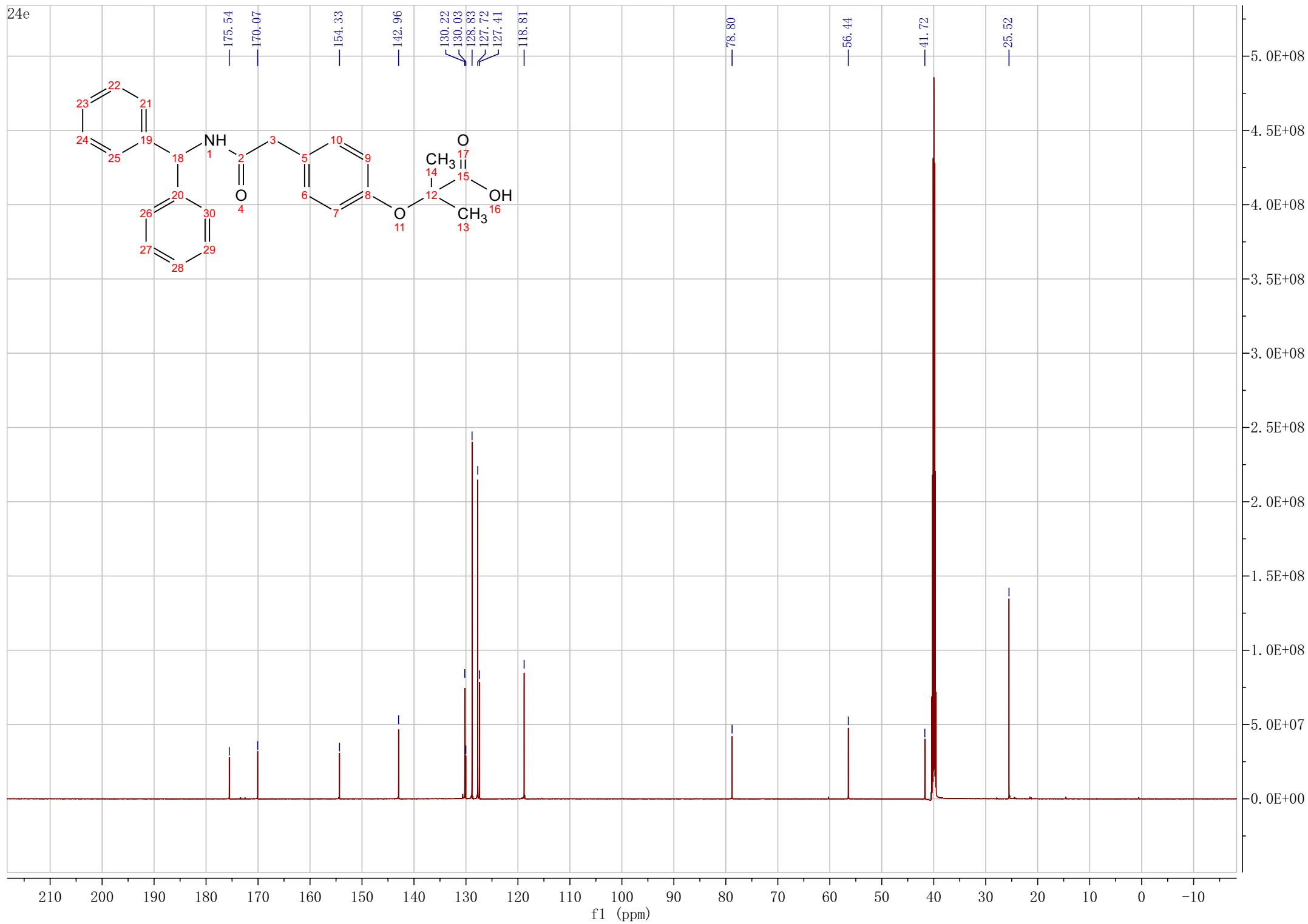

24f

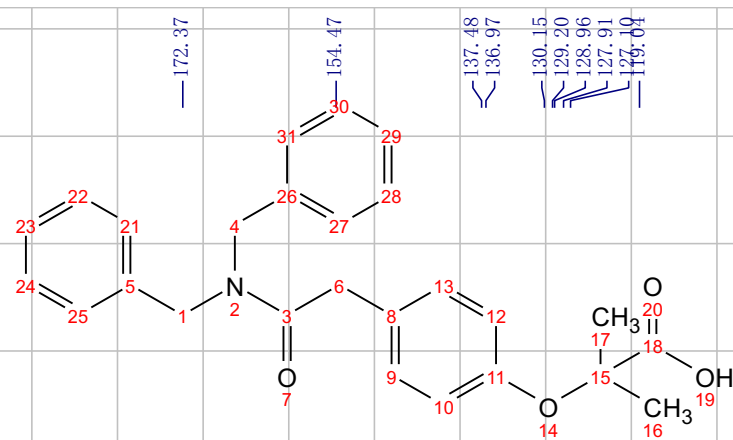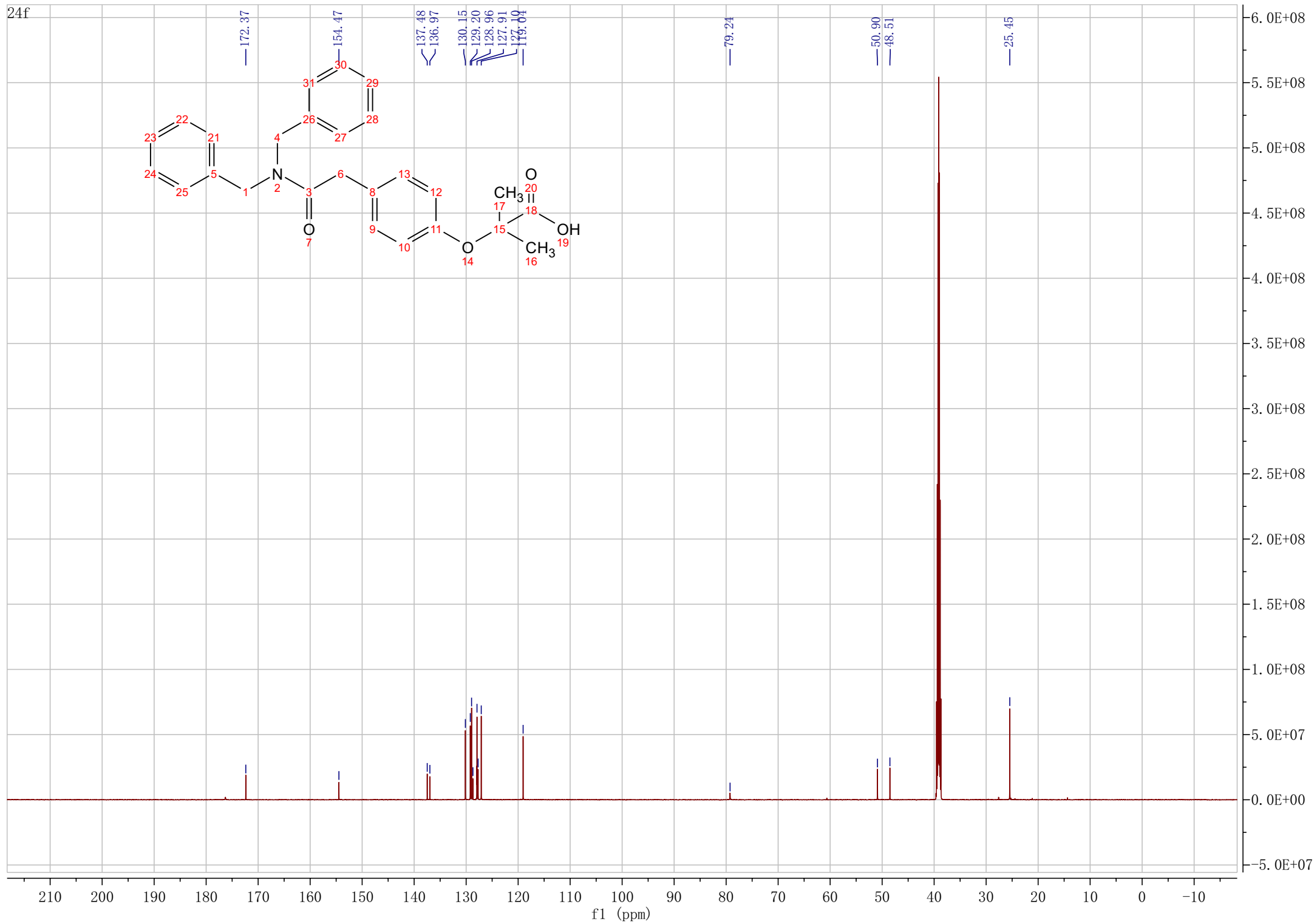

24g

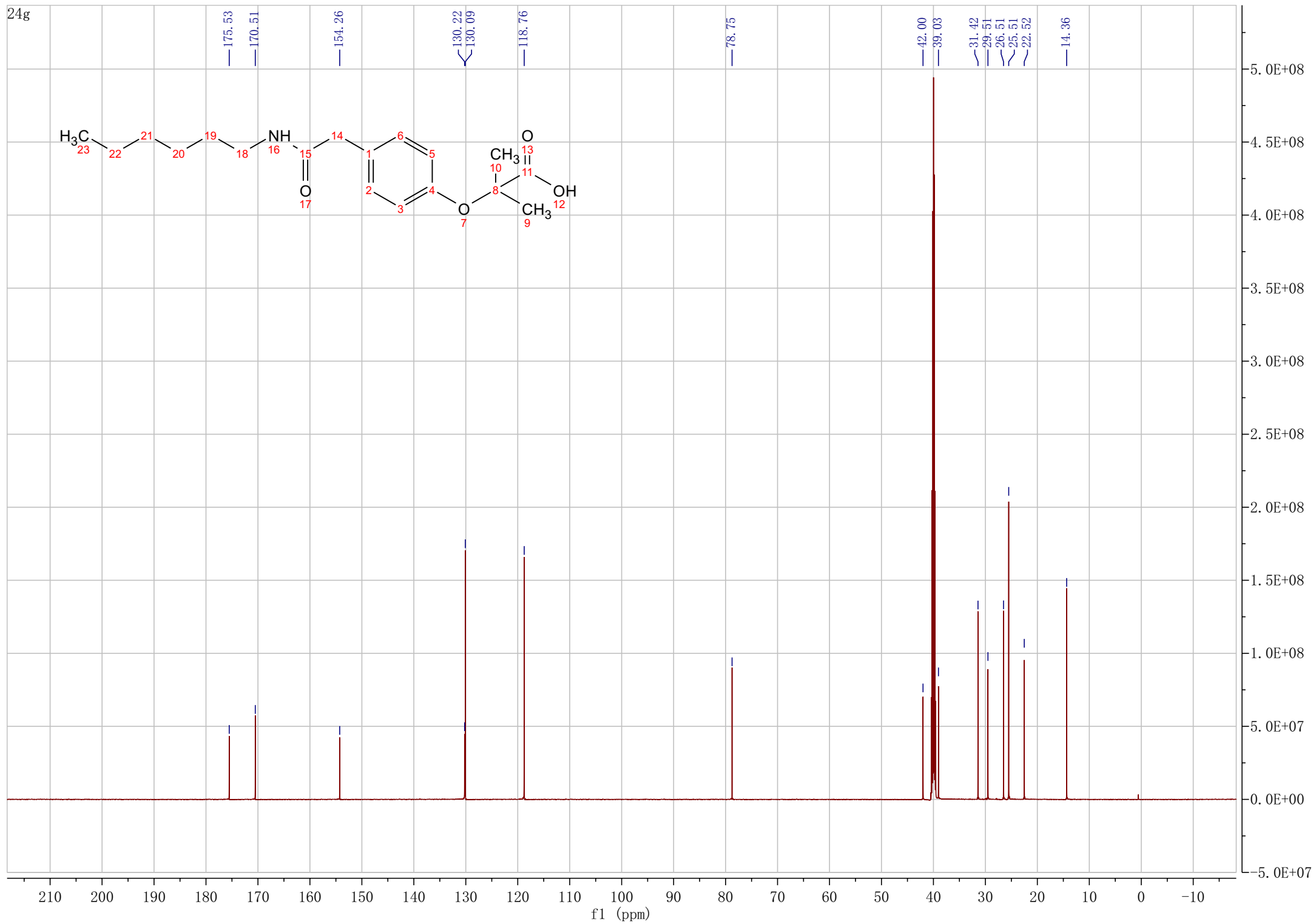

24h

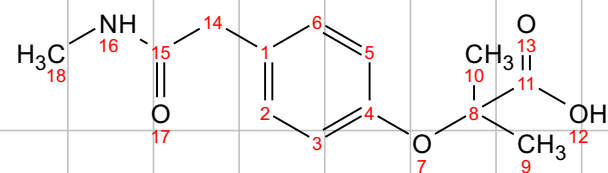

176.04

171.35

154.91

129.89

129.10

118.50

79.39

60.24

26.07

4.0E+08

3.5E+08

3.0E+08

2.5E+08

2.0E+08

1.5E+08

1.0E+08

5.0E+07

0.0E+00

210

200

190

180

170

160

150

140

130

120

110

100

90

80

70

60

50

40

30

20

10

0

-10

f1 (ppm)

Supplement: Supplementary file 1 [file molecules-27-02428-s001.zip › Figure S3.pdf]
